# Bilirubin Improves Gap Junction to Alleviate Doxorubicin-Induced Cardiotoxicity by Regulating AMPK-Axl-SOCS3-Cx43 Axis

**DOI:** 10.3389/fphar.2022.828890

**Published:** 2022-04-25

**Authors:** Siqi Zhang, Yixin Fan, Binbin Zheng, Yu Wang, Chen Miao, Yue Su, Kun Li, Yan E., Xueli Wang, Xueming He, Xuefeng Wu, Chenjie Xu, Yulin Tang, Wen-Tao Liu, Xiangqing Kong, Liang Hu

**Affiliations:** ^1^ Department of Cardiology, The First Affiliated Hospital of Nanjing Medical University, Nanjing, China; ^2^ Department of Pharmacy, Sir Run Run Hospital, Nanjing Medical University, Nanjing, China; ^3^ Jiangsu Key Laboratory of Neurodegeneration, Department of Pharmacology, Nanjing Medical University, Nanjing, China; ^4^ Department of Pharmacy, Xinghua People’s Hospital, Taizhou, China; ^5^ Department of Pathology, The First Affiliated Hospital of Nanjing Medical University, Nanjing, China; ^6^ Center for Clinical Research and Translational Medicine, The Affiliated Lianyungang Oriental Hospital of Kangda College of Nanjing Medical University, Lianyungang, China; ^7^ State Key Laboratory of Pharmaceutical Biotechnology, School of Life Sciences, Nanjing University, Nanjing, China; ^8^ Department of Anesthesiology and Pain, Nanjing First Hospital, Nanjing Medical University, Nanjing, China

**Keywords:** doxorubicin, bilirubin, cardiotoxicity, Cx43 gap junction, AMPK, SOCS3

## Abstract

Doxorubicin induces severe cardiotoxicity, accompanied by the high level of bilirubin in the blood. The conventional wisdom is that bilirubin is considered as a marker of liver damage. By contrast, here we aim to explore the potential protective effect of bilirubin on doxorubicin-induced cardiotoxicity, and investigate the mechanism for drug development. Doxorubicin was used to establish cardiotoxicity model *in vitro* and *in vivo*. The electrocardiogram (ECG), echocardiography and molecular biological methods were used to detect the effects of bilirubin on doxorubicin-induced cardiotoxicity. Consecutive intraperitoneal injection of bilirubin for 7 days significantly attenuated doxorubicin-induced arrhythmia, prolonged survival time and reduced the levels of aspartate aminotransferase (AST), lactate dehydrogenase (LDH), creatine kinase MB (CK-MB) and α-hydroxybutyrate dehydrogenase (α-HBDH) in mice. Bilirubin also markedly inhibited doxorubicin-induced phosphorylation of c-Jun N-terminal kinase (JNK) and connexin 43 (Cx43), and improved gap junction function *in vitro* and *in vivo*. In addition, bilirubin activated adenosine 5′-monophosphate (AMP)-activated protein kinase (AMPK) and induced suppressor of cytokine signaling 3 (SOCS3) expression, which was abolished by Axl inhibition. Moreover, pretreatment with AMPK agonist or AMPK inhibitor could mimic or abolish the cardioprotective effect of bilirubin on H9C2 cells *in vitro*, respectively. Altogether, bilirubin upregulates gap junctions’ function to protect against doxorubicin-induced cardiotoxicity by activating AMPK-Axl-SOCS3 signaling axis. We enrich the physiological function of bilirubin, and provide theoretical support for drug development.

## Introduction

Chemotherapeutic cardiotoxicity is a difficult problem that cannot be ignored, and the cardiotoxicity of doxorubicin, a representative of anthracycline, is particularly prominent (Vejpongsa and Yeh, 2014). Previous study has found that doxorubicin could induce the destruction of myocardial mitochondria and produce a large amount of ROS, inducing the apoptosis and necrosis of cardiomyocytes (Zhang et al., 2012; Songbo et al., 2019), and induce the disorder of cardiac conduction system and arrhythmia. Doxorubicin significantly induced acute cardiotoxicity in beagle dogs characterized by conduction abnormalities, such as decreased heart rate, ST segment elevation, QT intervals prolongation and arrhythmia (Xin et al., 2011). Electrocardiogram (ECG) monitoring also found doxorubicin treated-rats showed bradycardia, QRS widening and prolongation of both QT and PR intervals (Botelho et al., 2019). Nevertheless, the mechanism of doxorubicin-induced cardiac conduction abnormalities is not very clear. It is particularly important to explore the mechanism of doxorubicin-induced cardiotoxicity and find new therapeutic strategies.

Connexins are integral membrane building blocks that form gap junction, enabling direct cytoplasmic exchange of information and substances between adjacent cells, playing an important role in cardiac conduction (Boengler, 2009). In the heart, gap junction mediates the propagation of cardiac action potentials and the maintenance of a regular beating rhythm (Sorgen et al., 2018). Connexin 43 (Cx43), a protein of the connexin family, is closely related to heart conduction (Fontes et al., 2012). Studies have shown that Cx43 gap junction electrical coupling is essential for normal impulse propagation through the heart, it not only coordinates synchronous myocardial contraction, but also couple cardiomyocytes to non-cardiomyocytes, altering the electrophysiological properties of cardiomyocytes. Reduced expression of Cx43 gap junction would lead to reduce intercellular coupling and reduce the conduction velocity (Jongsma and Wilders, 2000; Fontes et al., 2012). Evidences have also shown that chemotherapy can reduce the expression of total Cx43 protein, but upregulated the phosphorylation level of Cx43 (Abdelmohsen et al., 2005). Additionally, chemotherapy induces the expression of pro-inflammatory factors, such as TNF-α and INF-γ, which in turn significantly reduced Cx43 expression (Leaphart et al., 2007; Tang and Fang, 2017). Besides, the stress kinase c-jun N-terminal kinase (JNK), a pivotal regulator of Cx43, could downregulate Cx43 to impair cell-cell communication and promote the development of atrial fibrillation once activated (Yan et al., 2018). Thus, targeting Cx43 inhibition may be helpful for the treatment of doxorubicin-induced cardiotoxicity.

Doxorubicin-induced cardiotoxicity may be a mixed mechanism of ROS-induced myocardial injury and inflammation-induced conduction disorder. Thus, the ideal intervention strategy is to find a safe compound that can not only improve the conduction disorder but also can inhibit inflammatory cell infiltration-induced cardiomyocyte injury. Bilirubin, a compound with strong antioxidant capacity (Ziberna et al., 2016). Previous studies have reported that moderately higher levels of bilirubin within the range considered normal were associated with reduced risk of respiratory disease and all-cause mortality (Horsfall et al., 2011). A fuller understanding of these mechanisms may lead to the potential use of targeted clinical treatments that moderately increase bilirubin levels. Serum concentration of bilirubin was negatively correlated with coronary heart disease (CAD), which indicated that bilirubin may play a new role of protection CAD (Schwertner et al., 1994; Vitek, 2016), but the underlying mechanism is still not clear. Moreover, it is shown that chemotherapy patients were often accompanied by an increase in bilirubin, which was previously thought to be a concomitant phenomenon of chemotherapy-induced liver damage (Damodar et al., 2014). In addition, during cellular stress, the level of bilirubin in the body will also increase, and our previous research has focused on adenosine 5′-monophosphate (AMP)-activated protein kinase (AMPK), the cellular energy stress molecule, involved in inflammation and repair process (Song et al., 2015; Zhang et al., 2017). In the present study, we mainly explore whether bilirubin have cardioprotective effect on doxorubicin-induced cardiotoxicity, and investigate its underlying mechanism.

## Materials and Methods

### Chemicals and Reagents

Bilirubin (purity 99.2%; Catalog No. T2934) was purchased from TargetMol (Washington, MA, United States), doxorubicin was purchased from Shenzhen Main Luck Pharmaceuticals Inc., AICAR (Catalog No. A9978), metformin (Catalog No. PHR1084) and compound C (Catalog No. P5499) were purchased from Sigma-Aldrich (St. Louis, MO, United States). R428 (HY-15150) was purchased from MedChemExpress (Pudong New Area, Shanghai, China). Axl small interfering RNA (siRNA, m; Catalog No. sc-29770) and control siRNA (Catalog No. sc-37007) were purchased from Santa Cruz Biotechnology (Santa Cruz, CA, United States). Antibody for Axl (Catalog No. ab215205), suppressor of cytokine signaling 3 (SOCS3; Catalog No. ab16030) was purchased from Abcam (Cambridge, MA, United States). Antibodies for phosphorylated c-Jun N-terminal kinase (JNK; Thr183/Tyr185; Catalog No. 9255), JNK (Catalog No. 9252), Cx43 (Catalog No. 3512); phosphorylated Cx43 (Ser368; Catalog No. 3511) phosphorylated AMPK (Thr172; Catalog No. 2531), AMPK (Catalog No. 2532) were purchased from Cell Signaling Technology (Beverly, MA, United States). Antibody for β-actin (Catalog No. A1978) was purchased from Sigma (St. Louis, MO, United States). Fetal bovine serum (FBS) was purchased from Gibco, and other cell culture media and supplements were purchased from KenGEN (KenGEN BioTECH, China). All other reagents were purchased from Sigma-Aldrich (St. Louis, MO, United States).

### Animals and Treatment

Adult male ICR mice (18–22 g) at 8 weeks of age were provided by the Experimental Animal Center at Nanjing Medical University, Nanjing, China. Animals had free access to food and water and were housed in groups of five to six per cage under pathogen-free conditions with soft bedding under controlled temperature (22 ± 2°C) and a 12-h light/dark cycle (lights on at 8:00 a.m.) All procedures were conducted in accordance with the guidelines and regulations of the National Institutes of Health (NIH) and were approved by the Ethics Committee of Nanjing Medical University (No. IACUC-1908026).

Sixty mice were randomly divided into six groups (*n* = 10 in each group): Control group; Doxorubicin (Dox) group; Doxorubicin + Bilirubin (7.5, 15 and 30 mg/kg) group; Bilirubin (30 mg/kg) group. Doxorubicin was dissolved in sterile saline (0.9% NaCl); bilirubin was dissolved in DMSO solution and dilute to appropriate concentration before using. Acute cardiotoxicity was induced by a single dose of doxorubicin (20 mg/kg) *via* intraperitoneal (i.p.) injection in mice. Various concentrations of bilirubin (7.5, 15, and 30 mg/kg) were i.p. injection into mice 1 day before doxorubicin treatment, and then continuously injection for 7 or 12 days every 24 h. The mortality rate and body weight of the mice were recorded every day.

### Cell Cultures

H9C2 cells were purchased from the American Type Culture Collection and maintained in Dulbecco’s modified Eagle’s Medium (DMEM; KenGEN Bio TECH, China) supplemented with 10% (v/v) FBS (Gibco), penicillin (100 U/ml), and streptomycin (100 U/ml). All cells were kept in a humidified chamber with 5% CO_2_ at 37°C. For further experiments, H9C2 cells were seeded in 6-well plate at a density of 1 × 10^5^ cells/well. After 24 h, cells were treated with bilirubin (0.1 μM), AMPK agonists AICAR (20 μM) and metformin (1 mM) for 6 h, respectively, and then treated with doxorubicin 1 μM for 24 h. Cells were subsequently harvested and analyzed by immunoblot assay.

### Cardiomyocyte Damage Assessment

Cardiomyocyte integrity was assessed by determining the serum level of cardiomyocytes marker enzyme leakage. Serum samples were extracted from each animal and stored at −80°C for further analysis. The following cardiac injury markers were identified using commercial kits (R&D Systems, United States) and detected according to the manufacturer’s instructions: aspartate aminotransferase (AST); lactate dehydrogenase (LDH); creatine kinase MB (CK-MB); α-hydroxybutyrate dehydrogenase (α-HBDH). Cardiac troponin T (cTnT) Kits were purchased from Roche (China). Concentrations were calculated by referring to a standard curve, according to the manufacturer’s instructions.

### Electrocardiogram

Essentially, mice were anesthetized by 1% sodium amobarbital before undergoing ECG for heart rate and QT interval analysis. Electrodes were placed under the skin right hind limb, right front limb and left hind limb. The results were recorded using the MP150 ECG module (BIOPAC, United States). The duration of each recording was at least 5 min at 50 mm/s with a voltage of 1 mV/cm.

### Echocardiography Measurements

Cardiac function was evaluated on the 7th days after doxorubicin injection using transthoracic echocardiography measurements obtained with a Vevo2100 High Resolution Ultrasound System in real time (Visual Sonics Vevo 2100, Canada) and an M-mode ultrasound scanning transducer. Mice were anesthetized with 2% isoflurane mixed with 0.5 L/min 100% O_2_ and placed them on the heating pad in a supine position. M-mode imaging was used to obtain stable images of the parasternal long axis view. The left ventricular ejection fraction (LVEF) was calculated. All data were analyzed off-line at the end of the study with software resident on the ultrasound system and measured by an investigator who was blinded to the experimental groups.

### Masson Staining

On the 7th days after doxorubicin administration, mice were sacrificed, separated the heart and rinsed it with phosphate buffer. And then placed it in a 4% paraformaldehyde solution for 48 h, and then embedded it with paraffin. Each specimen was cut into slices of about 5 μm thickness and subjected to Masson staining. After the staining was completed, the sheets were preserved, and the myocardial fibrosis range was observed by an optical microscope (Olympus, Japan).

### Flow Cytometry

The FITC Annexin V Apoptosis Detection Kit I (BD Biosciences, CA, United States) was used to detect apoptotic cells according to the manufacturer’s instructions. Briefly, H9C2 cells were seeded in 6-well plates at a density of 1 × 10^6^ cells/well. After 24 h, bilirubin (0.1 μM) was added into the cells, then after 6 h, the cells were treated with or without doxorubicin (1 μM) for 24 h, collected the cells and washed with cold PBS and then resuspend cells in 1 × Binding Buffer, and then transfer 100 μl of the solution (1 × 10^5^ cells) to a 2 ml culture tube, and add 5 μl of FITC Annexin V and 5 μl PI, and gently vortex the cells and incubate for 15 min at 25°C in the dark room, and finally add 400 μl of Binding Buffer to each tube, and then analyze it by flow cytometry.

### Real-Time Cell Analyzer

Doxorubicin-induced cardiotoxicity was determined by using the xCELLigence platform (real-time cell analyzer, RTCA) as previously studies (Scrace et al., 2013). Briefly, after setting up the program, 50 μl of the culture medium per well was dropped into the E-plate to plot the baseline, followed by seeding 1 × 10^4^ cells in each well. The cells were placed at room temperature for 30 min to attach to the E-plate before subsequent detection. The xCELLigence RTCA system was used to monitor cell kinetics across microelectronic sensors integrated into the bottom of the plate.

### Western Blotting

Samples (cells or heart tissue) were collected and washed with PBS before being lysed in radio immunoprecipitation assay (RIPA) lysis buffer. The protein concentrations were determined by BCA Protein Assay (Thermo Fisher, Waltham, MA, United States) and 40–80 μg of proteins were loaded and separated by SDS-PAGE and electrophoretically transferred onto polyvinylidene fluoride membranes (Millipore Corp., Bedford, MA, United States). The membranes were blocked with 5% bovine serum albumin for 2 h at room temperature, probed with antibodies overnight at 4°C with the primary antibodies and then incubated with HRP-coupled secondary antibodies. The primary antibodies used included p-AMPK (Thr172) (1:1000), AMPK (1:1000), Axl (1:1000), SOCS3 (1:1000), p-JNK (Thr183/Tyr185) (1:1000), JNK (1:1000), p-Cx43 (1:1000), total Cx43 (1:1000). For loading control, the blots were probed with antibody for β-actin (1:1000). The filters were then developed by enhanced chemiluminescence reagents (PerkinElmer, Waltham, MA, United States) with secondary antibodies (Chemicon, Billerica, MA, United States). Data were acquired with the Molecular Imager (Gel DocTM XR, 170-8170) and analyzed with Quantity One-4.6.5 (Bio-Rad Laboratories, Berkeley, CA, United States).

### RNA Interference

Axl siRNA (m, sc-29770) and control siRNA (sc-37007) were purchased from Santa Cruz Biotechnology (Santa Cruz, CA, United States). 3.3 nmol siRNA was dissolved in 330 μl RNase-free water. Control siRNA was used as a negative control. For the transfection of siRNA, H9C2 cells were cultured in six-well plates with antibiotic-free medium the day before transfection. The transfection was conducted when cells reached 60%∼80% confluence using Lipofectamine 2000 (Invitrogen, United States) and serum-free medium according to the manufacturer’s instructions. After 6 h, the transfection medium was replaced with the culture medium containing 10% FBS and then incubated at 37°C in 5% CO_2_.

### Statistical Analyses

GraphPad Prism 7 software (GraphPad Software, San Diego, CA, United States) was used to conduct all the statistical analyses. Kaplan Meier Survival analysis was completed using the Log rank (Mantel-Cox) test. Data were statistically evaluated by one-way or two-way analysis of variance (ANOVA) followed by Bonferroni post hoc tests. Results were represented as mean ± SEM of three independent experiments. *p* < 0.05 was deemed to be statistically significant.

## Results

### Doxorubicin Significantly Induces Abnormal Heart Rhythmb in Mice

**FIGURE 1:**
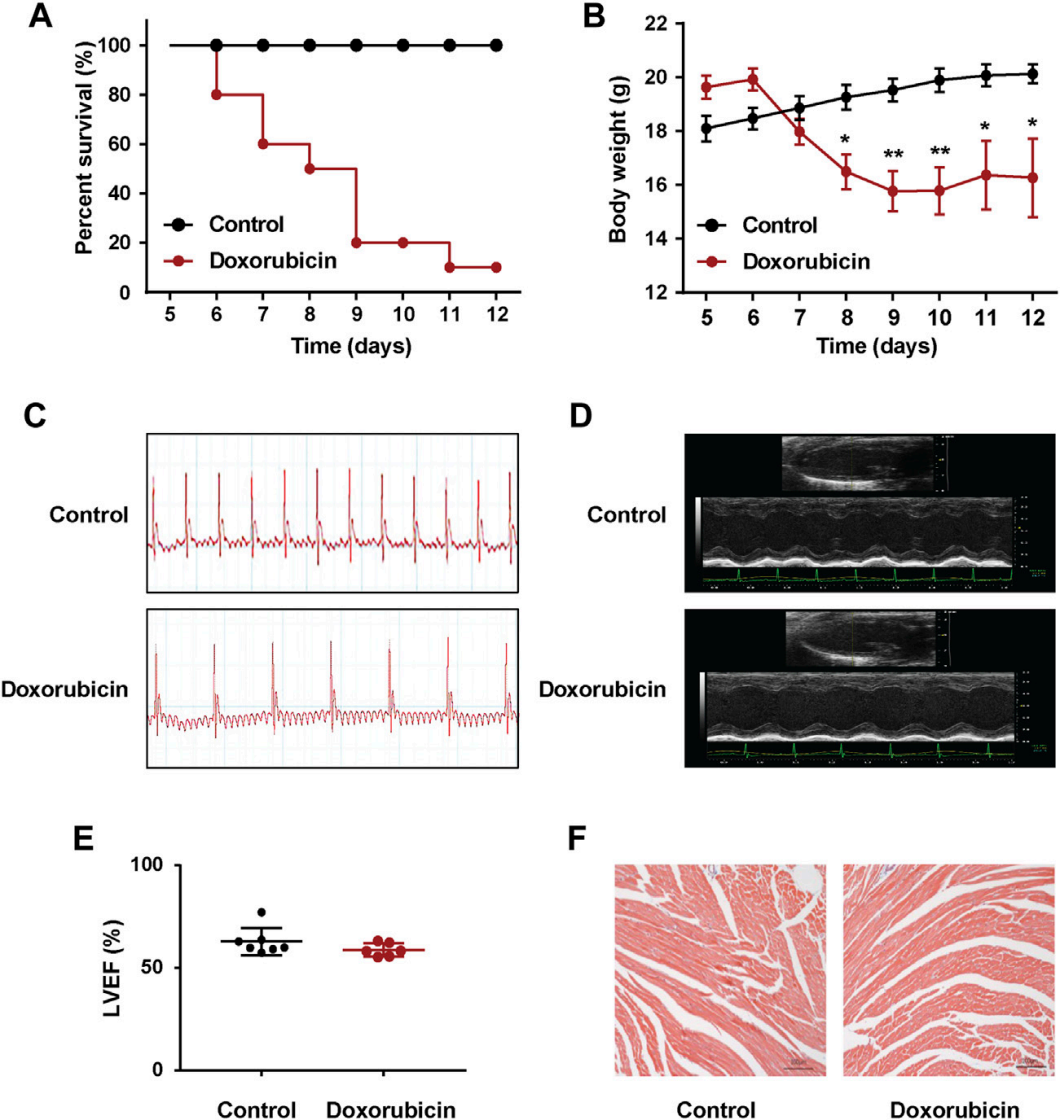
Doxorubicin significantly induces abnormal heart rhythm in mice. Mice received a single administration of doxorubicin (20 mg/kg, i.p.) or vehicle (0.9% NaCl, i.p.). **(A)** Kaplan Meier survival analysis showed that the survival rate of doxorubicin-treatment mice. The Log rank (Mantel-Cox) test was used to determine significance (*n* = 10 of each group). **(B)** Body weights was measured every day for consecutive 12 days. **(C)** Representative images of electrocardiogram (ECG), and **(D)** echocardiography were examined on Day 7 after doxorubicin administration. **(E)** Quantitative data of the LVEF values in mice. **(F)** Masson staining of myocardial sections in mice. Magnification: ×100. Scale bar: 100 μm. (*n* = 4 of each group). Significant difference was revealed following one-way ANOVA (*p < 0.05, **p < 0.01 vs. Control group; Bonferroni post hoc tests).

As shown in Figures 1A,B, the mortality rate of the mice reached 90% on Day 12 after doxorubicin administration, and the weight was 20% lower than Day 0. Considering the excessively high mortality rate of mice on Day 12, we repeated the experiment and measured the II lead ECG of mice on Day 7. Compared with the Control group, doxorubicin significantly induced arrhythmia in mice (Figure 1C). Moreover, utilizing echocardiography examination revealed that the LVEF values of doxorubicin mice did not change very obvious on Day 7, only with a slight decrease but have no statistical difference (Figures 1D,E), and there were also no obvious changes with Masson staining in heart (Figure 1F). These data suggested that the death of doxorubicin mice may be caused by severe arrhythmia.

### Bilirubin Protects Against Doxorubicin-Induced Cardiotoxicity

**FIGURE 2:**
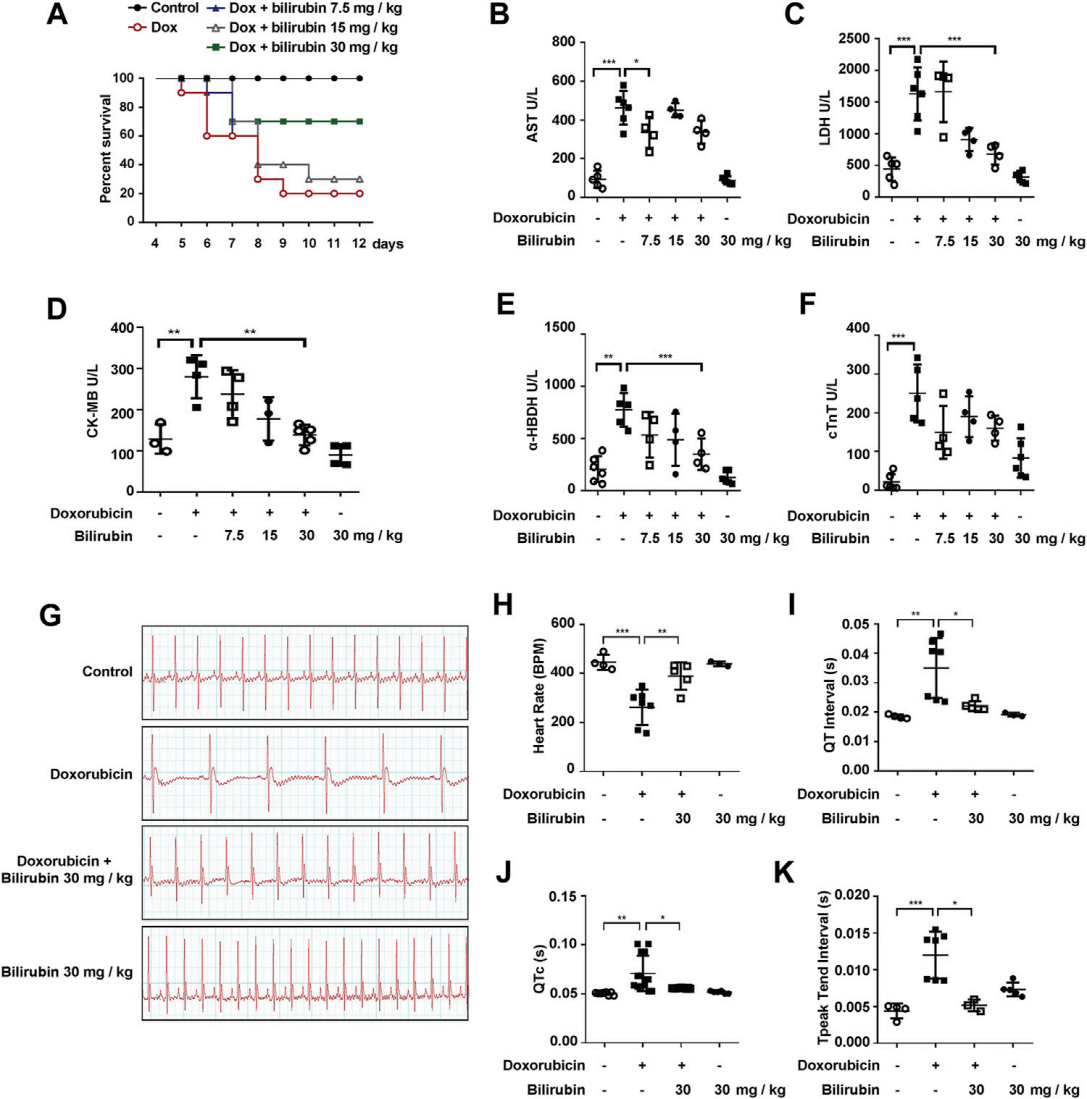
Bilirubin protects against doxorubicin-induced cardiotoxicity. **(A)** Survival rate was measured every day for consecutive 12 days (*n* = 10 of each group). The level of myocardial enzyme including AST **(B)**, LDH **(C)**, CK-MB **(D)**, α-HBDH **(E)** and cTnT **(F)** were measured by the Elisa kits. **(G)** Representative images of ECG and the quantitative data of heart rate **(H)**, QT interval **(I)**, QTc **(J)** and T-peak Tend Interval **(K)** showed the cardiac conduction function in mice. The plasma samples (*n* ≥ 4 of each group) were collected after the last bilirubin treatment on Day 7. Significant difference was revealed following one-way or two-way ANOVA (*p < 0.05, **p < 0.01, ***p < 0.001 vs. Control group; ^#^ *p* < 0.05, ^##^ *p* < 0.01, ^###^ *p* < 0.001 vs. Doxorubicin-treated group; Bonferroni post hoc tests).

We further investigated the effects of bilirubin on doxorubicin-induced cardiotoxicity and cardiac conduction abnormalities. As the results shown in Figure 2, pretreatment with bilirubin (30 mg/kg, i.p*.*) significantly increased the survival rate compared to the Doxorubicin-treated group (Figure 2A). In addition, the cardioprotective effect of bilirubin was evaluated by measuring the level of myocardial enzyme (AST, LDH, CK-MB, α-HBDH, and cTnT). As shown in Figures 2B–F, doxorubicin increased the level of AST, LDH, CK-MB, and α-HBDH compared to the Control group, while pretreatment with bilirubin markedly reduced the level of these myocardial enzyme. Moreover, we also performed ECG to detect whether pretreatment with bilirubin would ameliorate doxorubicin-induced conduction abnormalities. As shown in Figures 2G–K, doxorubicin (20 mg/kg, i.p*.*) caused a reduction in cardiac function, such as reduced the heart rate, prolonged the QT, QTc, and T-peak Tend Interval (Figures 2G–K). Pretreatment with bilirubin effectively reversed the doxorubicin-induced bradycardia and partly compromise the doxorubicin-induced extension of QT, QTc, and T-peak Tend interval (Figures 2G–K).

### Bilirubin Reduces Doxorubicin-Induced JNK-Cx43 Gap Junctions’ Dysfunction

**FIGURE 3:**
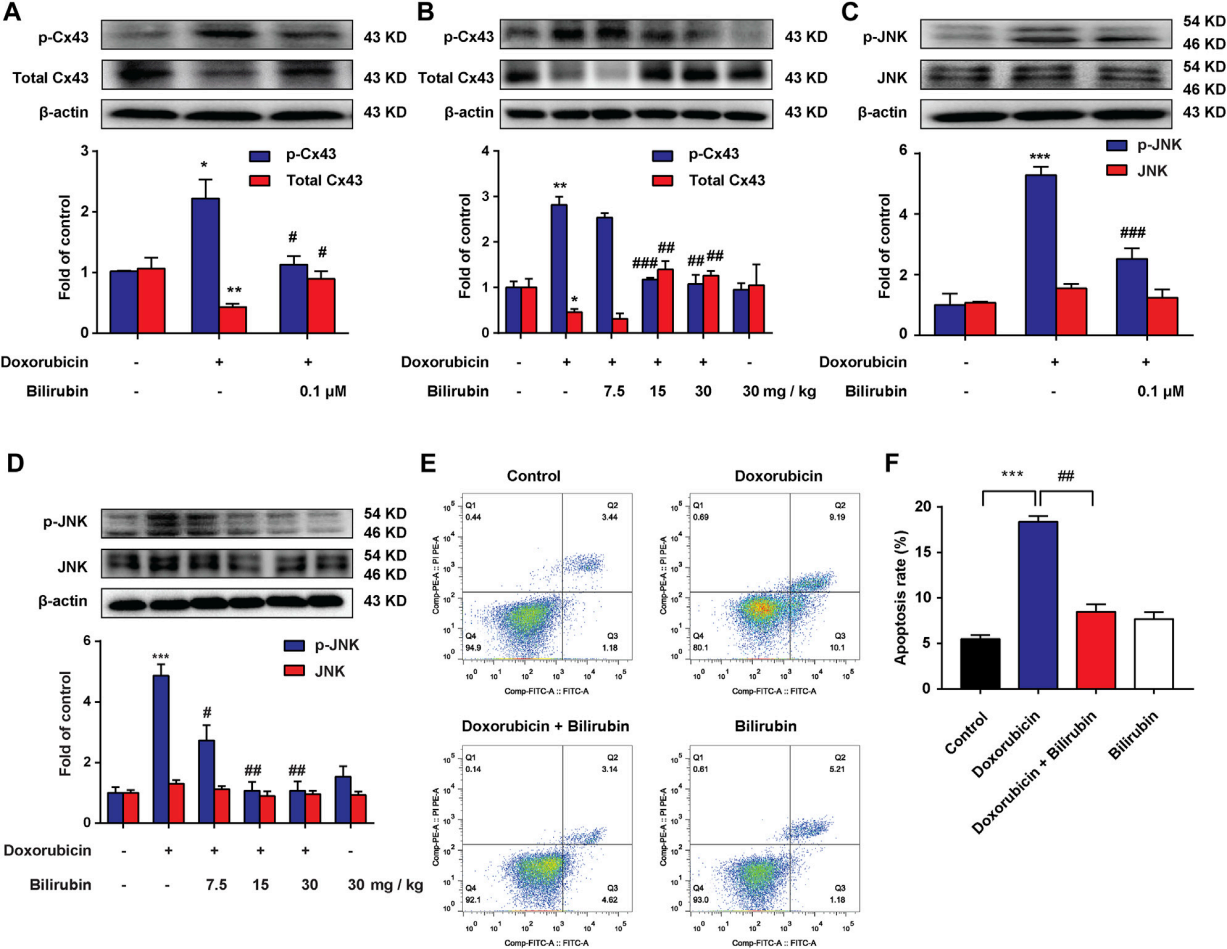
Bilirubin reduces doxorubicin-induced JNK-Cx43 gap junctions’ dysfunction. **(A,C)** Representative Western blot images showed the levels of p-Cx43, total Cx43, p-JNK and JNK in H9C2 cells *in vitro*. **(B,D)** Representative Western blot images showed the levels of p-Cx43, total Cx43, p-JNK and JNK in heart tissues from doxorubicin-mice given different dose of bilirubin (7.5, 15, and 30 mg/kg, i.p.) for 7 days. The western blot samples were collected and analyzed (*n* = 4 of each group). **(E)** Apoptosis was assessed by flow cytometric analysis in H9C2 cells. **(F)** Quantitative data of the apoptosis ratio analyzed by flow cytometry. H9C2 cells were treated with bilirubin (0.1 μM) for 6 h before doxorubicin (1 μM) treatment, and the samples were collected 24 h after doxorubicin treatment (*n* = 4 of each group). Significant difference was revealed following one-way ANOVA (*p < 0.05, **p < 0.01, ***p < 0.001 vs. Control group; ^#^ *p* < 0.05, ^##^ *p* < 0.01, ^###^ *p* < 0.001 vs. Doxorubicin-treated group; Bonferroni post hoc tests).

Previous studies have reported that doxorubicin could lead to conduction abnormalities on the heart in rats (Aygun and Gul, 2019). Gap junction plays an important role in cardiac conduction. Additionally, lessening the expression of total Cx43 protein would lead to reduce intercellular coupling and the conduction velocity (Petrich et al., 2004; Fontes et al., 2012). In this study, we investigated the effect of bilirubin on doxorubicin-induced Cx43 gap junction. Compared with the Control group, doxorubicin markedly decreased total Cx43 expression and increased Cx43 phosphorylation in H9C2 cells (Figure 3A) or in mice heart (Figure 3B). Bilirubin (15 and 30 mg/kg, i.p*.*) increased total Cx43 expression and decreased Cx43 phosphorylation in H9C2 cells or in mice heart. Doxorubicin also induced the phosphorylation of JNK in H9C2 cells *in vitro* (Figure 3C) or in the heart *in vivo* (Figure 3D), and these effects can also be reversed by bilirubin. Additionally, bilirubin could reduce apoptosis of H9C2 cells induced by doxorubicin *in vitro* (Figures 3E,F). These data suggested that bilirubin could improve gap junctions’ function and protect against cardiomyocyte apoptosis induced by doxorubicin.

### Bilirubin Increases AMPK Phosphorylation and SOCS3 Expression

**FIGURE 4:**
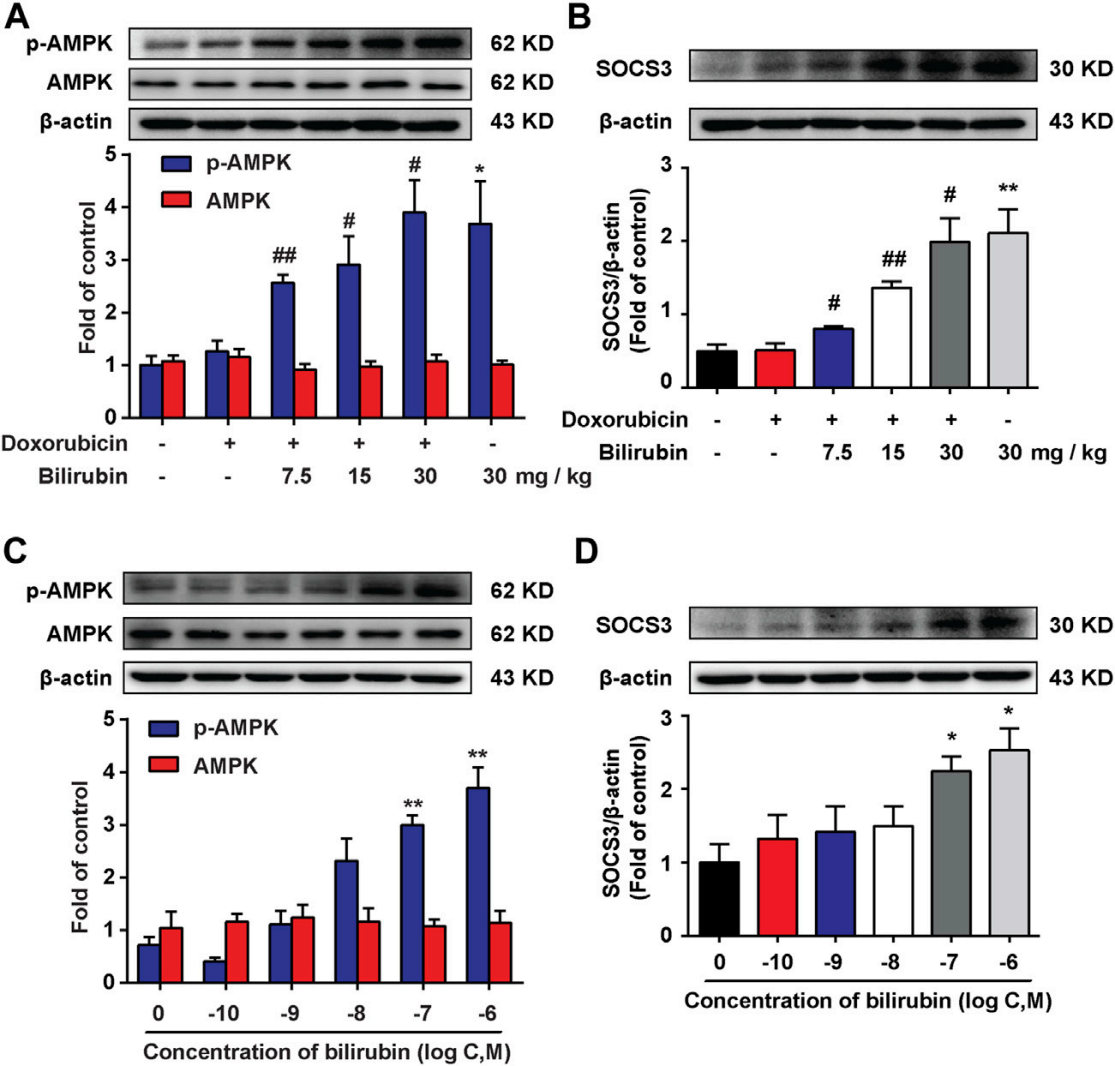
Bilirubin increases AMPK phosphorylation and SOCS3 expression. **(A,B)** Representative western blot bands showed the levels of p-AMPK, AMPK, and SOCS3 in heart tissues from mice given different dosages of bilirubin (7.5, 15, and 30 mg/kg, i.p.) for 7 days, (*n* = 4 of each group). **(C,D)** Representative Western blot bands shown the levels of p-AMPK, AMPK, and SOCS3 in H9C2 cells cultured with different concentrations of bilirubin for 6 h. Significant difference was revealed following one-way ANOVA (*p < 0.05, **p < 0.01 vs. Control group; ^#^ *p* < 0.05, ^##^ *p* < 0.01 vs. Doxorubicin-treated group; Bonferroni post hoc tests).

AMPK, a metabolic sensitive serine/threonine protein kinase, which can be activated by many stimulators, such as hypoxia and cell stress (Inoki et al., 2012). Additionally, our previous study found that AMPK activation could induce the expression of SOCS3, an endogenous inflammatory suppressor (Zhang et al., 2017). In the present study, we further investigate the effects of bilirubin on AMPK and SOCS3. As shown in Figures 4A,B, compared with the doxorubicin group, bilirubin (7.5, 15, and 30 mg/kg, i.p*.*) significantly increase AMPK phosphorylation and SOCS3 expression in the mice heart *in vivo*. *In vitro*, H9C2 cells were co-cultured with or without bilirubin (10^−10^–10^−6^ M) for 6 h. Data showed that bilirubin (10^−7^ M) sufficiently induced the phosphorylation of AMPK and SOCS3 expression in H9C2 cells *in vitro* (Figures 4C,D). These data suggested that bilirubin may activate AMPK/SOCS3 axis to inhibit inflammatory response.

### Bilirubin Protects Against Doxorubicin-Induced Cardiotoxicity in an AMPK-SOCS3 Dependent Manner in H9C2 Cells

**FIGURE 5:**
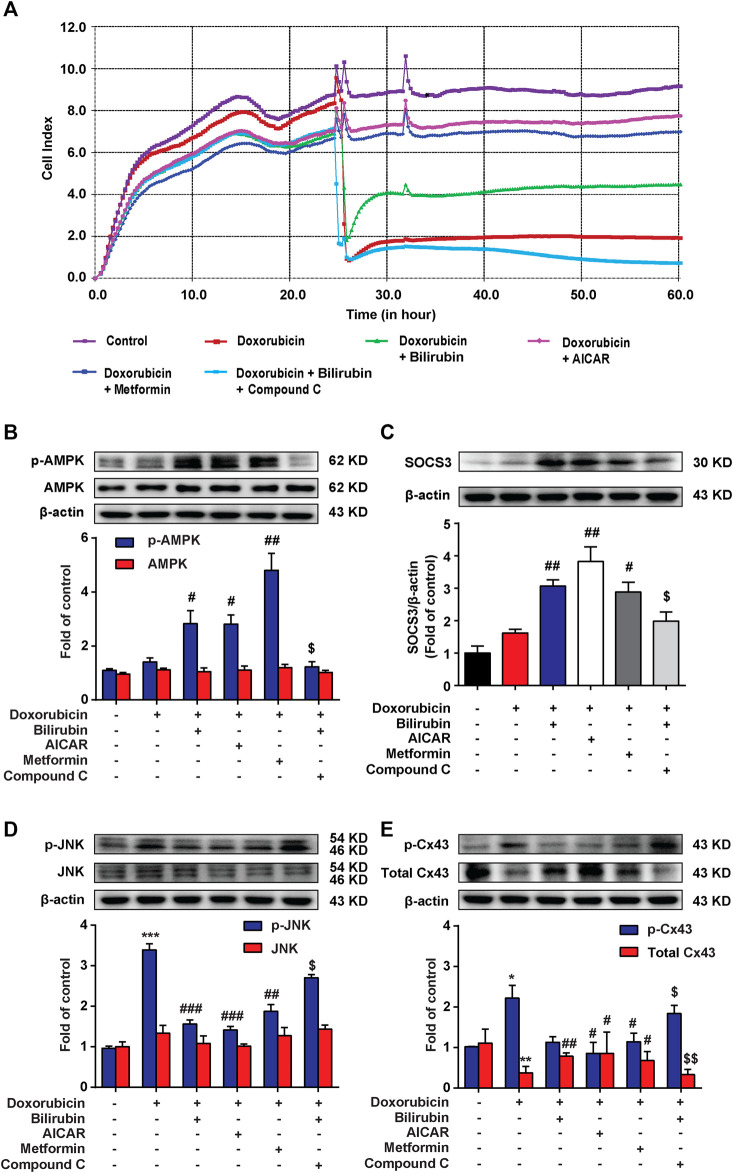
Bilirubin protects against Doxorubicin-induced cardiotoxicity in an AMPK-SOCS3 dependent manner in H9C2 cells. **(A)** RTCA (xCELLigence platform) was used to record the cell growth curves in H9C2 cells. **(B–E)** Representative western blot bands showed the levels of p-AMPK, AMPK, SOCS3, p-JNK, JNK, p-Cx43, and total Cx43 in H9C2 cells *in vitro*. H9C2 cells were pretreated with Compound C (2 μM) for half an hour before bilirubin treatment, and then cells were cultured with bilirubin (0.1 μM), AICAR (20 μM) and metformin (1 mM) for 6 h, followed by doxorubicin treatment (1 μM) for another 24 h. The western blot samples were collected and analyzed (*n* = 4 of each group). Significant difference was revealed following one-way ANOVA (*p < 0.05, ***p < 0.001 vs. Control group; ^#^ *p* < 0.05, ^##^ *p* < 0.01, ^###^ *p* < 0.001 vs. Doxorubicin-treated group; ^$^ *p* < 0.05 vs. Doxorubicin and bilirubin-treated group; Bonferroni post hoc tests).

We further investigated whether the cardioprotective effect of bilirubin was dependent on AMPK-SOCS3 signaling *in vitro*. The RTCA experiment was used to measure doxorubicin-induced cardiotoxicity in H9C2 cells. As shown in Figure 5A, pretreatment with bilirubin (10^−7^ M) for 6 h significantly alleviated doxorubicin-induced cardiotoxicity, which was abolished by AMPK inhibitor compound C. In addition, we used two AMPK agonists (AICAR and metformin) on H9C2 cells for verification. As shown in Figure 5A, AICAR and metformin all mimicked the protective effect of bilirubin, significantly alleviating doxorubicin-induced cardiotoxicity. Meanwhile, we measured the effects of bilirubin on the expression of p-AMPK, SOCS3, p-JNK, and p-Cx43. As shown in Figures 5B–E, compared with the doxorubicin group, bilirubin and AMPK agonists (AICAR and metformin) increased AMPK phosphorylation and induced SOCS3 expression, and decreased the phosphorylation of JNK and Cx43, whereas these effects could be abolished by AMPK inhibitor Compound C. These data suggested that bilirubin could mimic AMPK activator to induce SOCS3 expression and inhibit JNK-Cx43 axis to improve gap junctions’ function.

### Bilirubin Induces SOCS3 Expression *via* Activating Axl Receptor in H9C2 Cells

**FIGURE 6:**
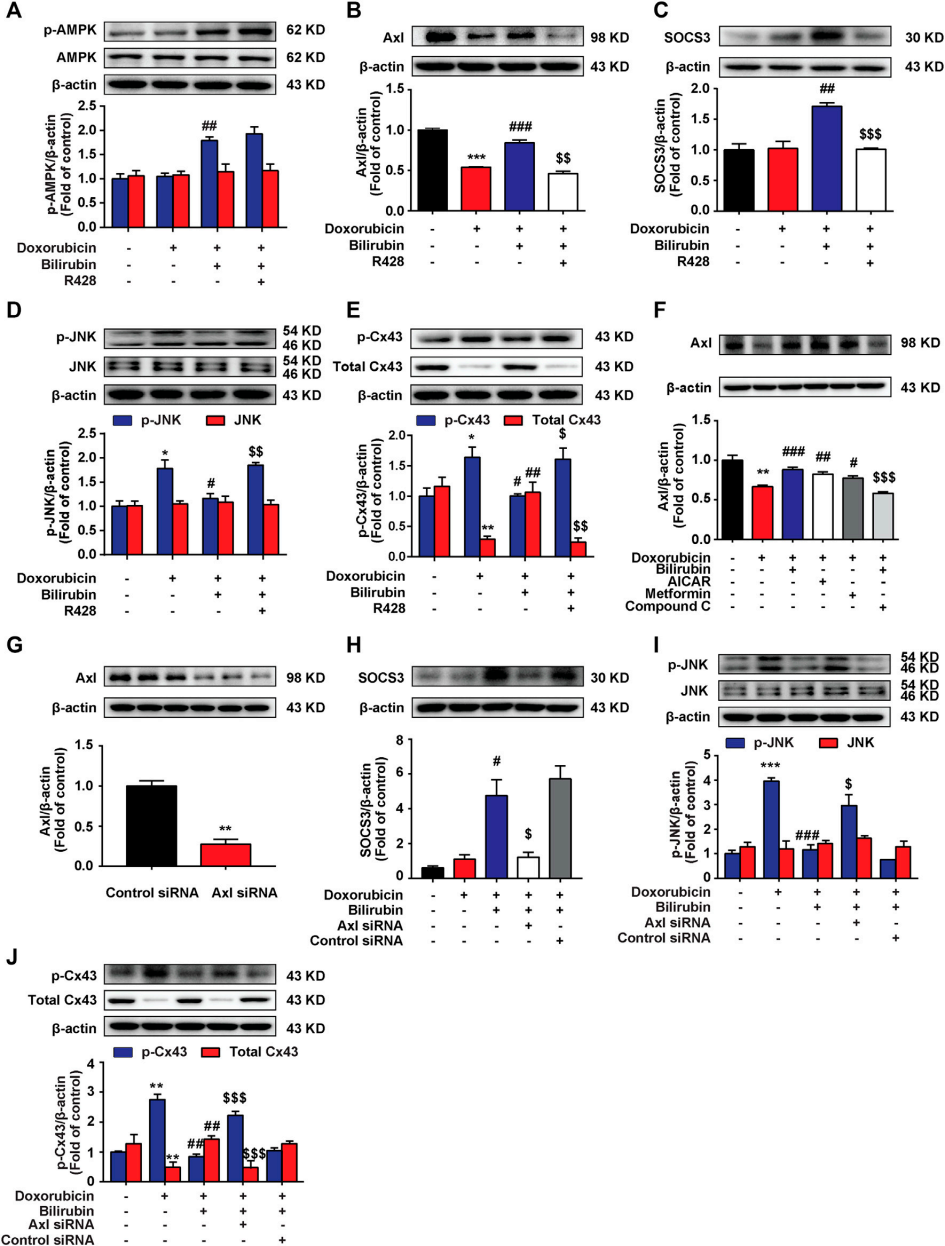
Bilirubin induces SOCS3 expression *via* activating Axl receptor in H9C2 cells. **(A–E)** Representative western blot bands showed the levels of p-AMPK, AMPK, Axl, SOCS3, p-JNK, JNK, p-Cx43, and total Cx43 in H9C2 cells *in vitro*. H9C2 cells were pretreated with R428 (1 μM) for half an hour before bilirubin treatment, and then cells were cultured with bilirubin (0.1 μM) for 6 h, followed by doxorubicin treatment (1 μM) for another 24 h. The western blot samples were collected and analyzed (*n* = 4 of each group). **(F)** Representative western blot bands showed the levels of Axl in H9C2 cells. **(G)** The efficiency of Axl knockdown was assessed by western blot assay. **(H–J)** Representative western blot bands showed the levels of SOCS3, p-JNK, JNK, p-Cx43, and total Cx43 in H9C2 cells. H9C2 cells were transfected with 100 pM Axl siRNA or control siRNA for 24 h, and then subjected to bilirubin (0.1 μM) for 6 h, followed by exposure to doxorubicin (1 μM) for another 24 h. The western blot samples were collected and analyzed (*n* ≥ 3 each group). Significant difference was revealed following one-way ANOVA (*p < 0.05, **p < 0.01, ***p < 0.001 vs. Control group; ^#^ *p* < 0.05, ^##^ *p* < 0.01, ^###^ *p* < 0.001 vs. Doxorubicin-treated group; ^$^ *p* < 0.05, ^$$^ *p* < 0.01, ^$$$^ *p* < 0.001 vs. Doxorubicin and bilirubin-treated group; Bonferroni post hoc tests).

Our previous study indicated that AMPK activation could induce the upregulation of SOCS3 in BV-2 cells (Zhang et al., 2017). Here, we also found that bilirubin could induce SOCS3 expression by activating AMPK in H9C2 cells, and further explored the mechanism. It is demonstrated that activated TAM receptors can significantly upregulate the expression of SOCS3 (Rothlin et al., 2007). TAM receptors have three members include Tyro3, Axl, and Mer receptor. Considering the high level of Axl receptor in the heart (Batlle et al., 2014), we explored whether Axl receptor participated in AMPK inducing SOCS3 expression and the cardioprotective effect of bilirubin. As shown in Figures 6A–C, bilirubin significantly increased the expression of p-AMPK, Axl, and SOCS3, and decreased the phosphorylation level of JNK and Cx43 *in vitro* (Figures 6D,E). Interestingly, Axl inhibitor R428 abolished the effects of bilirubin on these target proteins except p-AMPK, suggesting that Axl is not the upstream target of AMPK. We further used bilirubin and AMPK activators to determine the upstream and downstream relationship between AMPK and Axl. As shown in Figure 6F, compared with the doxorubicin group, bilirubin significantly increased the expression of Axl, which was abolished by the AMPK inhibitor Compound C. AMPK activators (AICAR and metformin) also mimic the effect of bilirubin to increase the expression of Axl. Axl siRNA was used to further verify the effects of bilirubin on H9C2 cells *in vitro*. As shown in Figures 6G,H, compared with the bilirubin group, knockdown of Axl using siRNA markedly decreased the expression of SOCS3 induced by bilirubin (Figure 6H), and abolished the inhibition effect of bilirubin on the phosphorylation of JNK and Cx43. These data suggested that bilirubin improved the Cx43-gap junctions’ function *via* activating AMPK/Axl signaling pathway.

## Discussion

In this study, the major findings were as follows: 1) doxorubicin could induce abnormal heart rhythm in mice; 2) bilirubin alleviates JNK-Cx43 gap junctions’ dysfunction; 3) bilirubin activates AMPK-SOCS3 axis to inhibit Cx43 phosphorylation and protect H9C2 cells against doxorubicin toxic injury; 4) Axl is involved in bilirubin-induced SOCS3 expression.

Currently, many clinical guidelines recommend a maximum dose of doxorubicin was 450 mg/m^2^, which aims to reduce the cardiotoxicity by limiting the cumulative dose of doxorubicin. Unfortunately, it cannot reduce late-onset chronic progressive cardiotoxicity (Doyle et al., 2005).

Previous evidences have indicated an association between liver dysfunction and heart failure in humans (Correale et al., 2018), but the underlying mechanisms is still not clear. It is shown that the extent of myocardial damage in rats with partial hepatectomy was further increased after doxorubicin administration, while supernatant collected from doxorubicin-treated hepatocytes could alleviate the cardiotoxicity in H9C2 cells *in vitro* (Zhang et al., 2014). Bilirubin is the end product of heme catabolism in mammals. High bilirubin levels are generally considered to be a marker of liver damage (Giannini et al., 2005). Interestingly, recent study have demonstrated that bilirubin may have a certain cytoprotective effects (Sedlak et al., 2009). Bilirubin exhibits potent anti-oxidant properties which can prevent the oxidative damage triggered by a wide range of oxidant-related stimuli (Tomaro et al., 2002). Schwertner and Vitek’ studies have observed that the incidence of heart disease in patients with Gilbert’s syndrome (individuals with higher bilirubin levels) was lower than general population (Vitek et al., 2002; Schwertner and Vitek, 2008; Vitek, 2016). Low total bilirubin level has been shown to be a risk factor for cardiovascular diseases (Schwertner et al., 1994; Shcherbinina, 2007). Moreover, studies have shown that bilirubin may help to resist multidrug resistance in tumor (Rathinaraj et al., 2020). Thus, we hypothesized that bilirubin may have a cardioprotective effect in doxorubicin-induced cardiotoxicity.

In this study, we found that bilirubin could significantly attenuate doxorubicin-induced abnormal heart rhythmb, increase the survival rate and reduce the level of AST, LDH, CK-MB, and α-HBDH compared to the doxorubicin group (Figures 1, 2). Bilirubin also effectively reversed the doxorubicin-induced bradycardia, QT prolongation and arrhythmia in mice (Figures 2G–K). Besides, *in vitro* bilirubin could reduce doxorubicin-induced myocardial apoptosis *in vitro* (Figure 3E). Therefore, we speculate that bilirubin protects against doxorubicin-induced cardiotoxicity may by inhibiting myocardial apoptosis and ameliorating cardiac conduction abnormalities.

Previous studies have reported that Cx43 gap junction is closely related to cardiac conduction (Jongsma and Wilders, 2000; Fontes et al., 2012; Sorgen et al., 2018). Downregulation of Cx43 has been observed in both human and animal models of heart failure (Dupont et al., 2001; Kostin et al., 2003). It is reported that JNK plays an important role in regulating Cx43 gap junction (Petrich et al., 2004). JNK activation not only can lead to a loss of specificity of total Cx43 protein and gap junction, reduce conduction velocity (Petrich et al., 2004), but also increase the phosphorylation of Cx43 and reduced Cx43-mediated cell-to-cell communication (Jones and Lancaster, 2015; Jabr et al., 2016). Here, we found that doxorubicin could significantly increase the phosphorylation of JNK and Cx43, and reduce the expression of total Cx43 protein *in vitro* and *in vivo* (Figures 3A–D). Pretreatment of bilirubin could reverse these effects of doxorubicin (Figures 3A–D). These results indicated that bilirubin protect against doxorubicin-induced cardiotoxicity may by inhibiting p-JNK-Cx43 signaling pathway.

Our previous study demonstrated that preemptive inducing the body to produce an inflammation tolerance *via* inducing SOCS3 expression could inhibit neuroinflammation (Fan et al., 2018). SOCS3 is an important negative regulator, negatively regulating various cytokine signaling, such as IL-1R, TNF-R, and IL-6R signaling pathways (Croker et al., 2003; Kubo et al., 2003). Therefore, we speculated that bilirubin preconditioning may protect against doxorubicin-induced cardiotoxicity *via* inducing SOCS3 expression to form inflammation tolerance. We found that bilirubin significantly increased the expression of SOCS3 both *in vivo* and *in vitro* (Figures 4B,D). Moreover, our previous study has found that proper activated AMPK can increase the expression of SOCS3 and induced inflammatory tolerance in mice (Zhang et al., 2017). Here we found that bilirubin could also increase the phosphorylation of AMPK both *in vivo* and *in vitro* (Figures 4A,C). AMPK inhibition abolished the upregulation effect of bilirubin on p-AMPK and SOCS3 *in vitro*, and reverse the protective effect of bilirubin on doxorubicin-induced cardiotoxicity (Figures 5D,E). AMPK agonists (AICAR and metformin) could mimic the cardioprotective effect of bilirubin and promote the expression of SOCS3. Furthermore, bilirubin decreased the phosphorylation of JNK and Cx43 induced by doxorubicin, which was mimicked by AMPK activators and abolished by AMPK inhibition *in vitro* (Figures 5B,C). These findings suggested that bilirubin protected against doxorubicin-induced cardiotoxicity may by activating AMPK-SOCS3 axis to improve Cx43 gap junctions’ function.

**FIGURE 7:**
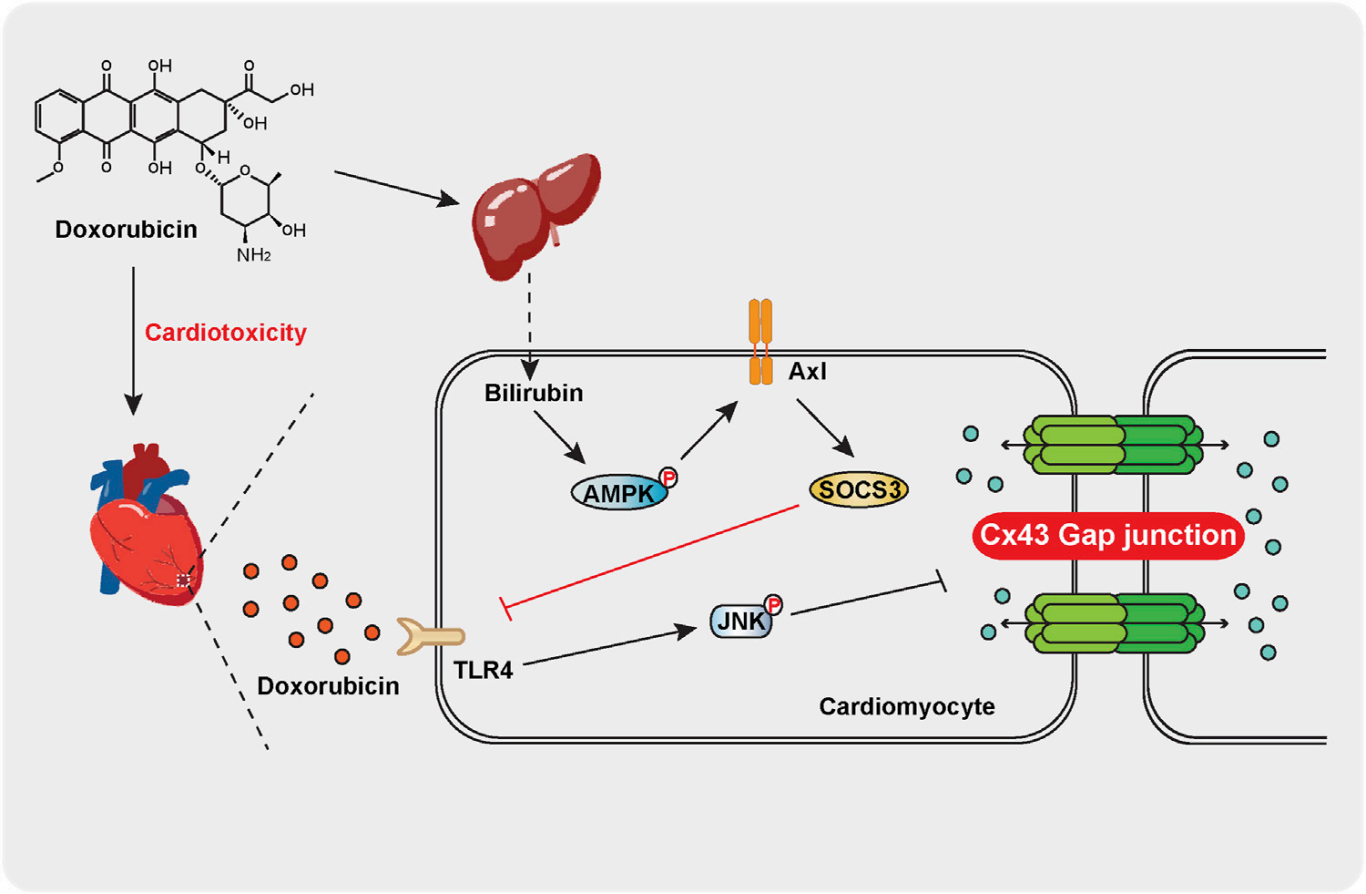
Schematic indicating that moderate increase bilirubin can inhibit doxorubicin-induced gap junction’ dysfunction and conduction abnormalities *via* regulating AMPK-Axl-SOCS3 signaling axis. Doxorubicin significantly increases the phosphorylation of JNK and Cx43 in mouse cardiomyocytes, leading to gap junction’ dysfunction and conduction abnormalities and causing cardiotoxicity. Bilirubin induces SOCS3 expression in an AMPK-Axl-dependent manner, inhibiting JNK-Cx43 signaling pathway to improve gap junction’ function and conduction abnormalities.

We further investigated the mechanism of bilirubin inducing the expression of SOCS3 in heart. Previous studies have found that activated TAM receptors can significantly upregulate the expression of SOCS3 (Rothlin et al., 2007). Moreover, activation of Gas6/Axl signaling pathway can alleviate cardiac fibrosis and apoptosis (Chen et al., 2019). Due to Axl receptor is highly expressed in the heart (Batlle et al., 2014), we speculated that bilirubin inducing the expression of SOCS3 may by activating the Axl receptor in heart. We found that bilirubin could significantly increase the expression of p-AMPK, Axl and SOCS3, and inhibit the phosphorylation of JNK and Cx43 induced by doxorubicin *in vitro* (Figure 6). AMPK inhibition suppressed the activation effect of bilirubin on Axl receptor (Figure 6F). Meanwhile, Axl receptor inhibitor (R428) could reverse the effects of bilirubin on Axl, SOCS3, p-JNK, and p-Cx43. Furthermore, knockdown Axl sufficiently abolished the expression of SOCS3, and repealed the inhibition of bilirubin on phosphorylated JNK and Cx43 *in vitro*. These data suggested that the cardioprotective effect of bilirubin may by activating AMPK/Axl/SOCS3 to decrease Cx43 phosphorylation and improve gap junctions’ function (Figure 7).

## Conclusion

We provided the experimental evidences that bilirubin could protect against doxorubicin-induced cardiotoxicity *via* activating AMPK-Axl-SOCS3 axis to improve JNK-Cx43 gap junction’ function and conduction abnormalities. The interaction between the liver and the heart is beneficial for protecting against doxorubicin-induced cardiotoxicity.

## Data Availability

The original contributions presented in the study are included in the article/Supplementary Material, further inquiries can be directed to the corresponding authors.
