# Supplementary material for: Bilirubin Improves Gap Junction to Alleviate Doxorubicin-Induced Cardiotoxicity by Regulating AMPK-Axl-SOCS3-Cx43 Axis
Source: Front Pharmacol. 2022 Apr 25;13:828890. doi: 10.3389/fphar.2022.828890 (PMC9082937; doi:10.3389/fphar.2022.828890)
Supplement: Supplementary file 1 [file DataSheet1.ZIP › Original pictures for figures/Original pictures for figures.pdf]

Figure 1C

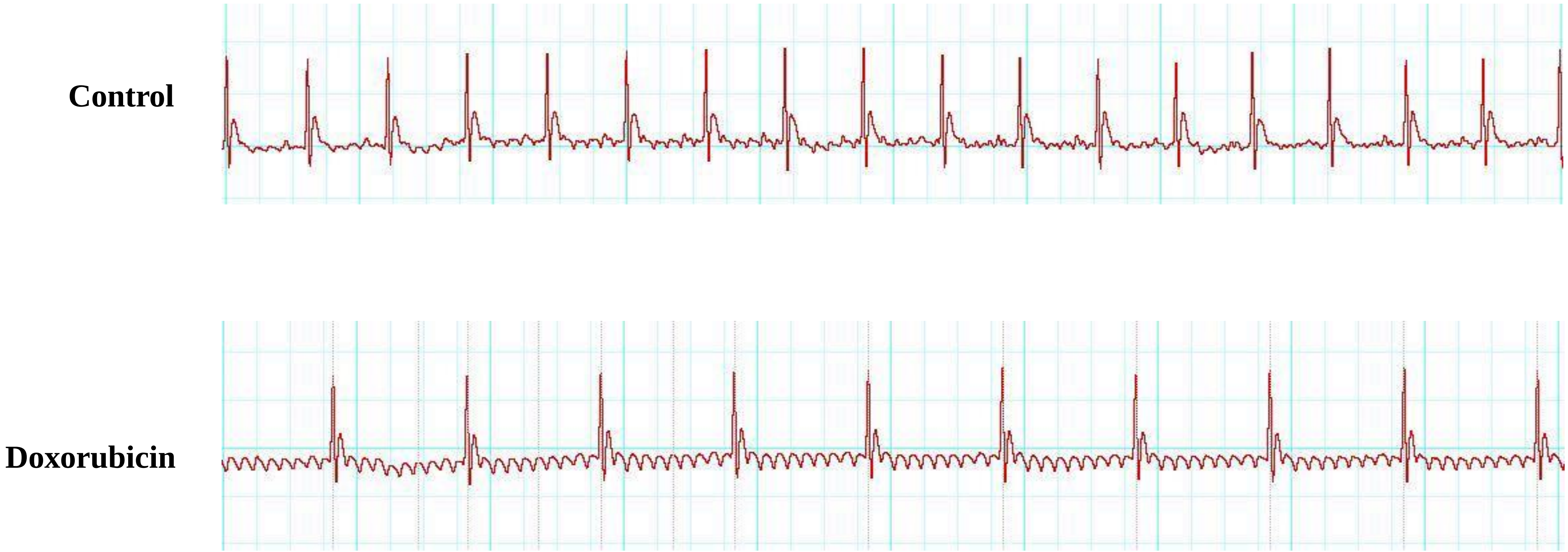

Figure 1D

Control

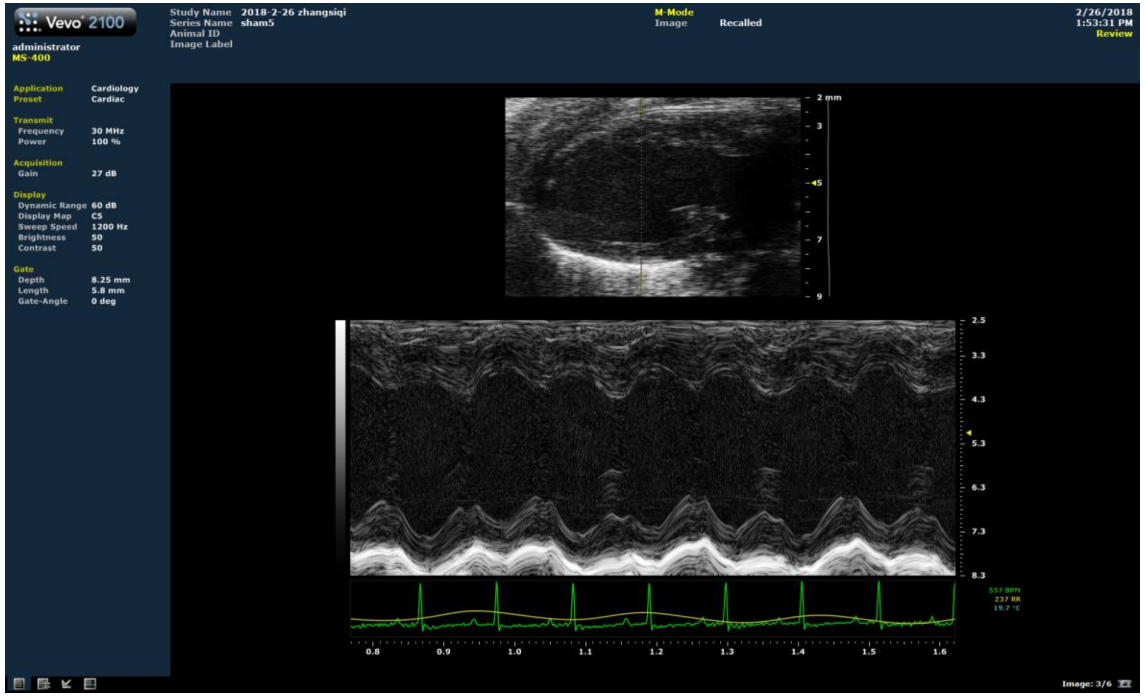

Doxorubicin

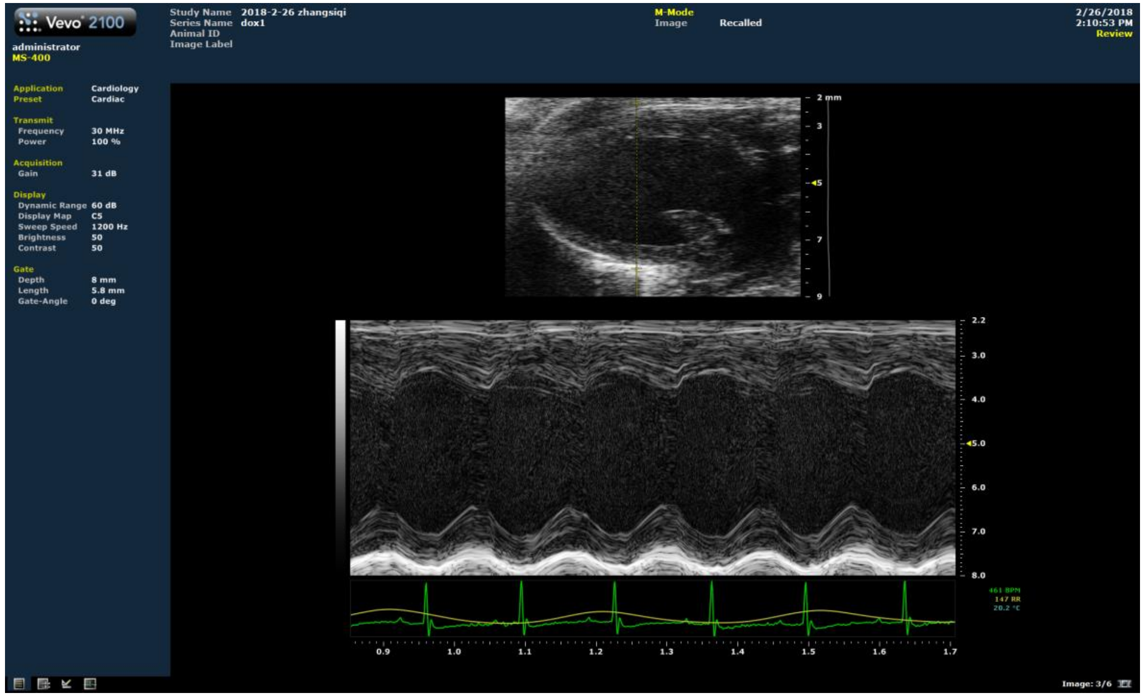

Figure 1F

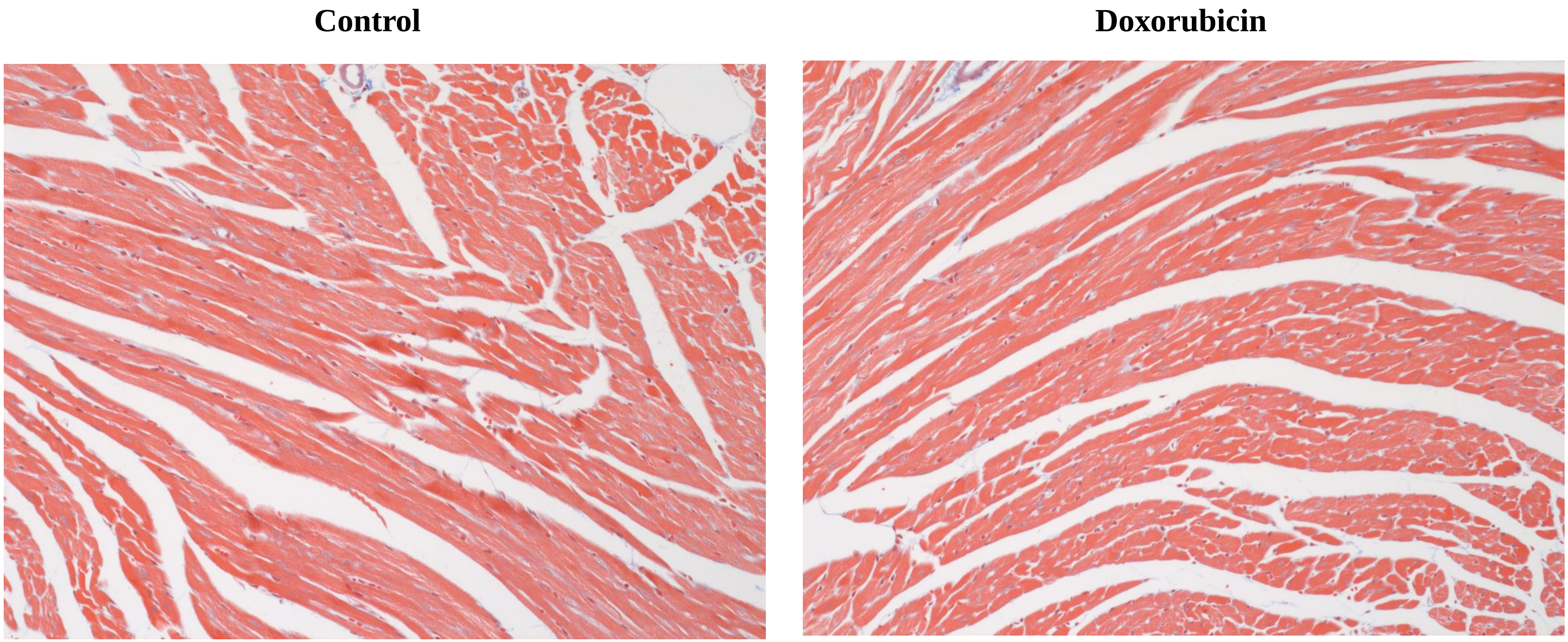

Figure 2G

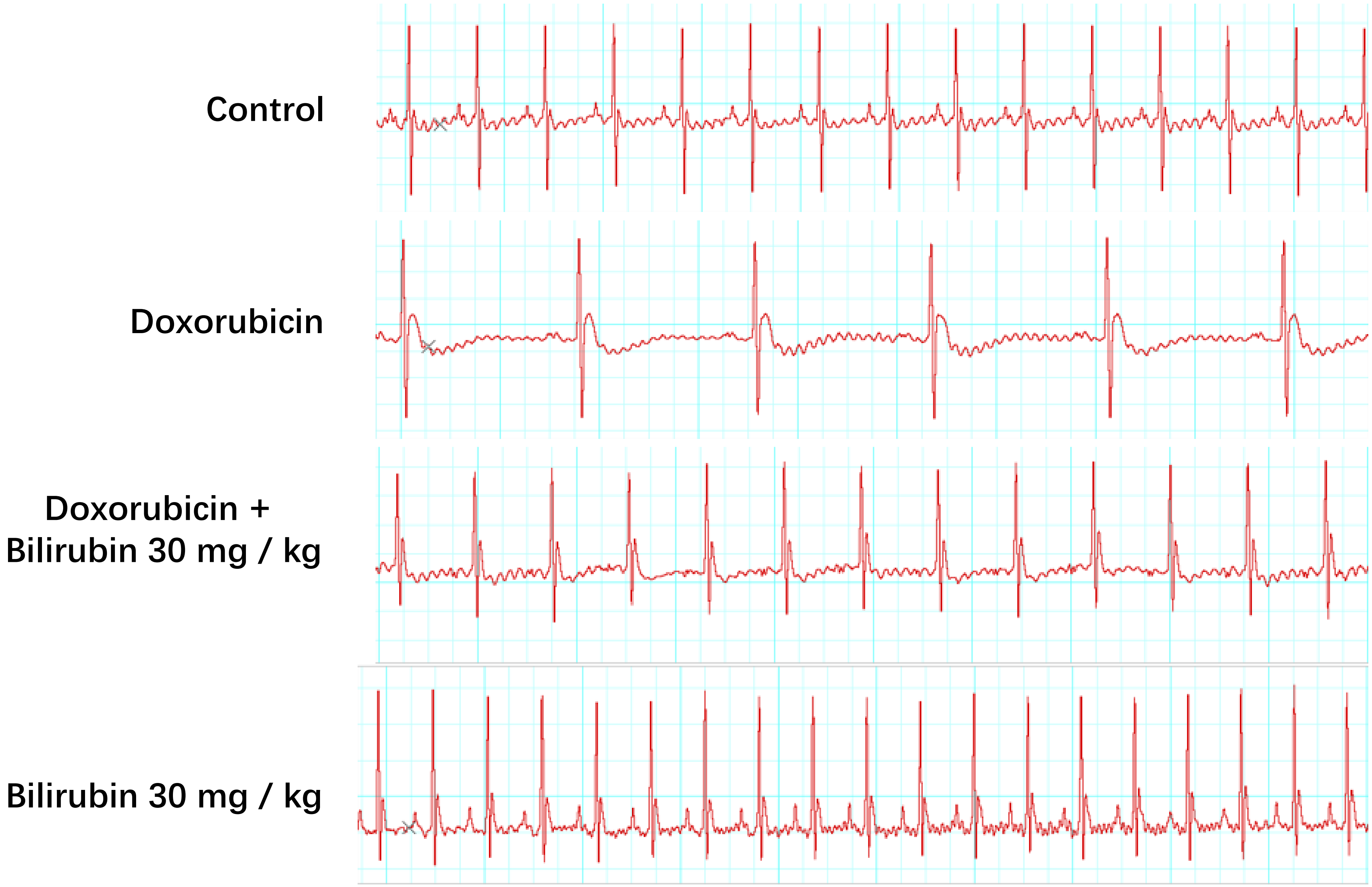
Control
Doxorubicin
Doxorubicin +
Bilirubin 30 mg / kg
Bilirubin 30 mg / kg

Figure 3A

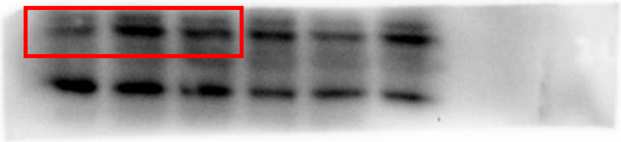

p-Cx43

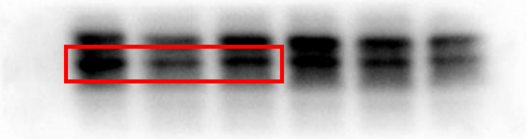

total-Cx43

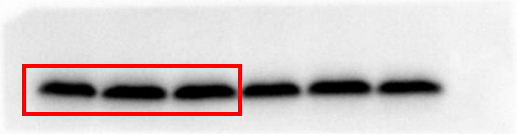

β -actin

Figure 3B

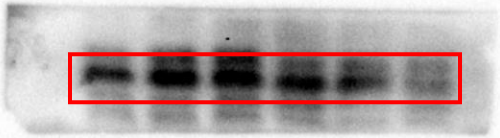

p-Cx43

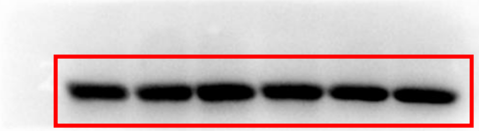

β -actin

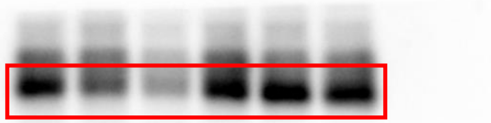

total-Cx43

Figure 3C

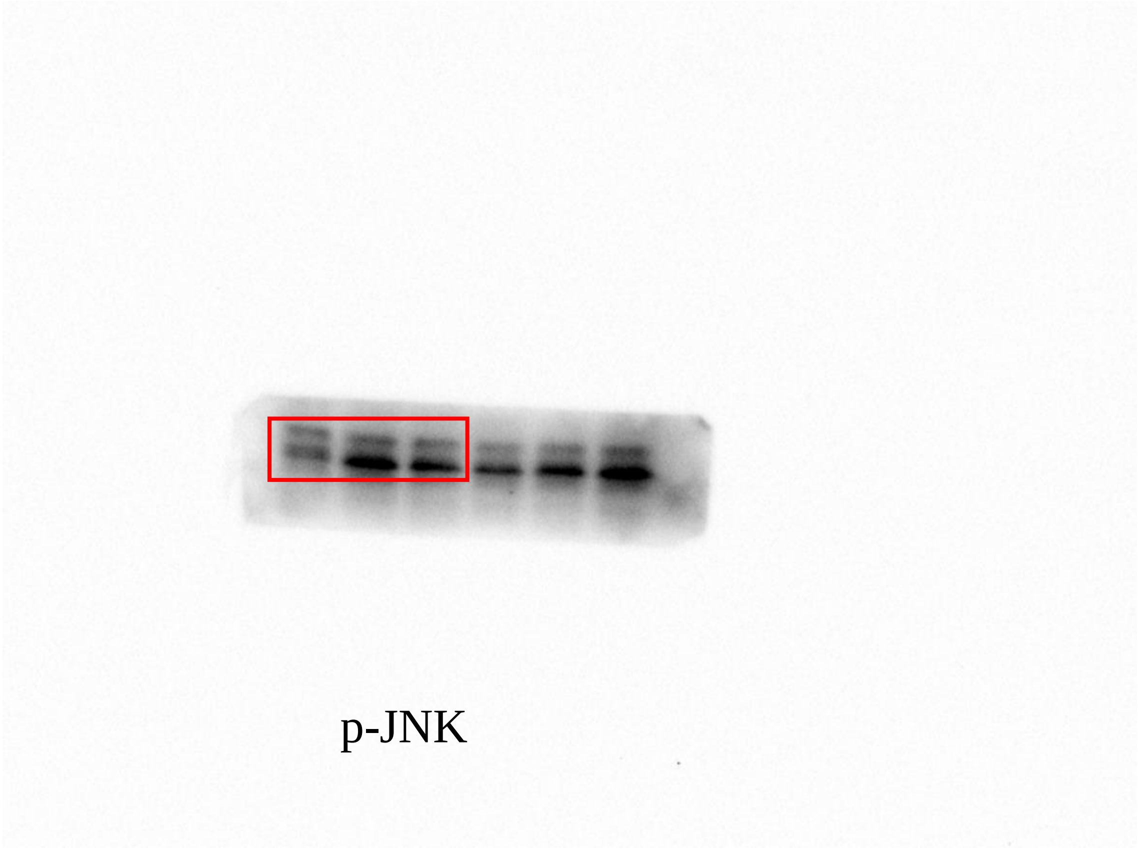

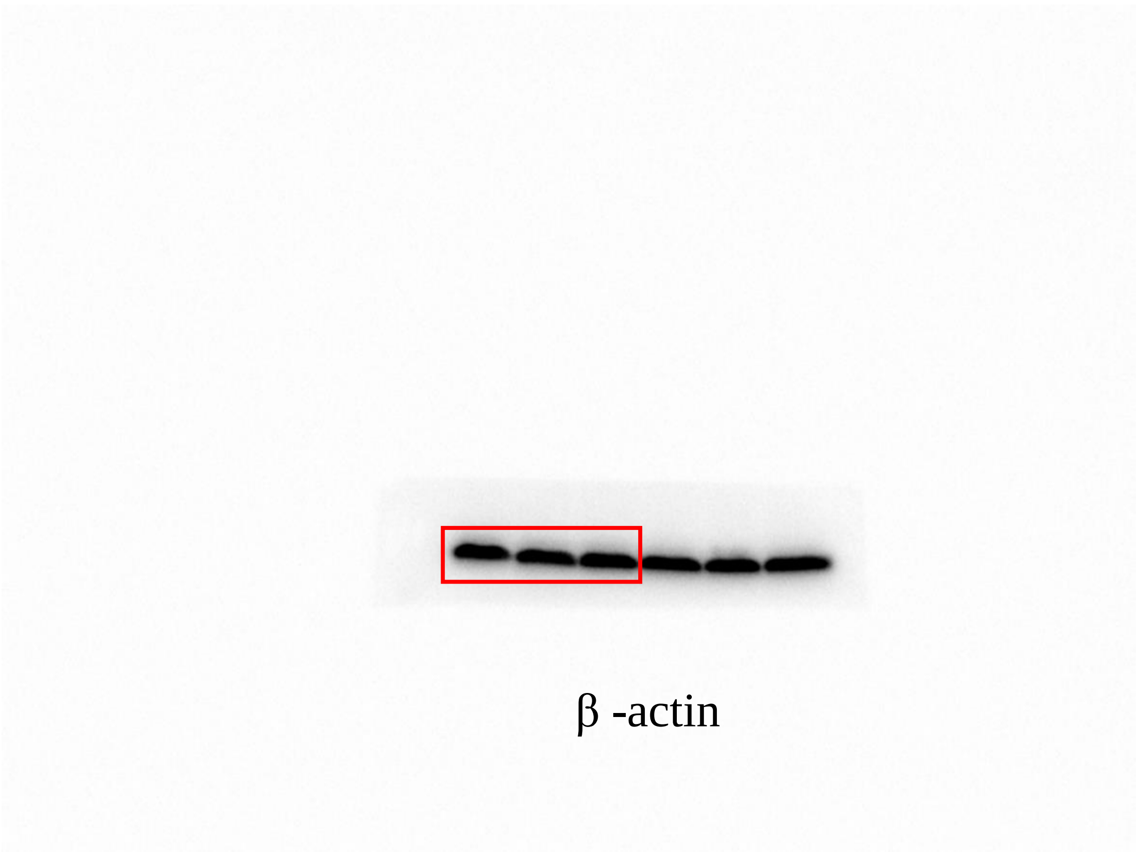

Figure 3D

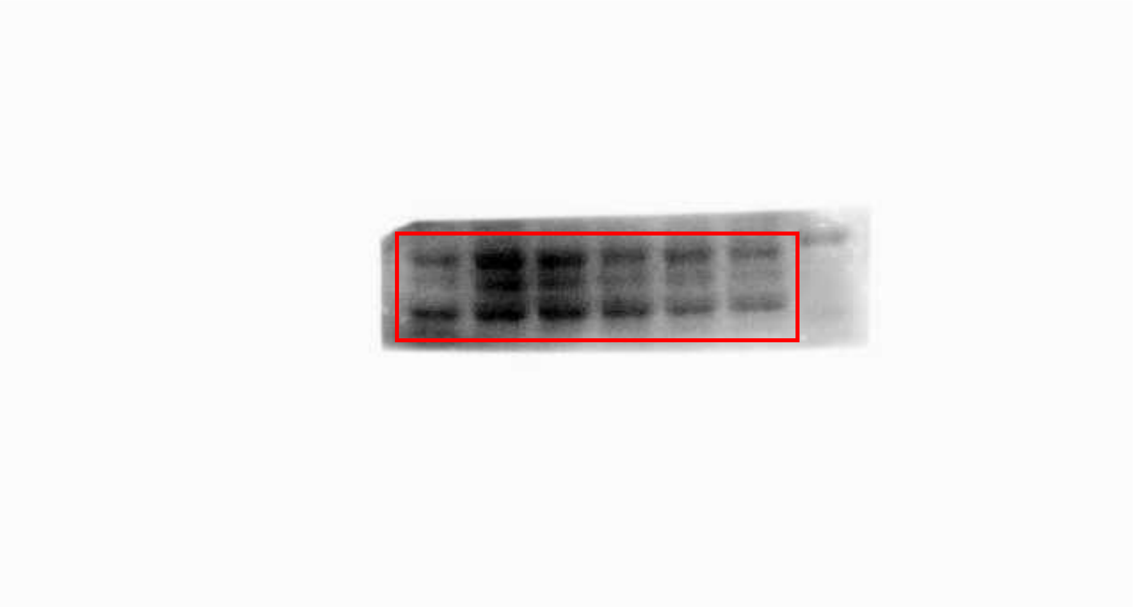

p-JNK

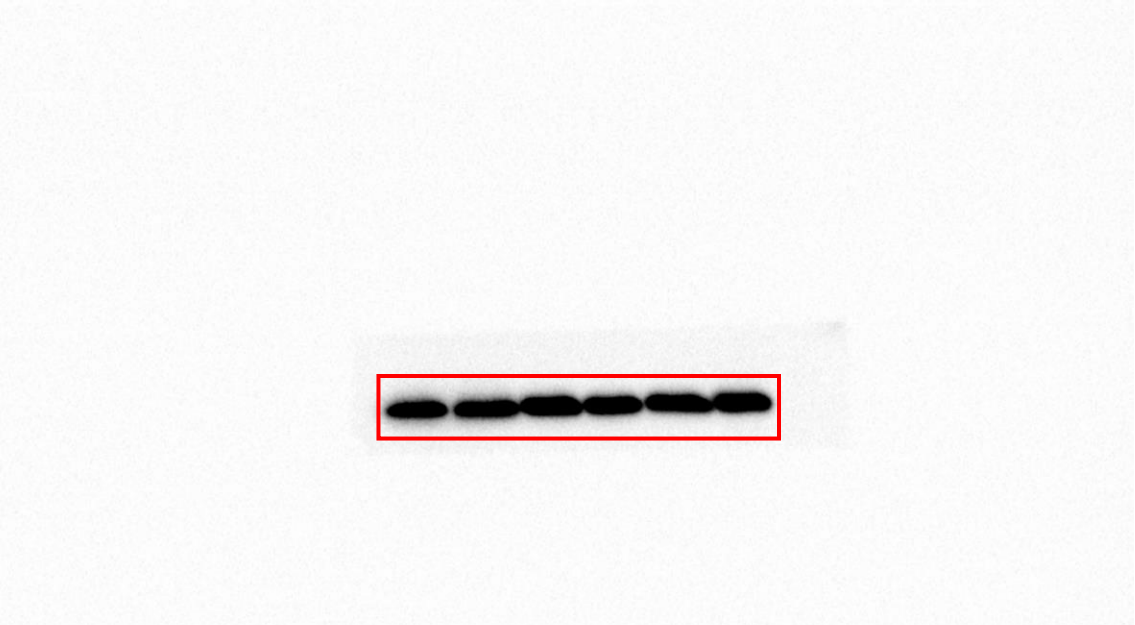

β -actin

Figure 4A

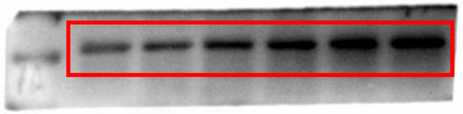

p-AMPK

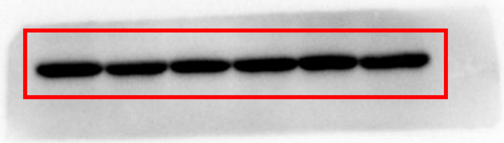

β -actin

Figure 4B

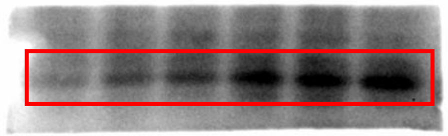

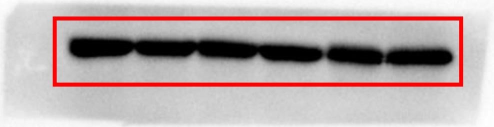

Figure 4C

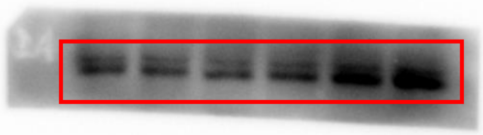

p-AMPK

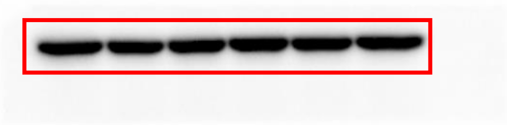

β -actin

Figure 4D

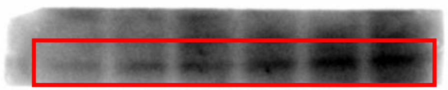

SOCS3

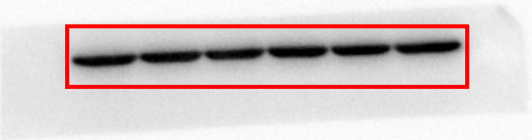

β -actin

Figure 5B

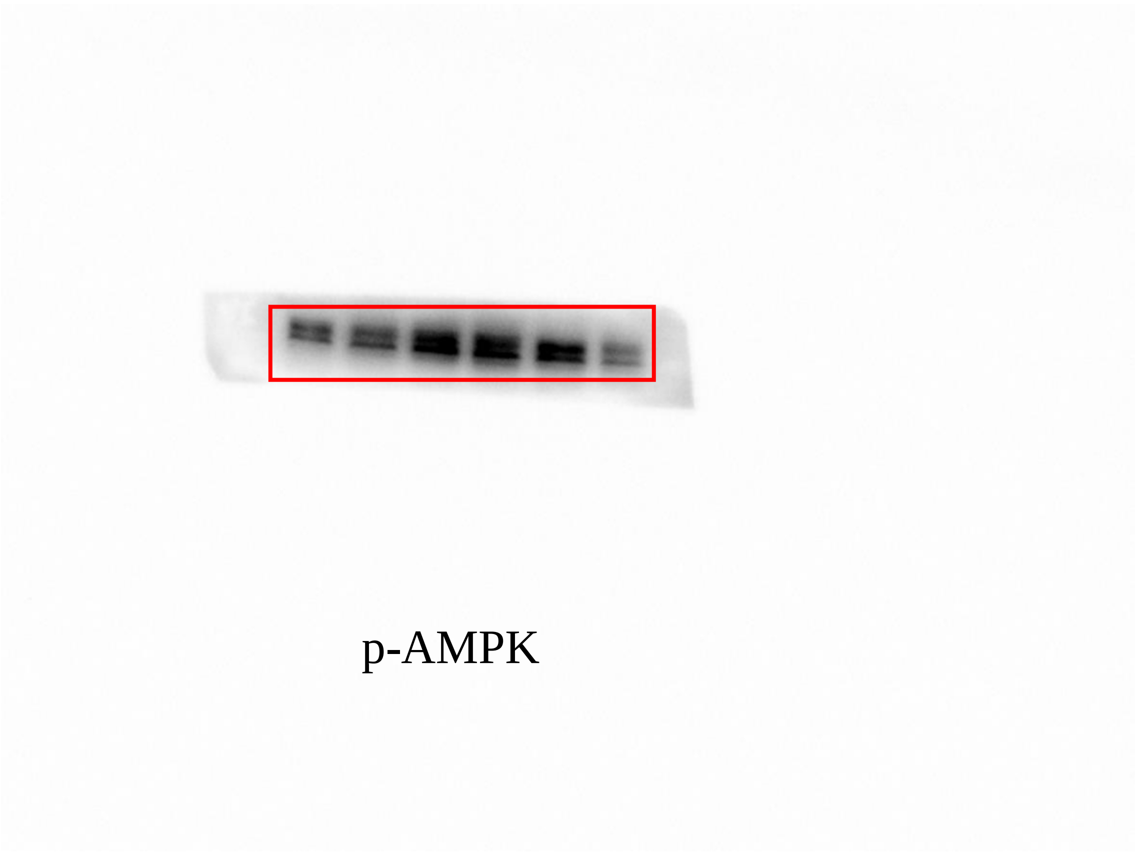

p-AMPK

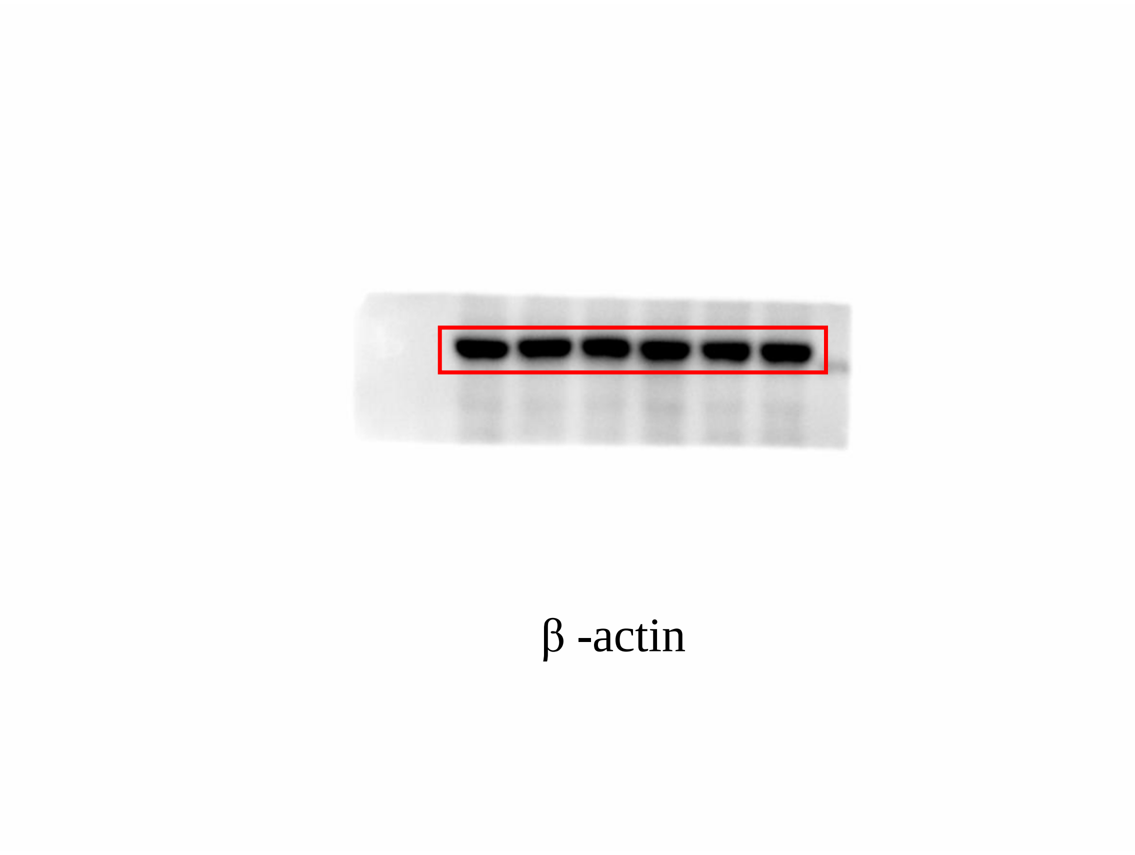

β -actin

Figure 5C

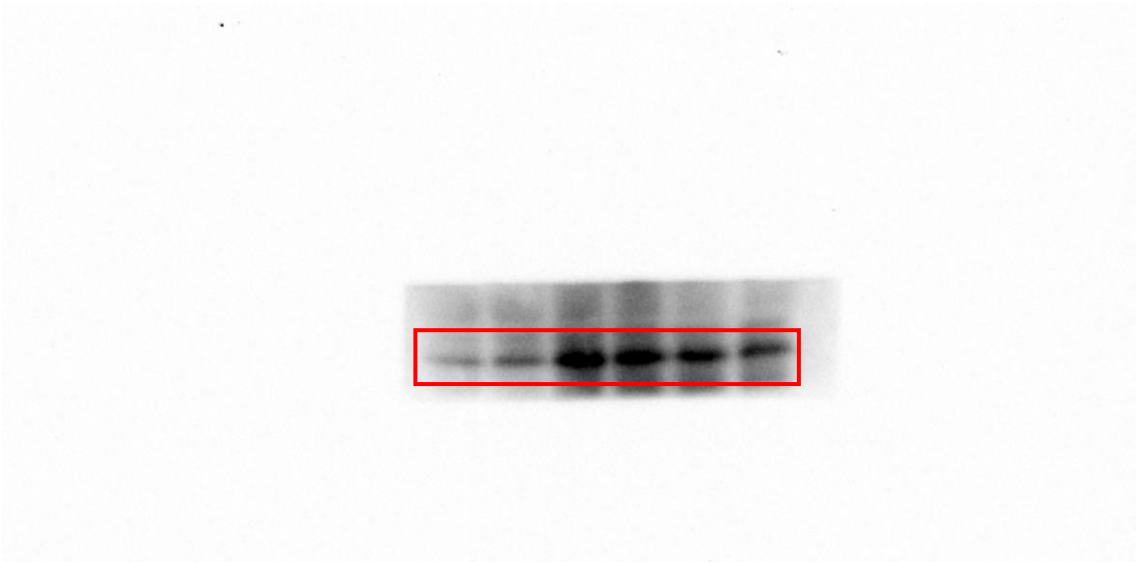

SOCS3

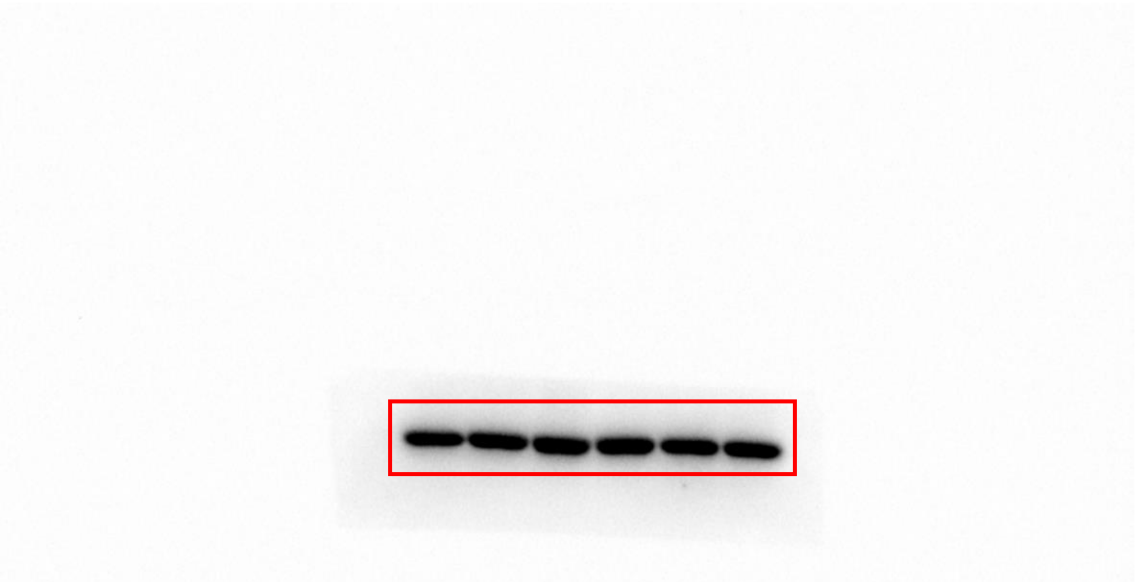

β -actin

Figure 5D

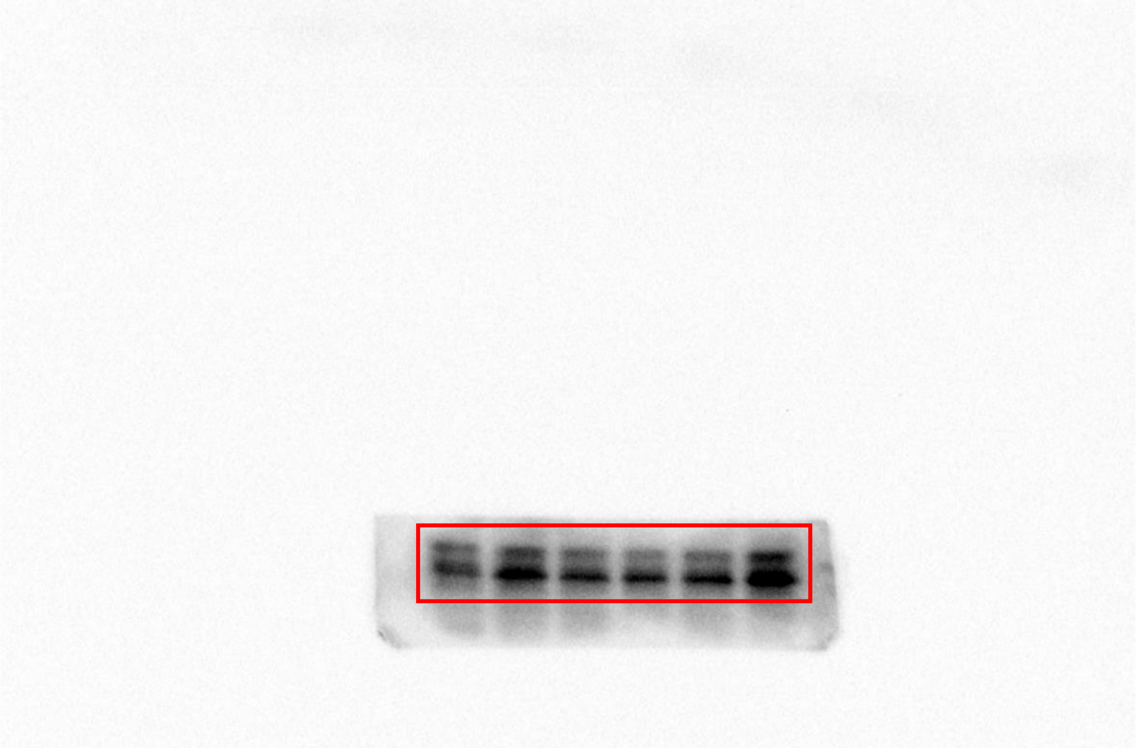

p-JNK

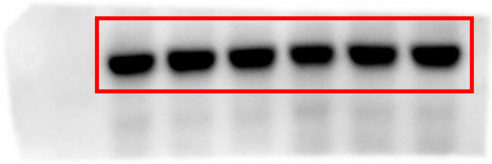

β -actin

Figure 5E

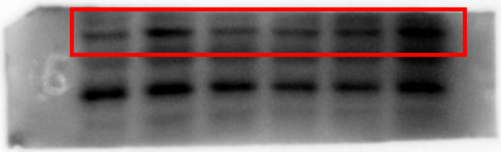

p-Cx43

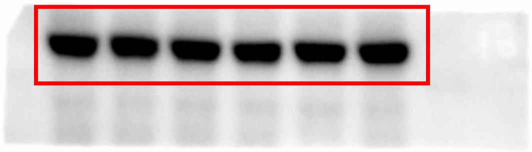

β -actin

Figure 6A

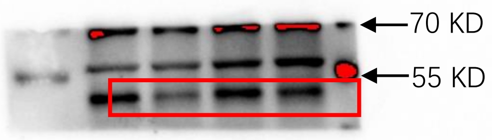

p-AMPK

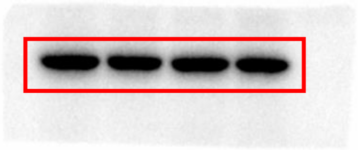

β -actin

Figure 6B

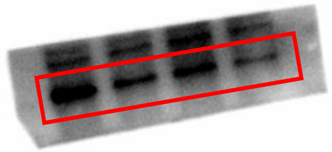

Axl

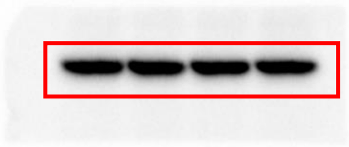

β -actin

Figure 6C

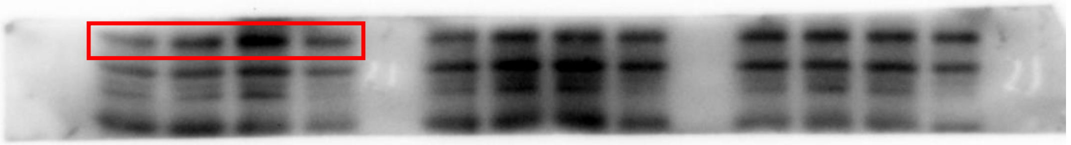

SOCS3

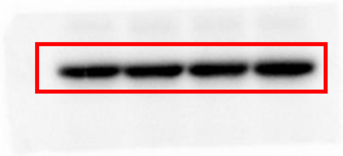

β -actin

Figure 6D

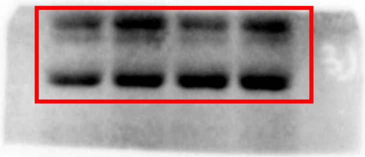

p-JNK

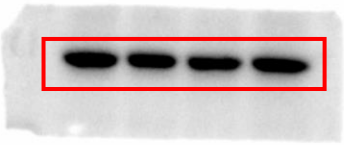

β -actin

Figure 6E

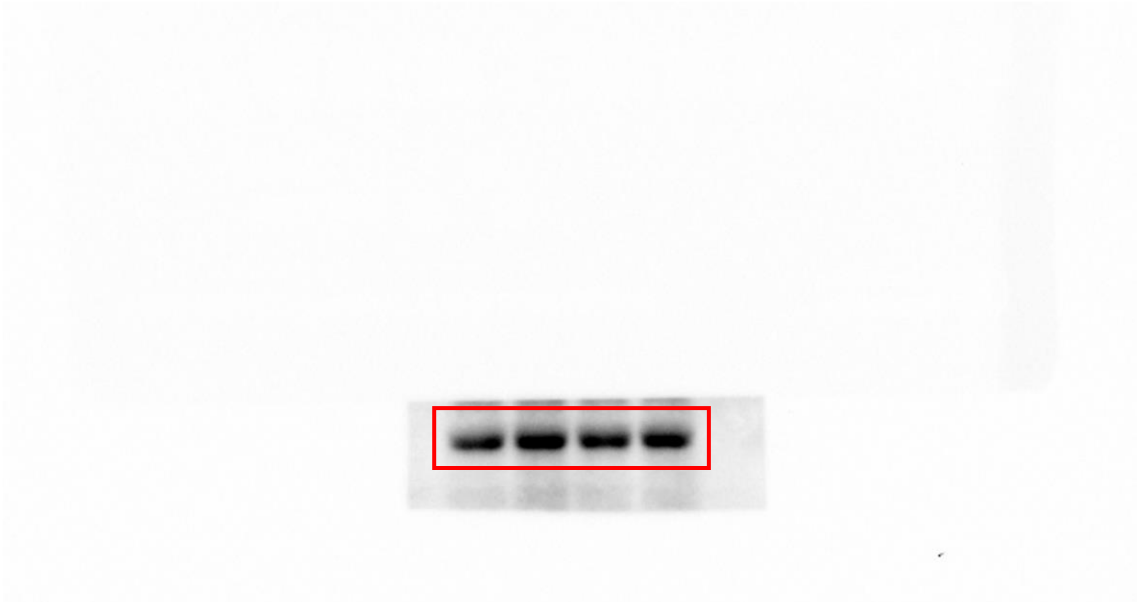

p-Cx43

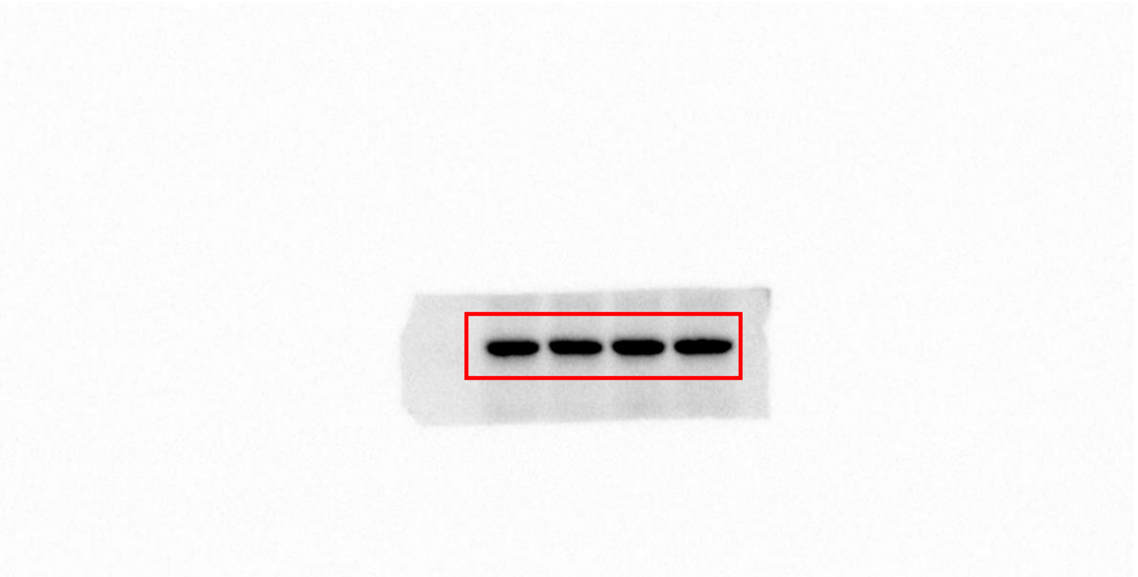

β -actin

Figure 6F

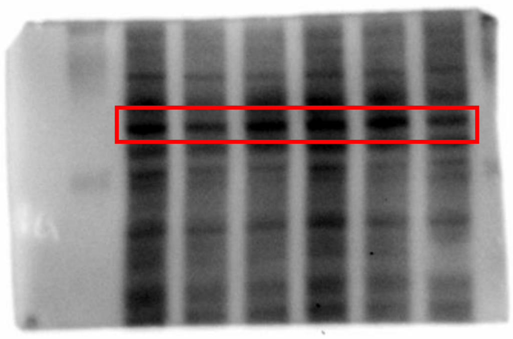

Axl

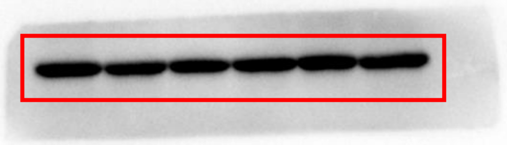

β -actin

Figure 6G

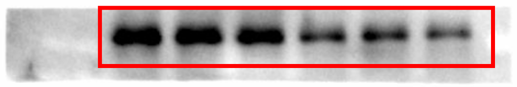

Axl

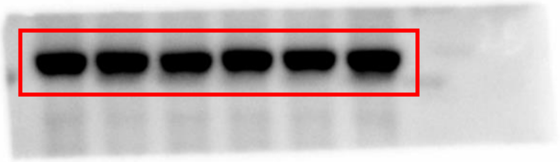

β -actin

Figure 6H

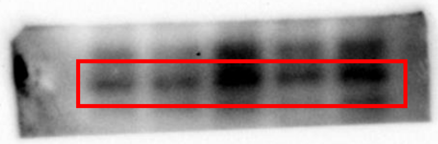

SOCS3

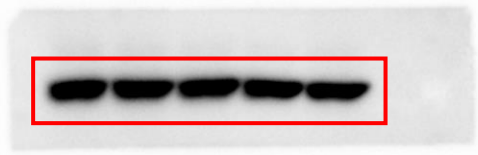

β -actin

Figure 6I

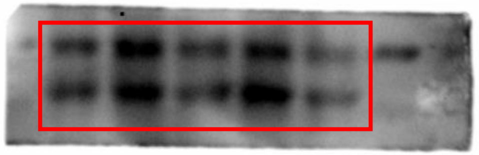

p-JNK

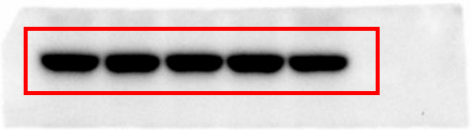

β -actin

Figure 6J

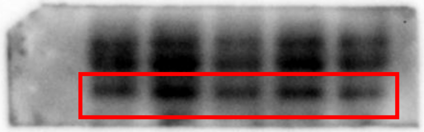

p-Cx43

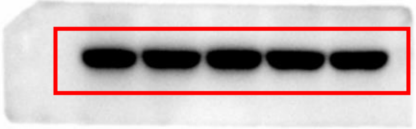

β -actin
