# Supplementary figures and images for: Bilirubin Improves Gap Junction to Alleviate Doxorubicin-Induced Cardiotoxicity by Regulating AMPK-Axl-SOCS3-Cx43 Axis
Source: Front Pharmacol. 2022 Apr 25;13:828890. doi: 10.3389/fphar.2022.828890 (PMC9082937; doi:10.3389/fphar.2022.828890)

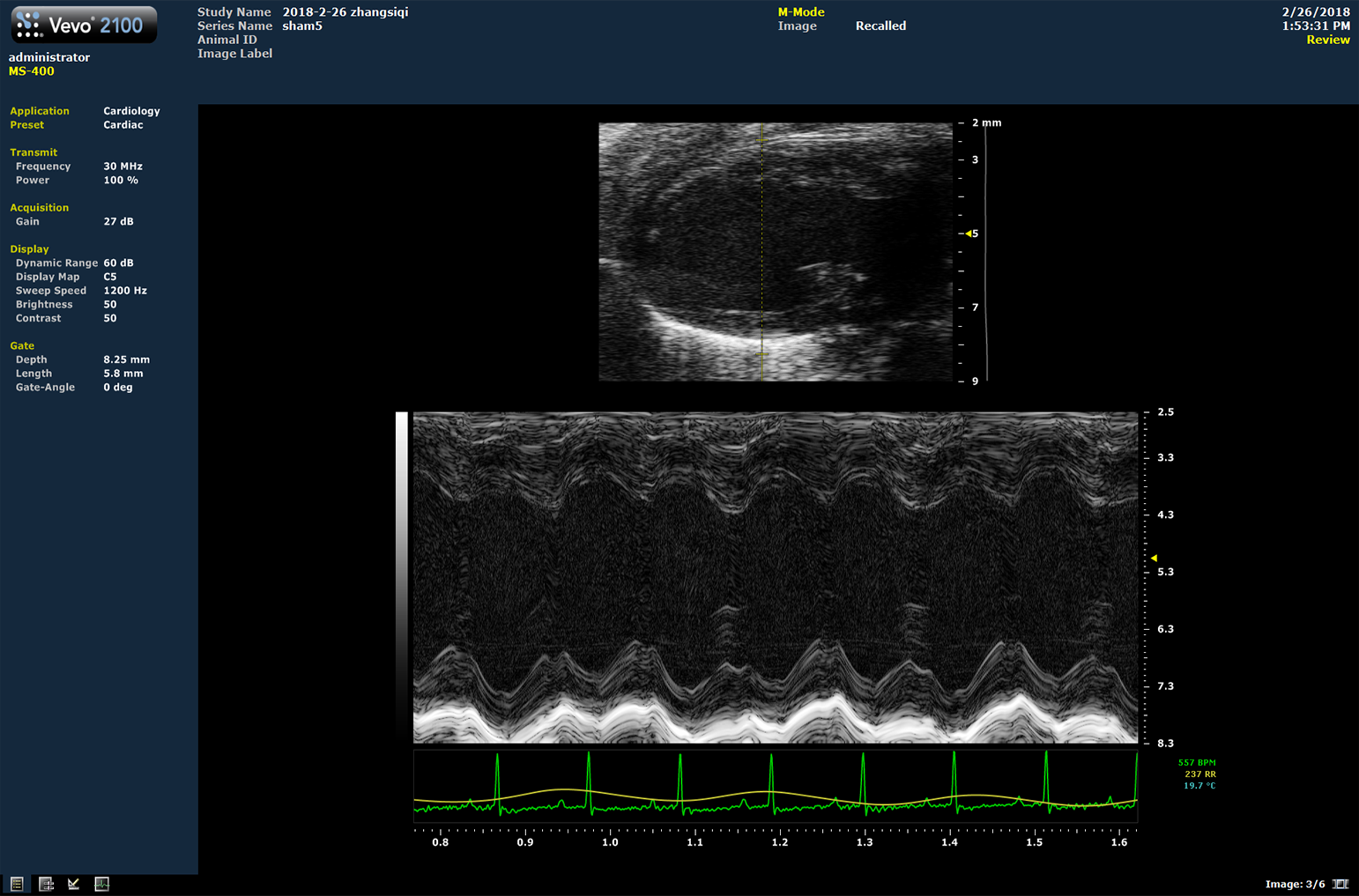

Supplement: Supplementary file 1 [file DataSheet1.ZIP › Original pictures for figures/1/control.tif]

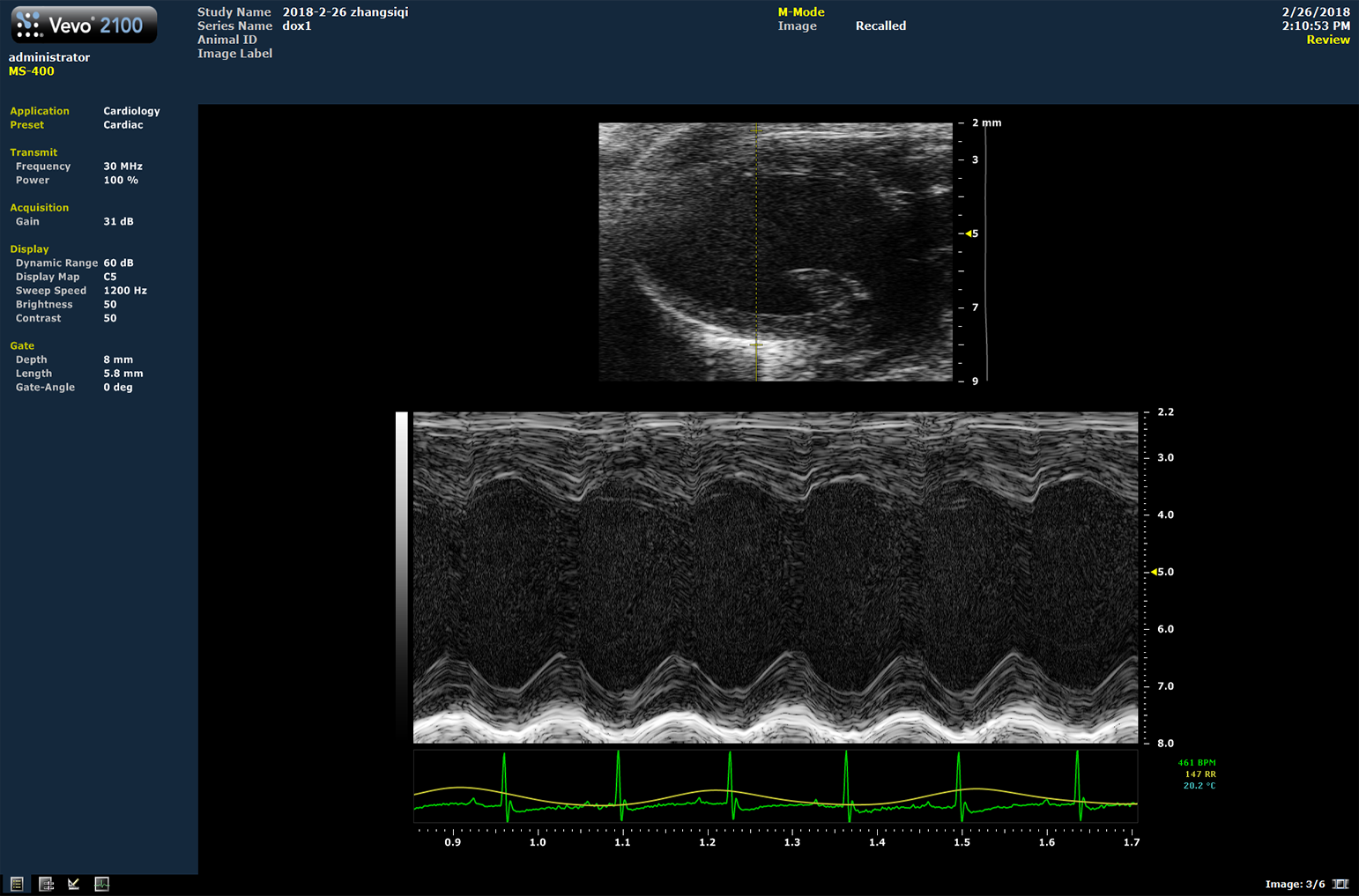

Supplement: Supplementary file 1 [file DataSheet1.ZIP › Original pictures for figures/1/dox.tif]

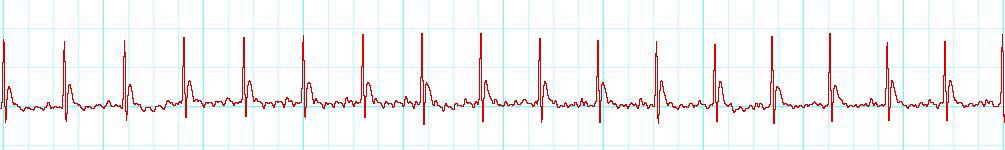

Supplement: Supplementary file 1 [file DataSheet1.ZIP › Original pictures for figures/1/ECG Control.jpg]

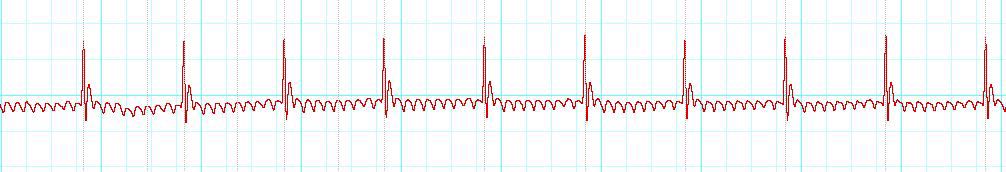

Supplement: Supplementary file 1 [file DataSheet1.ZIP › Original pictures for figures/1/ECG dox.jpg]

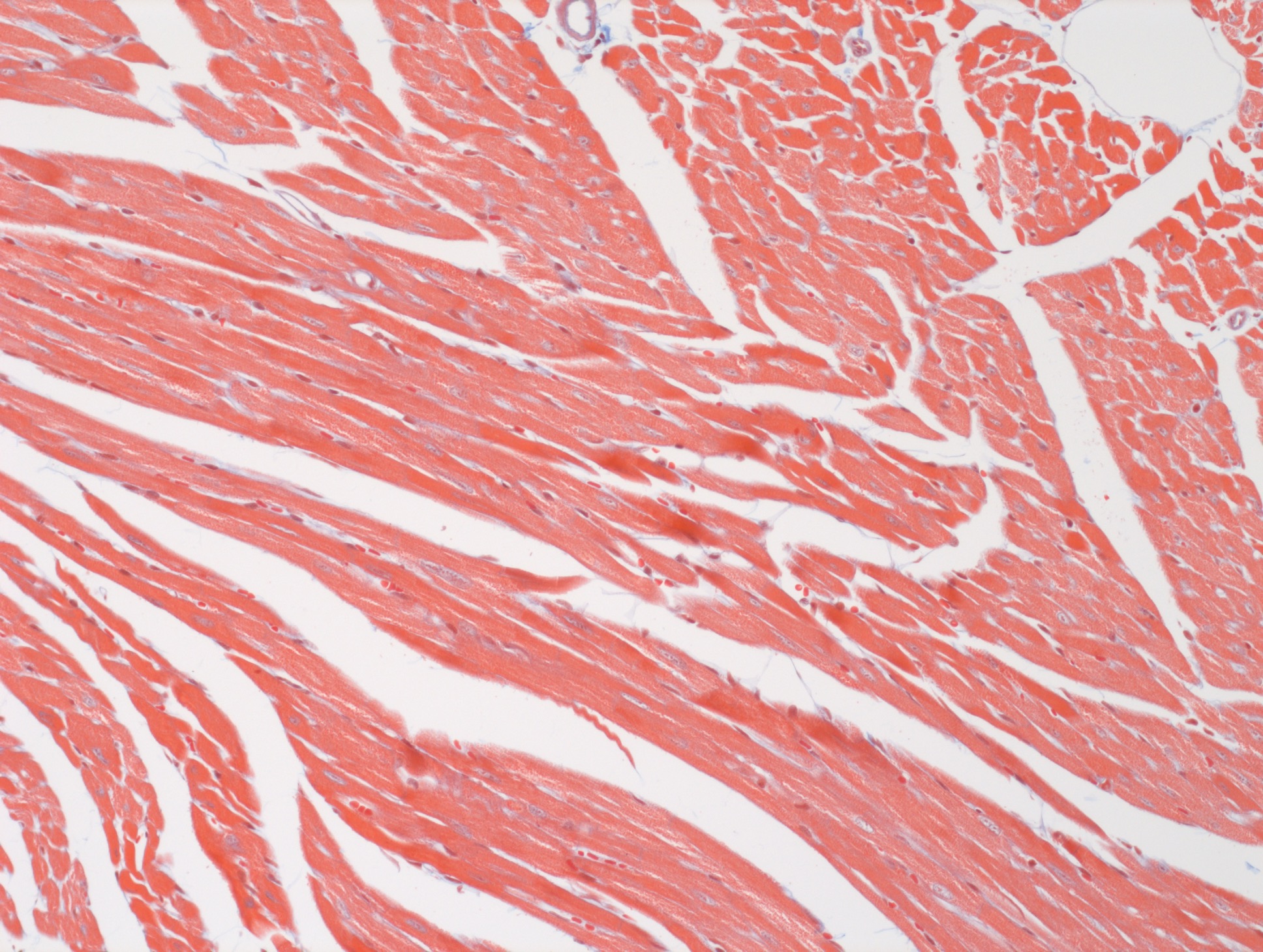

Supplement: Supplementary file 1 [file DataSheet1.ZIP › Original pictures for figures/1/masson control.jpg]

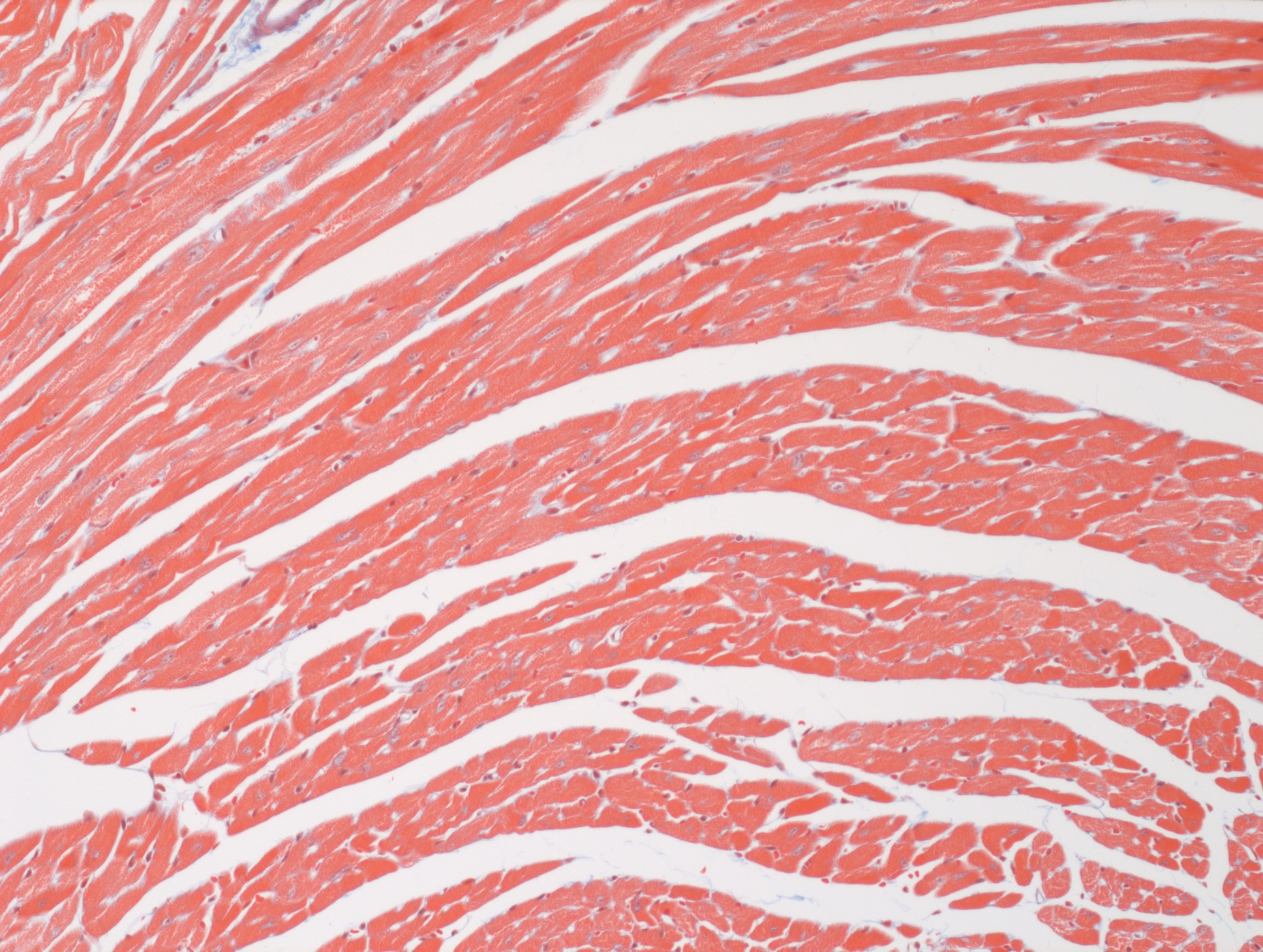

Supplement: Supplementary file 1 [file DataSheet1.ZIP › Original pictures for figures/1/masson dox.jpg]

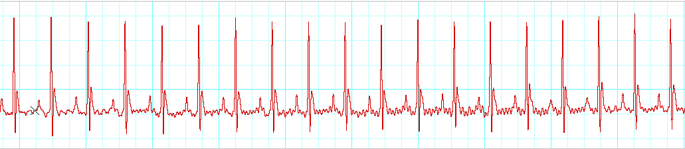

Supplement: Supplementary file 1 [file DataSheet1.ZIP › Original pictures for figures/2/ECG br 30.tif]

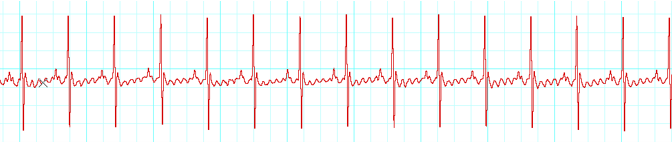

Supplement: Supplementary file 1 [file DataSheet1.ZIP › Original pictures for figures/2/ECG Control.tif]

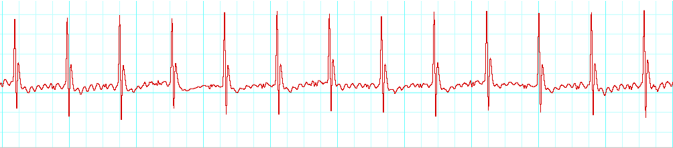

Supplement: Supplementary file 1 [file DataSheet1.ZIP › Original pictures for figures/2/ECG dox+br30.tif]

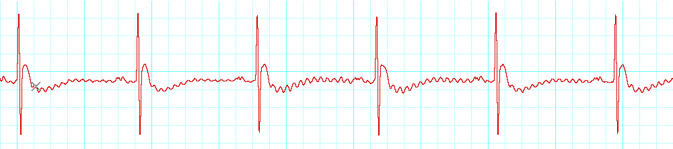

Supplement: Supplementary file 1 [file DataSheet1.ZIP › Original pictures for figures/2/ECG dox.tif]

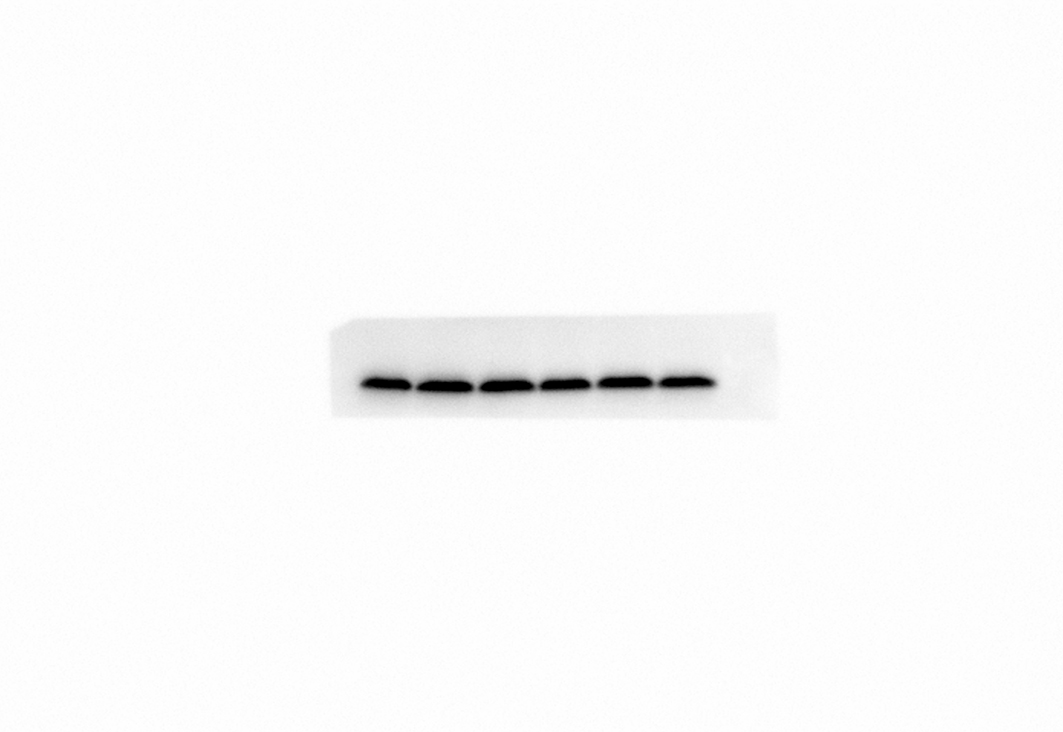

Supplement: Supplementary file 1 [file DataSheet1.ZIP › Original pictures for figures/3/3A actin.jpg]

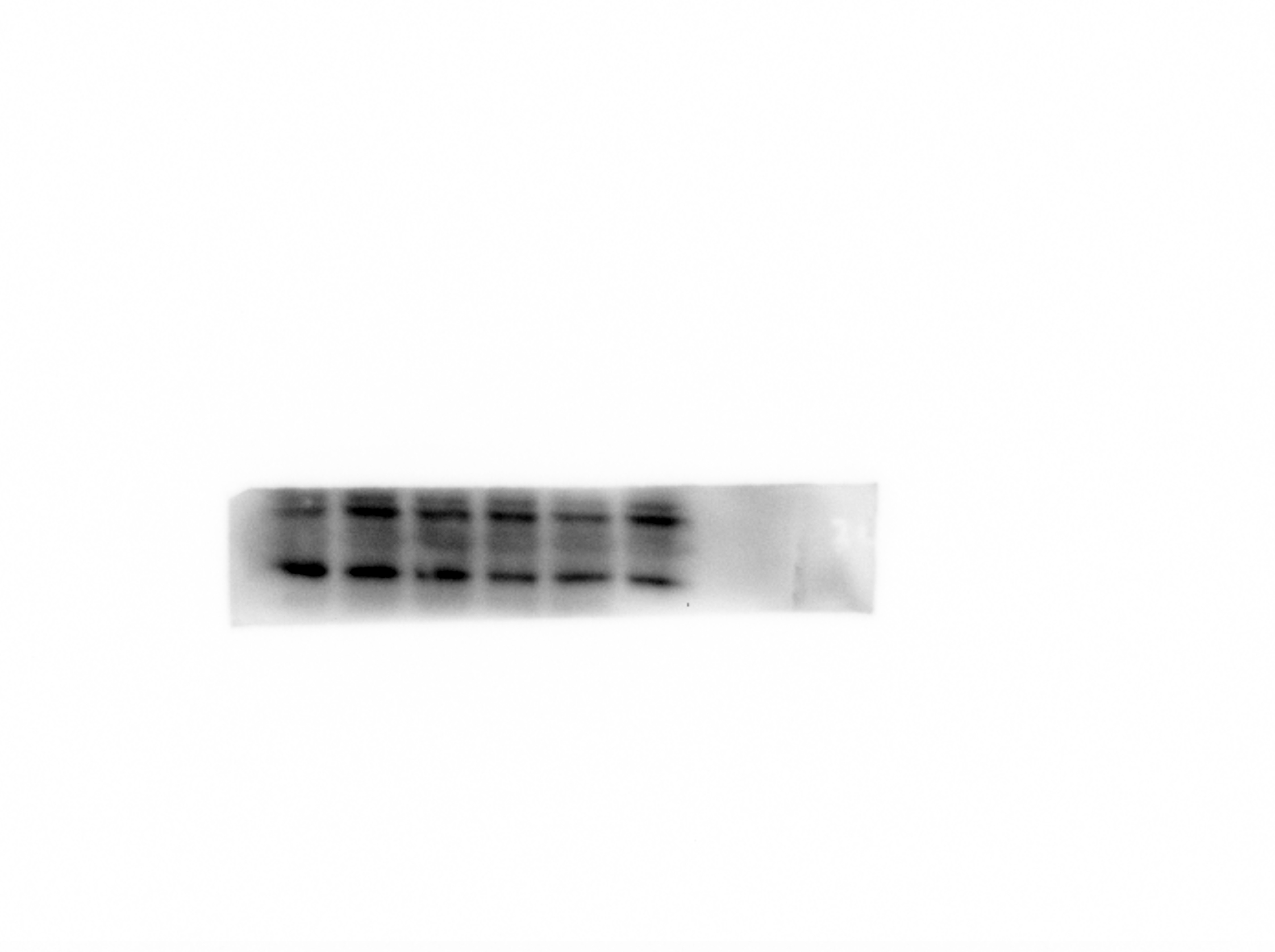

Supplement: Supplementary file 1 [file DataSheet1.ZIP › Original pictures for figures/3/3A P-CX-43.jpg]

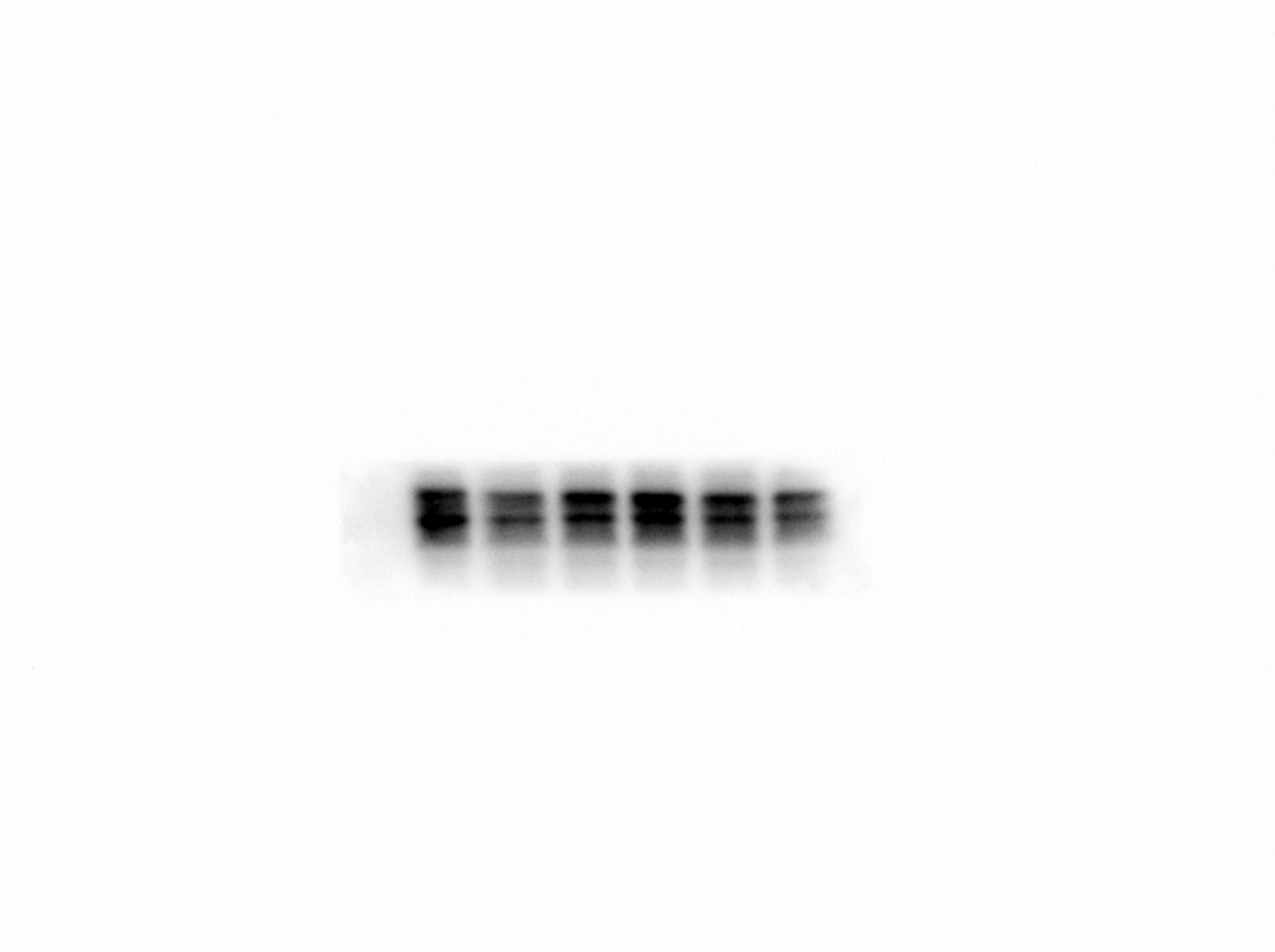

Supplement: Supplementary file 1 [file DataSheet1.ZIP › Original pictures for figures/3/3A total Cx43.jpg]

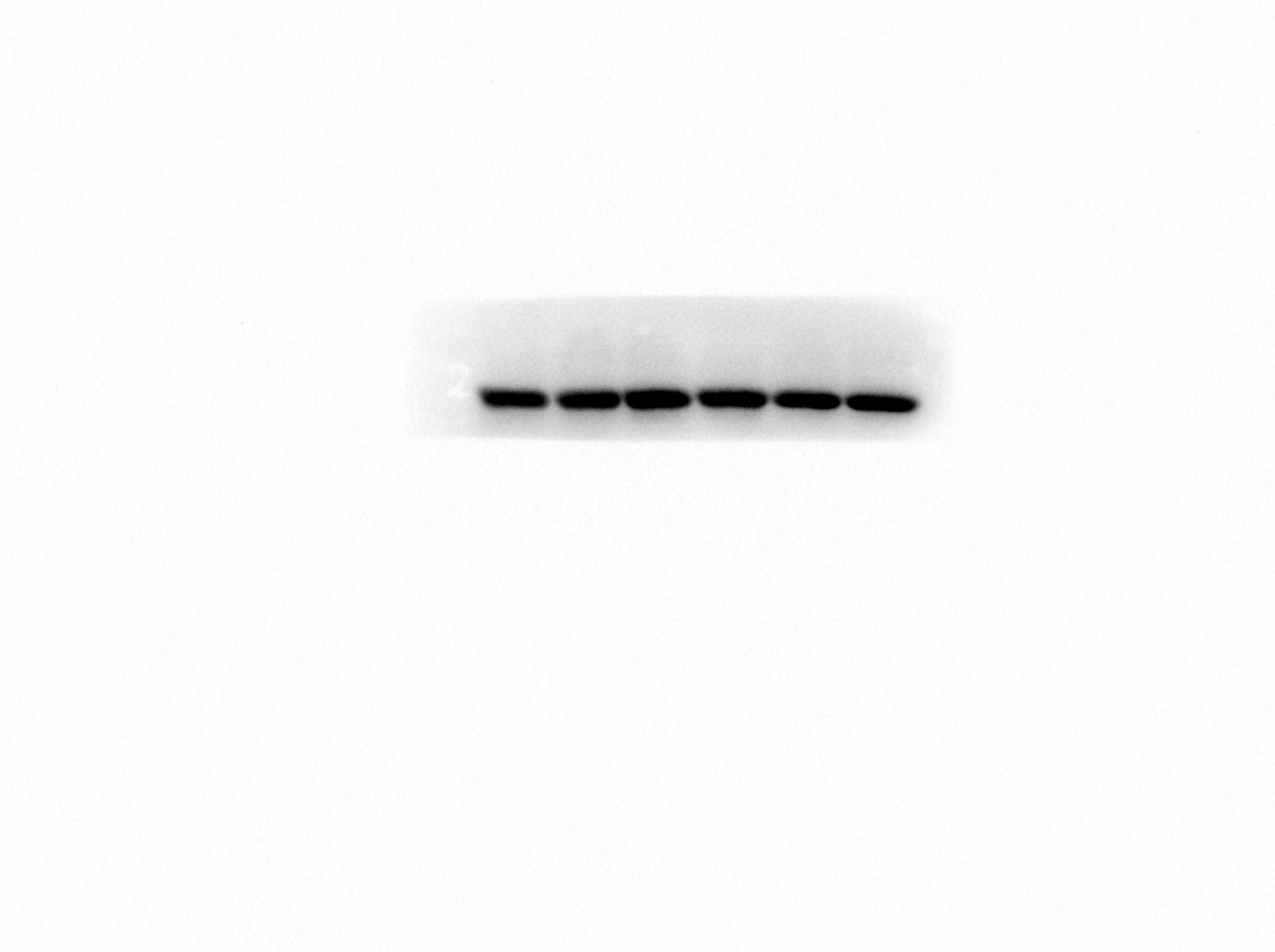

Supplement: Supplementary file 1 [file DataSheet1.ZIP › Original pictures for figures/3/3B actin.jpg]

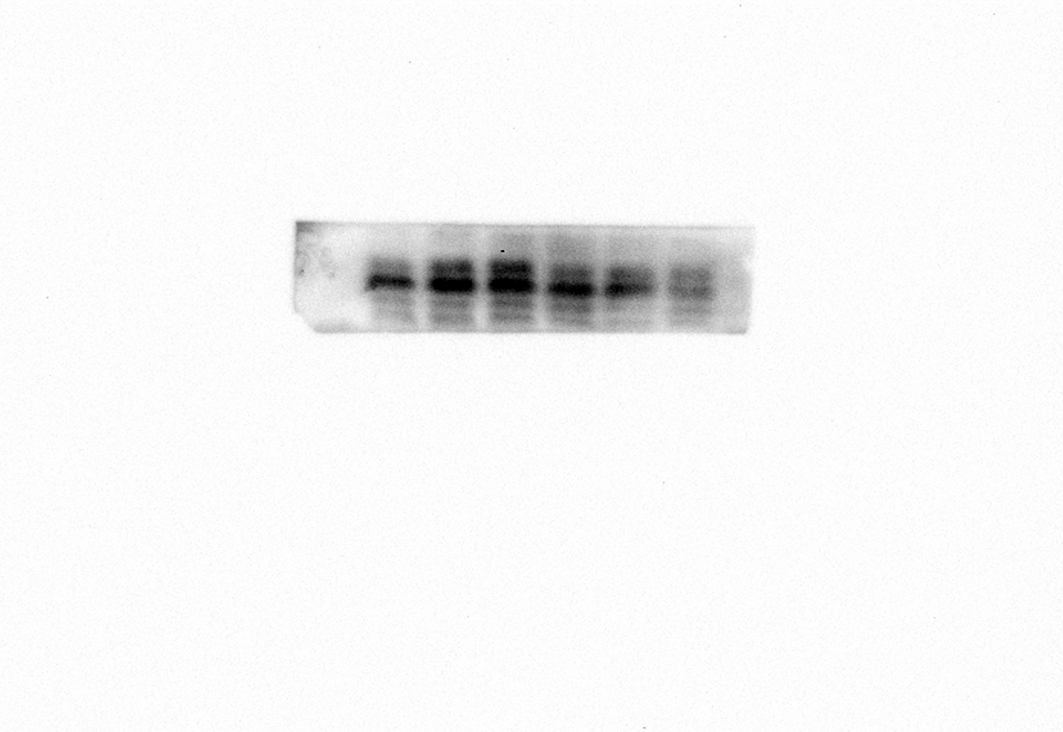

Supplement: Supplementary file 1 [file DataSheet1.ZIP › Original pictures for figures/3/3B p-Cx43.jpg]

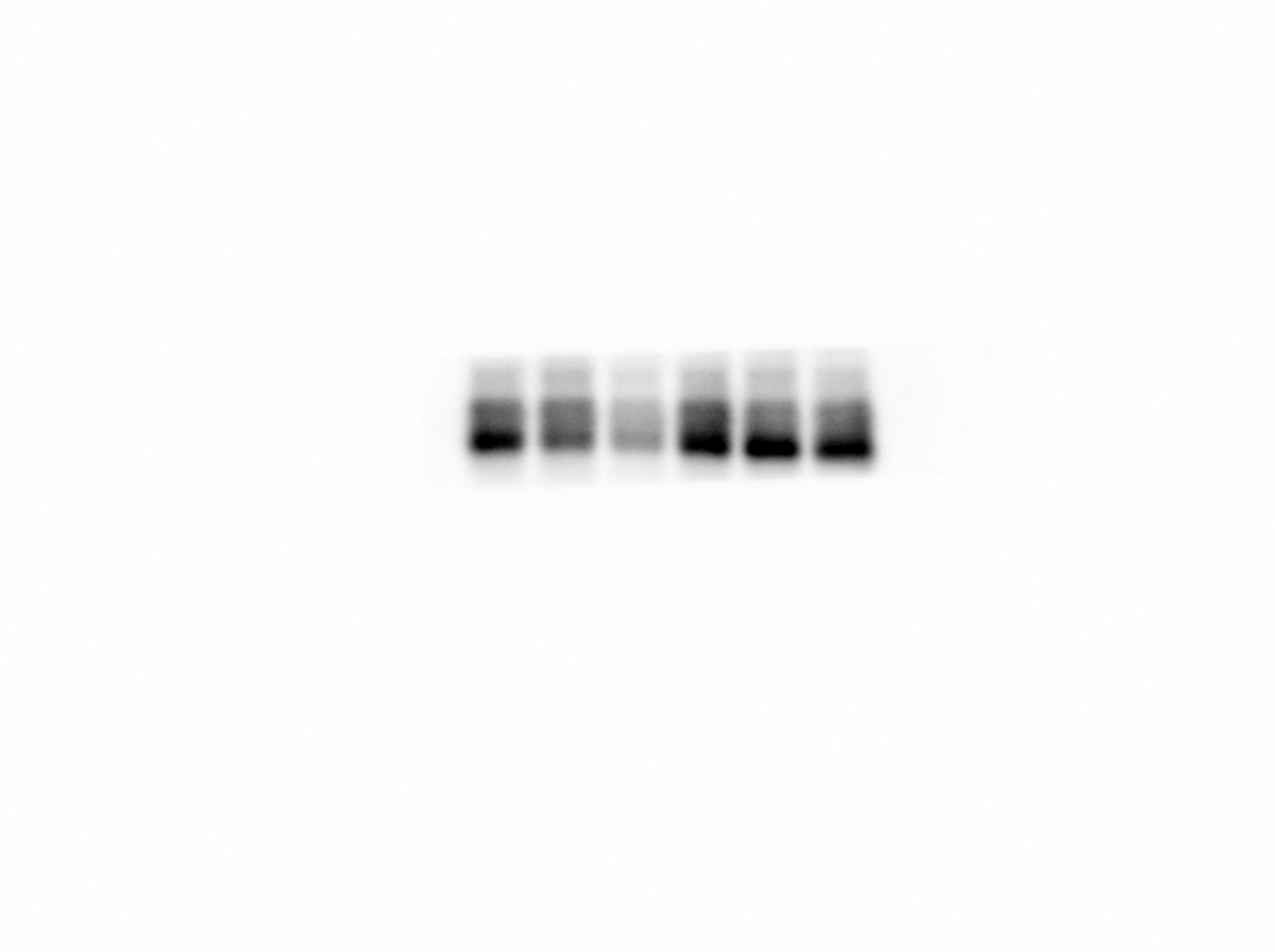

Supplement: Supplementary file 1 [file DataSheet1.ZIP › Original pictures for figures/3/3B total Cx43.jpg]

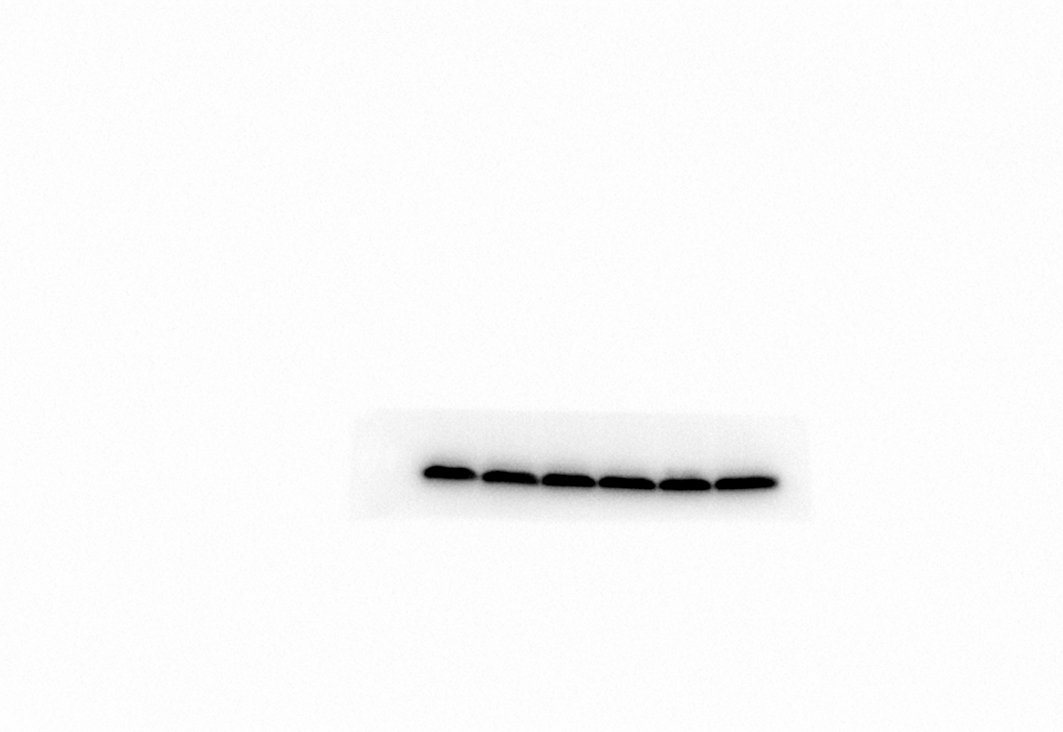

Supplement: Supplementary file 1 [file DataSheet1.ZIP › Original pictures for figures/3/3C actin.jpg]

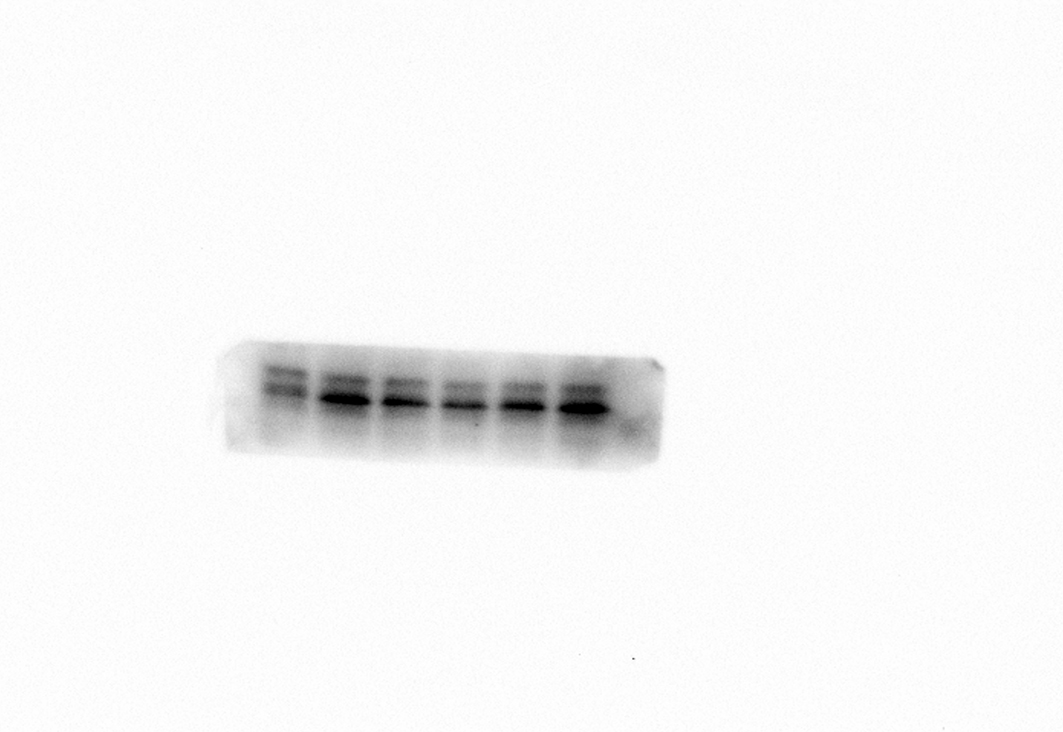

Supplement: Supplementary file 1 [file DataSheet1.ZIP › Original pictures for figures/3/3C p-JNK.jpg]

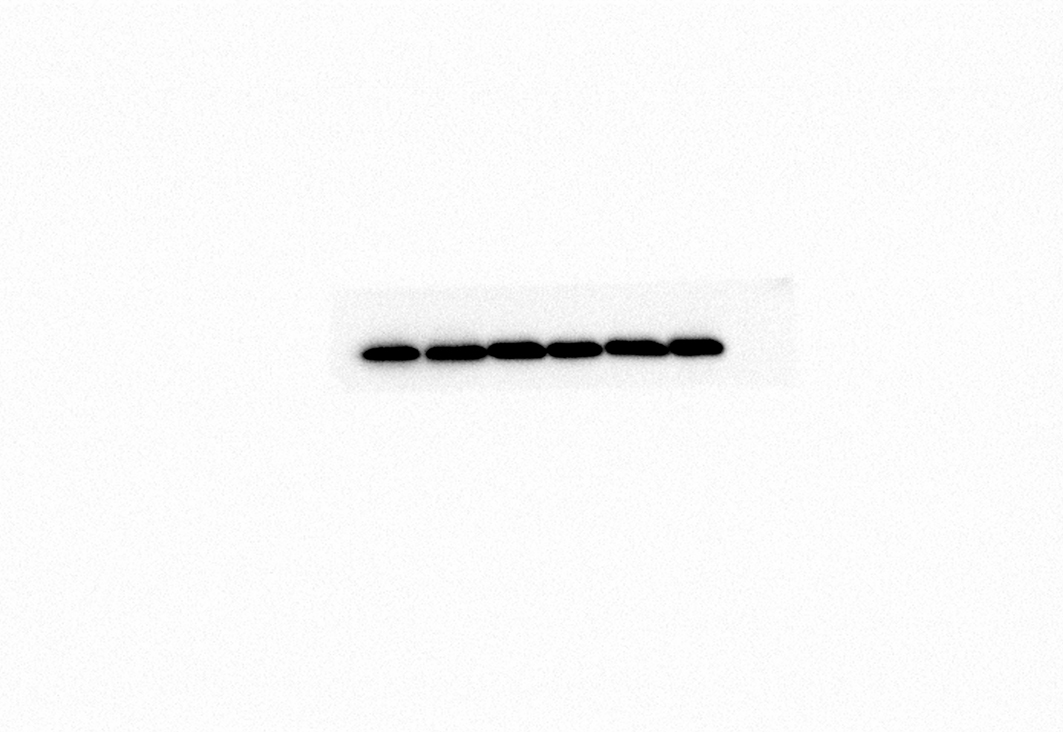

Supplement: Supplementary file 1 [file DataSheet1.ZIP › Original pictures for figures/3/3D actin.jpg]

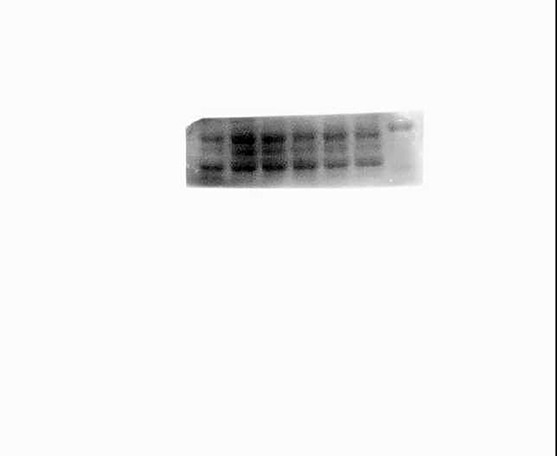

Supplement: Supplementary file 1 [file DataSheet1.ZIP › Original pictures for figures/3/3D p-JNK.jpg]

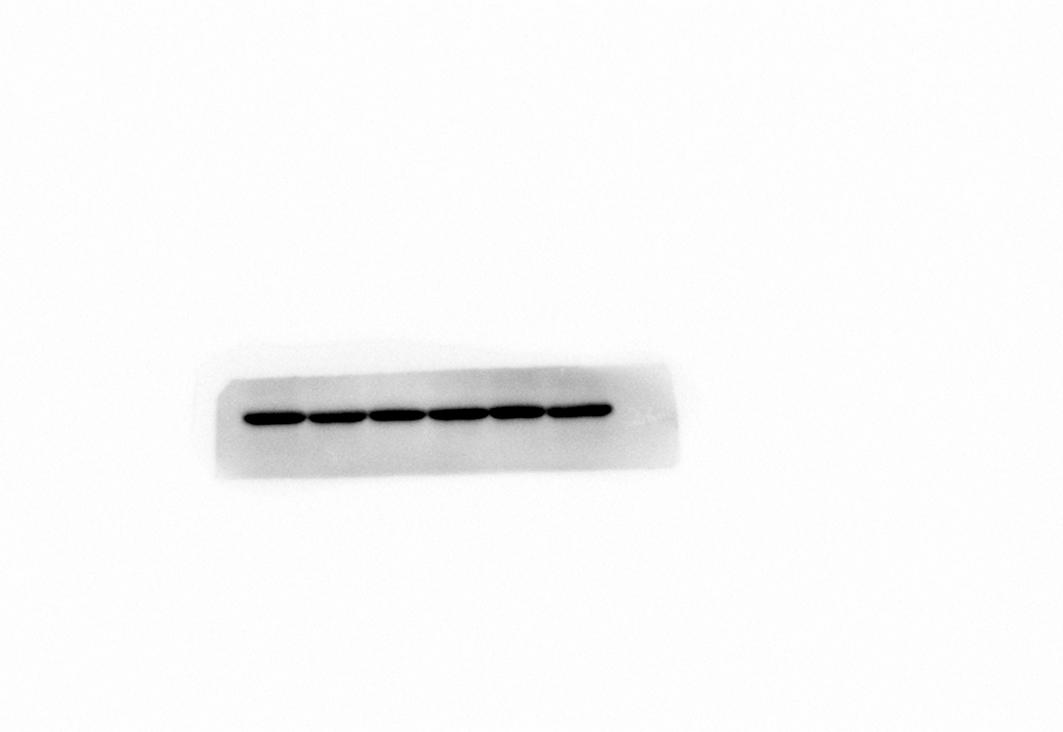

Supplement: Supplementary file 1 [file DataSheet1.ZIP › Original pictures for figures/4/4A actin.jpg]

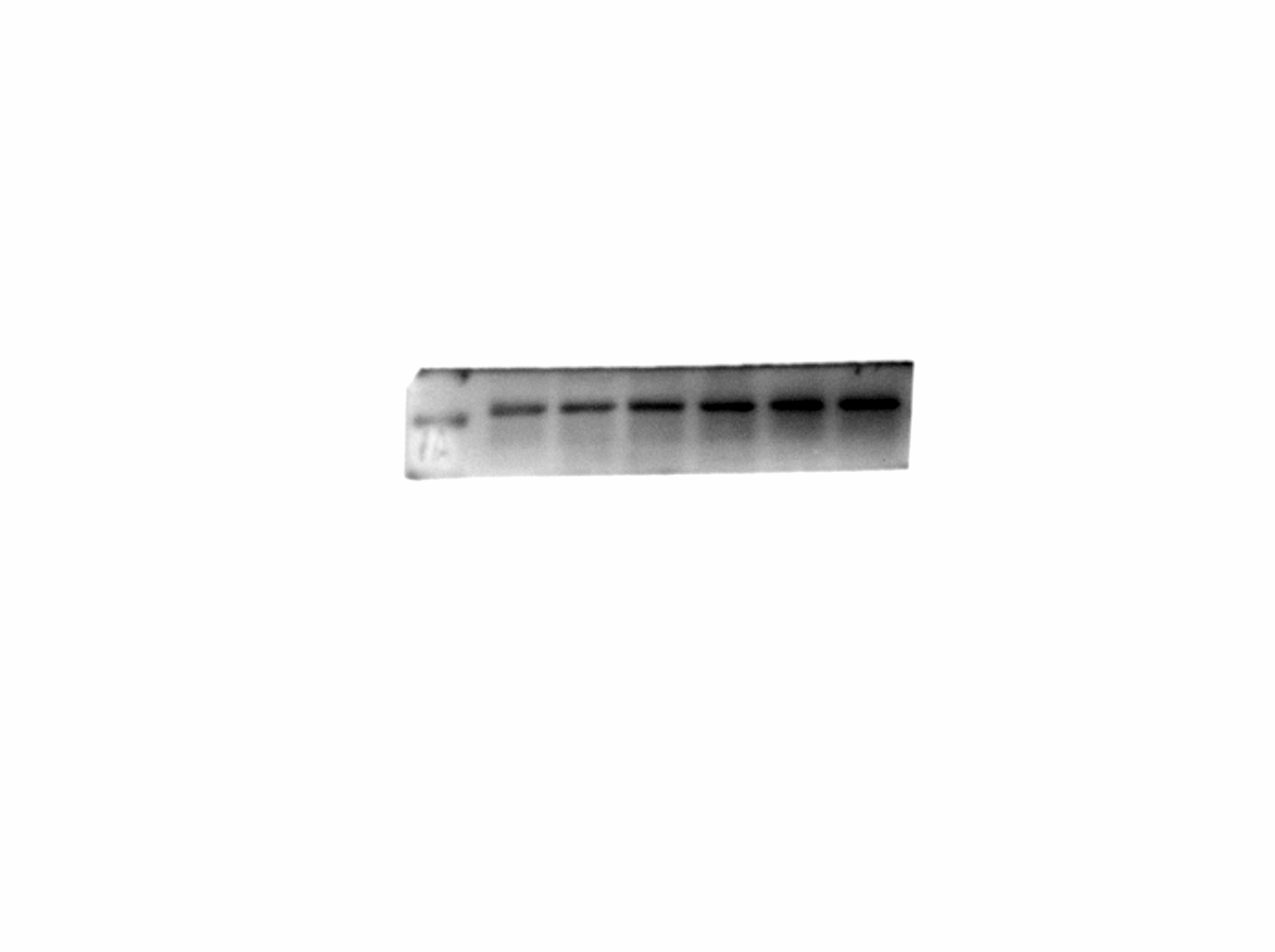

Supplement: Supplementary file 1 [file DataSheet1.ZIP › Original pictures for figures/4/4A p-AMPK.jpg]

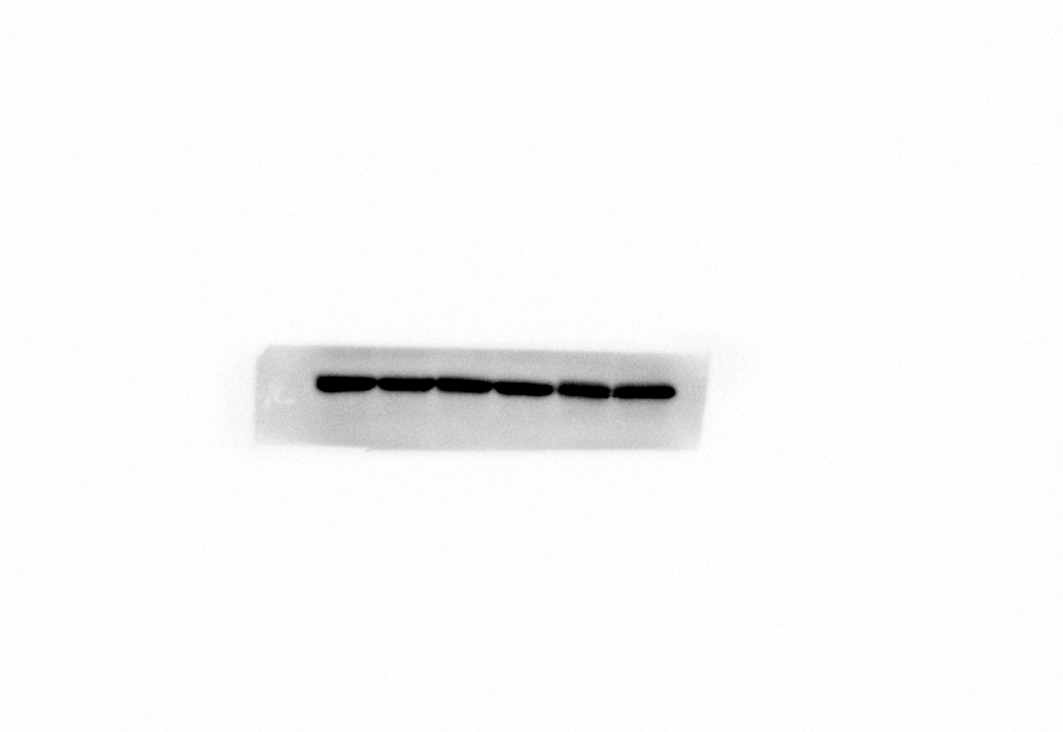

Supplement: Supplementary file 1 [file DataSheet1.ZIP › Original pictures for figures/4/4B actin.jpg]

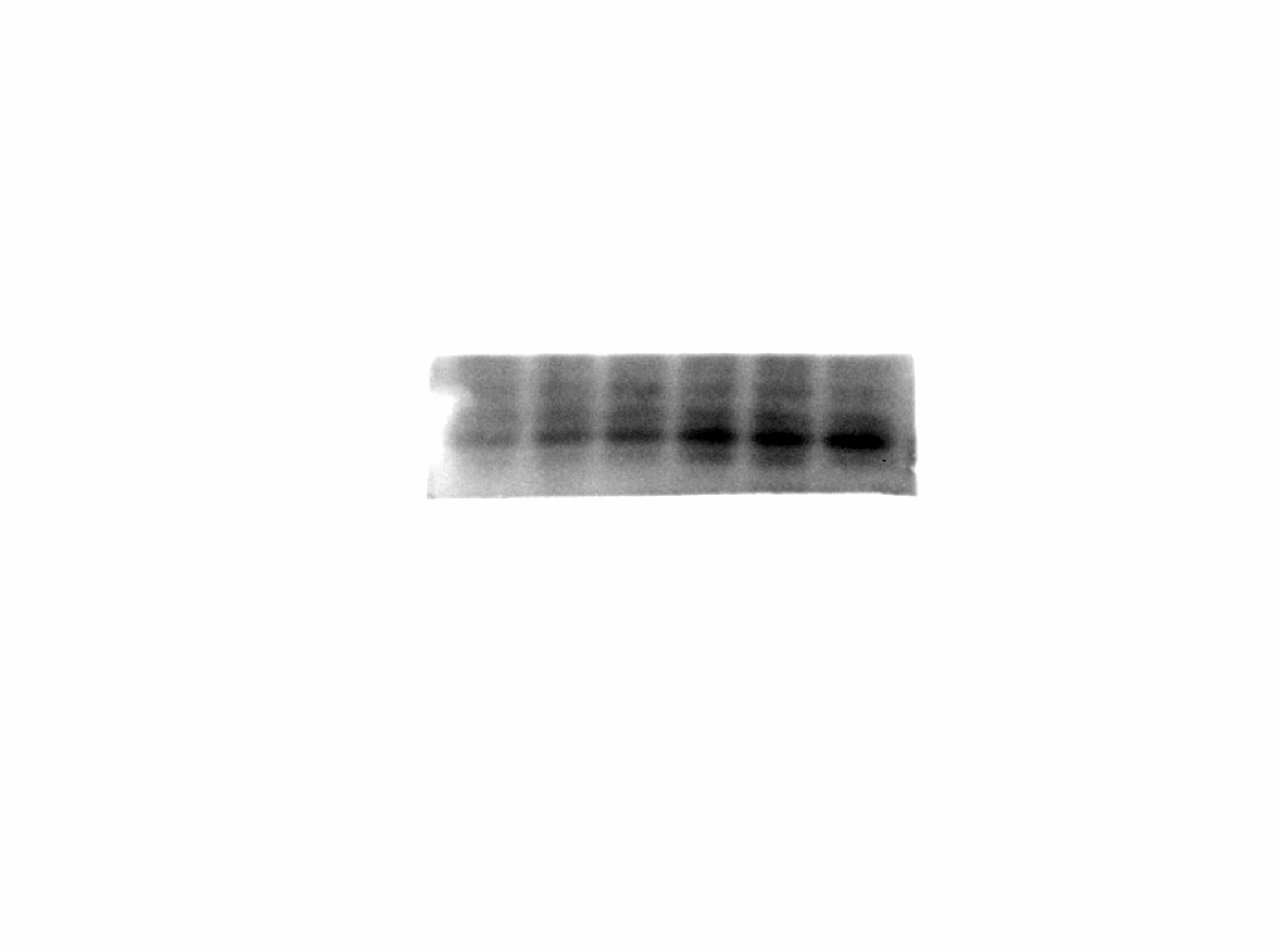

Supplement: Supplementary file 1 [file DataSheet1.ZIP › Original pictures for figures/4/4B SOCS3.jpg]

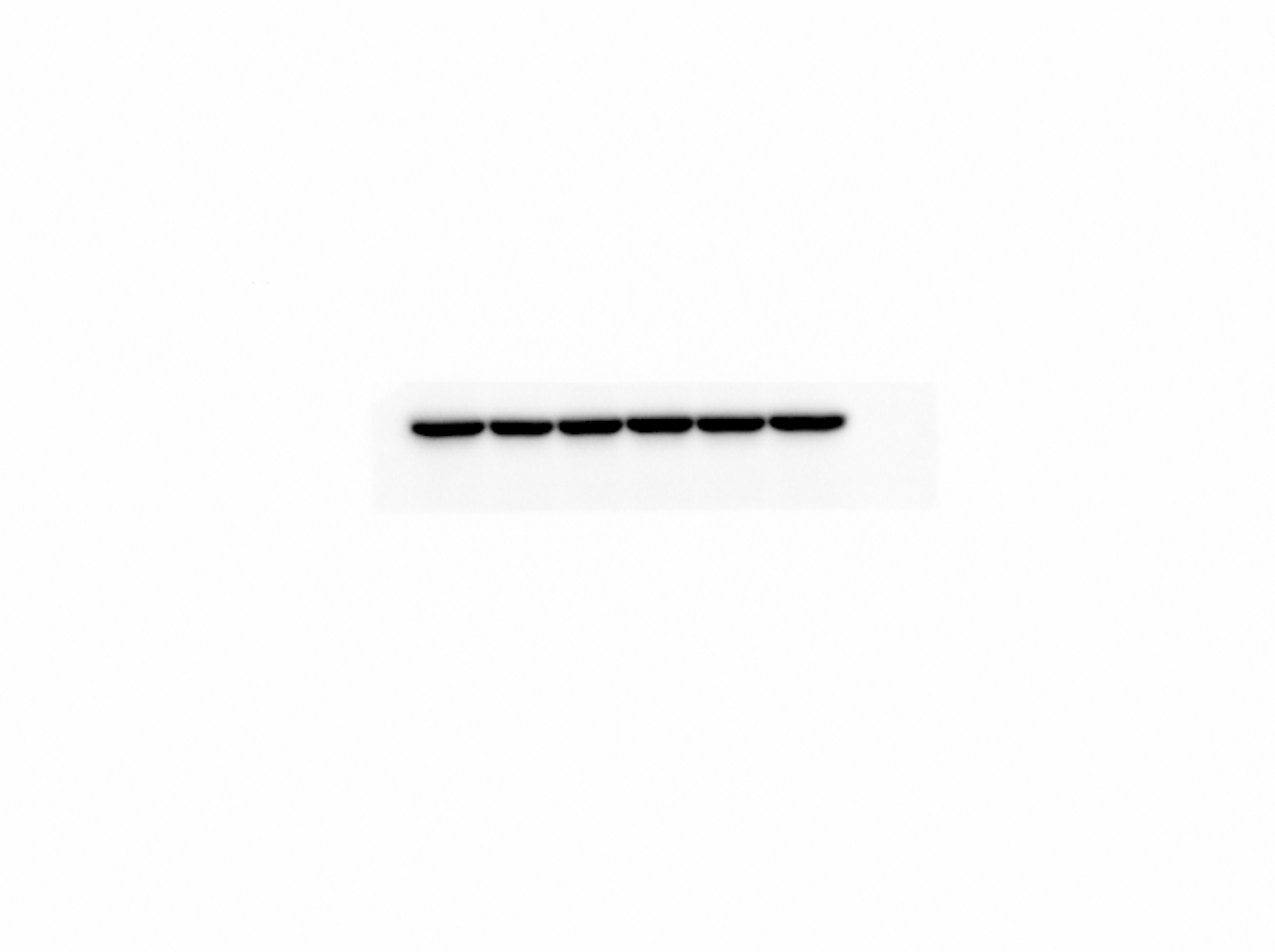

Supplement: Supplementary file 1 [file DataSheet1.ZIP › Original pictures for figures/4/4C actin.jpg]

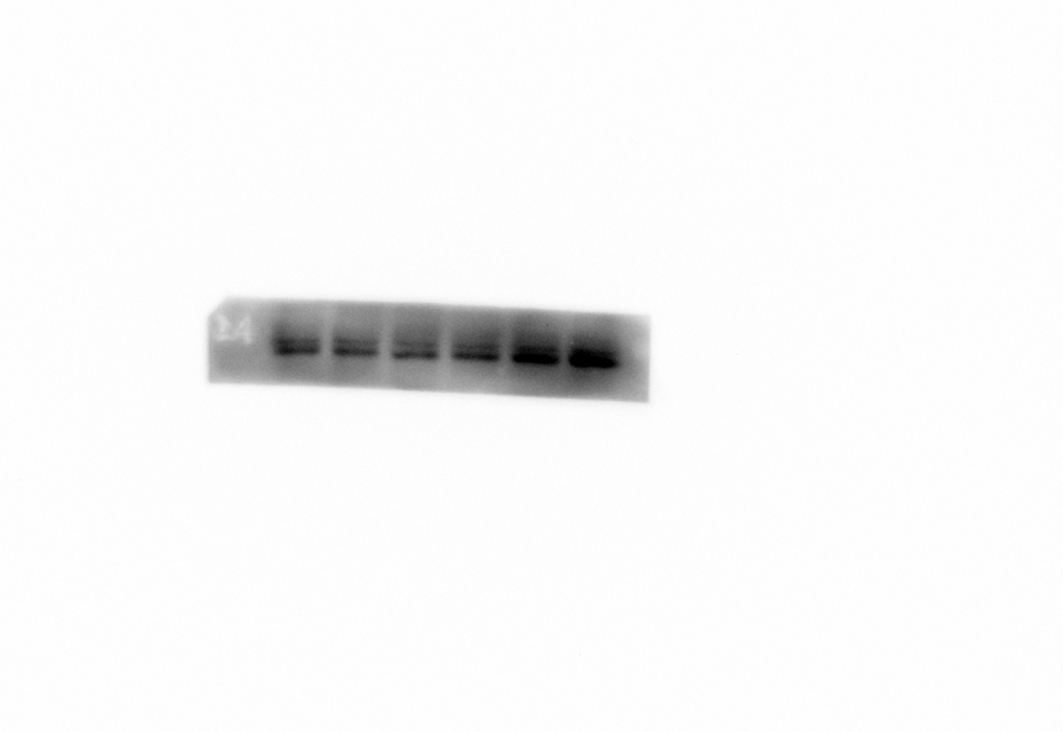

Supplement: Supplementary file 1 [file DataSheet1.ZIP › Original pictures for figures/4/4C p-AMPK.jpg]

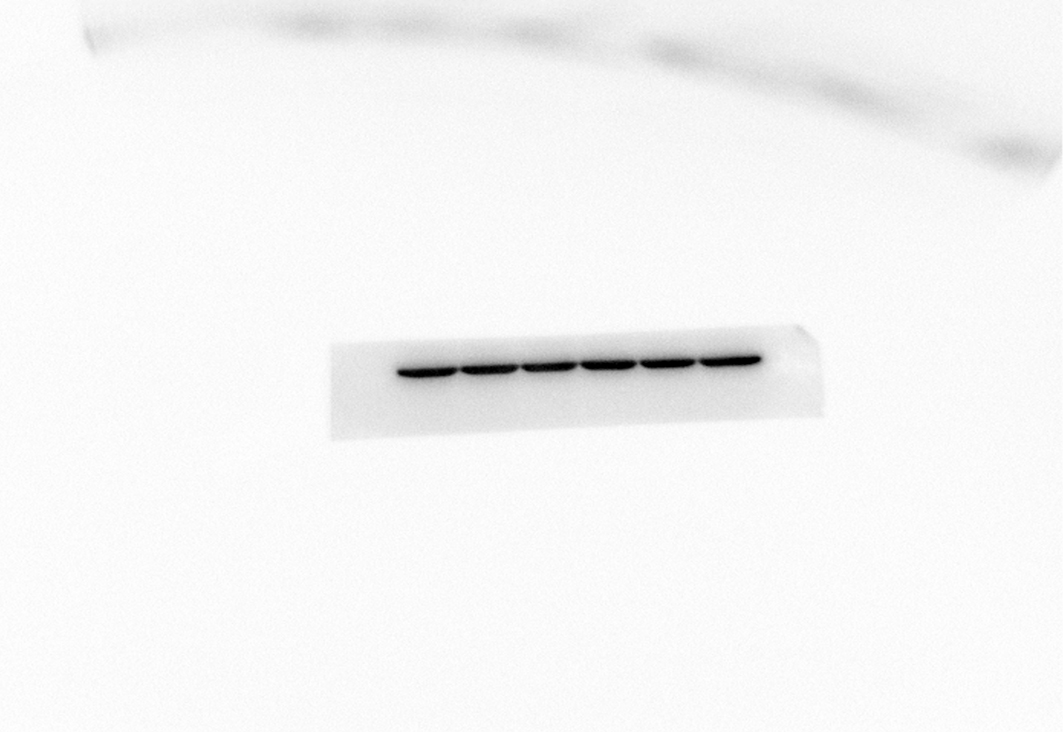

Supplement: Supplementary file 1 [file DataSheet1.ZIP › Original pictures for figures/4/4D actin.jpg]

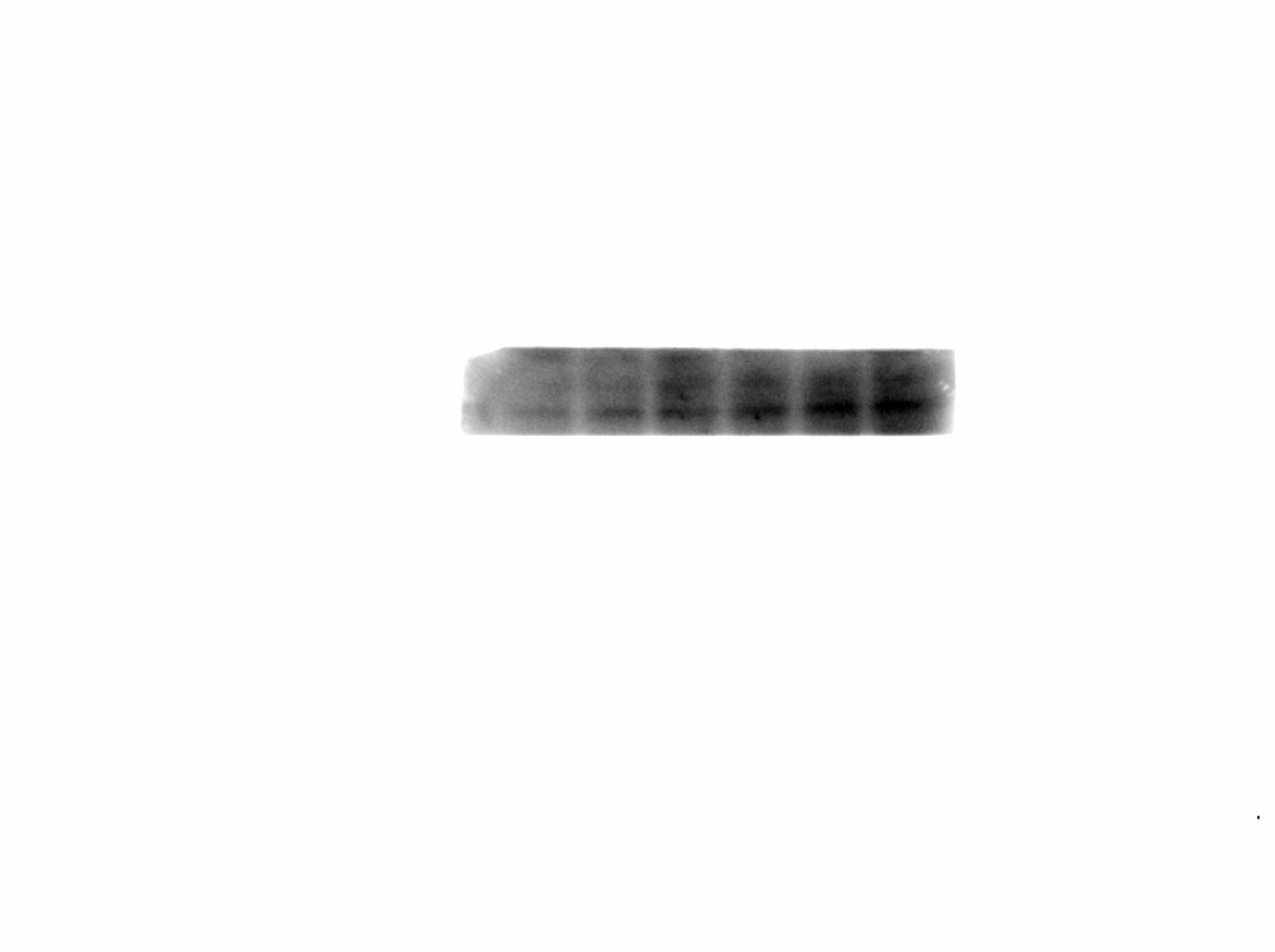

Supplement: Supplementary file 1 [file DataSheet1.ZIP › Original pictures for figures/4/4D SOCS3.jpg]

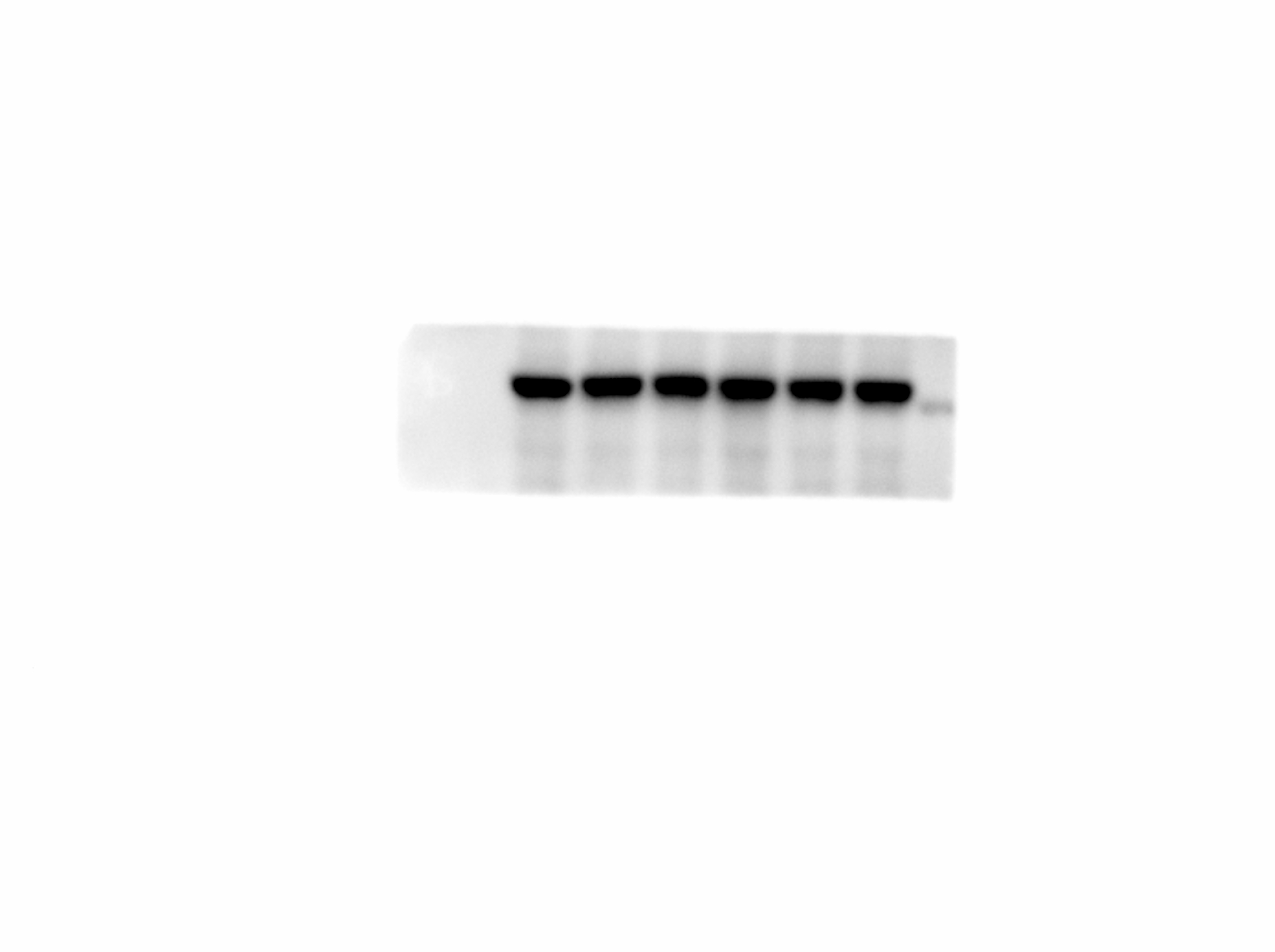

Supplement: Supplementary file 1 [file DataSheet1.ZIP › Original pictures for figures/5/5B actin.jpg]

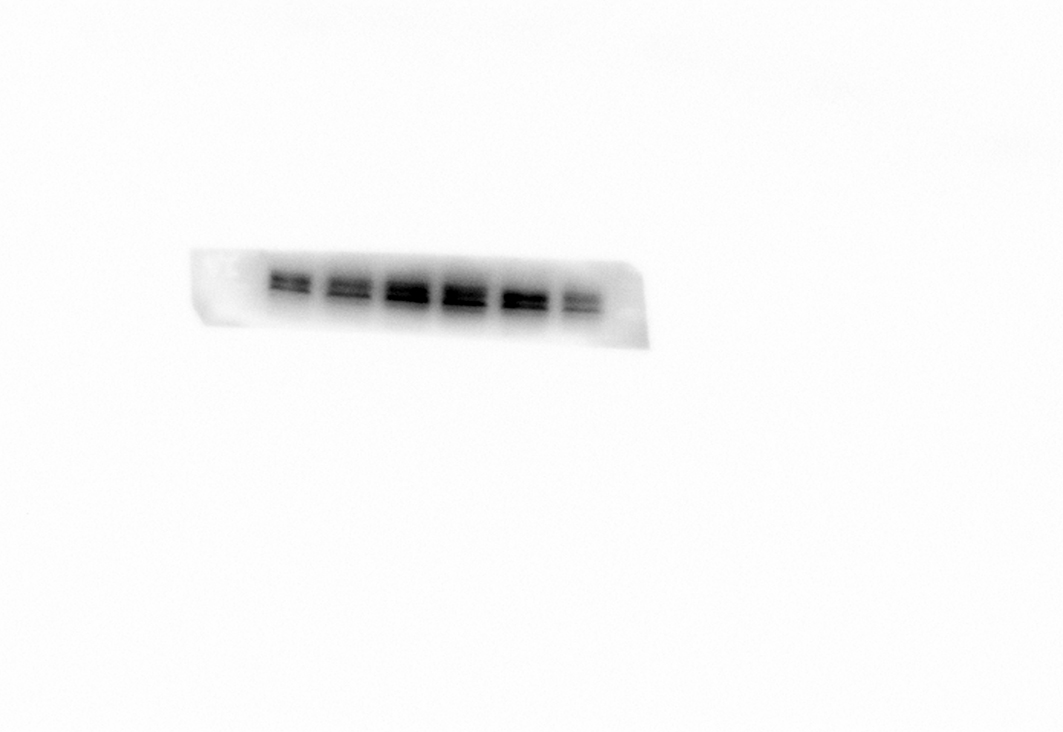

Supplement: Supplementary file 1 [file DataSheet1.ZIP › Original pictures for figures/5/5B p-AMPK.jpg]

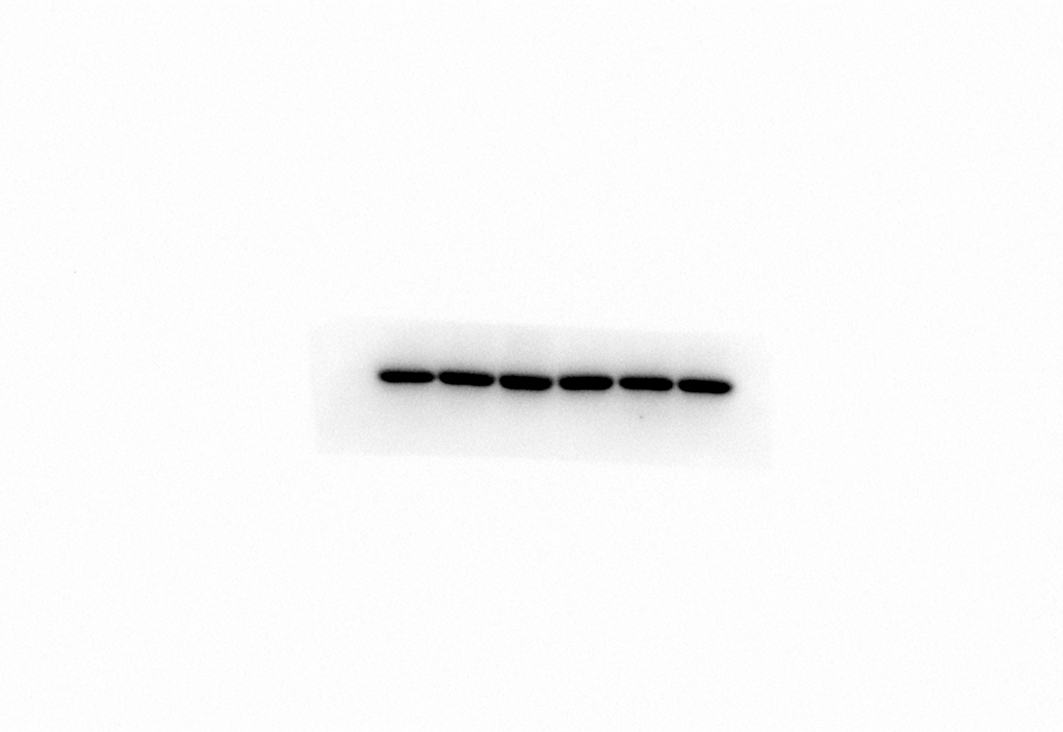

Supplement: Supplementary file 1 [file DataSheet1.ZIP › Original pictures for figures/5/5C actin.jpg]

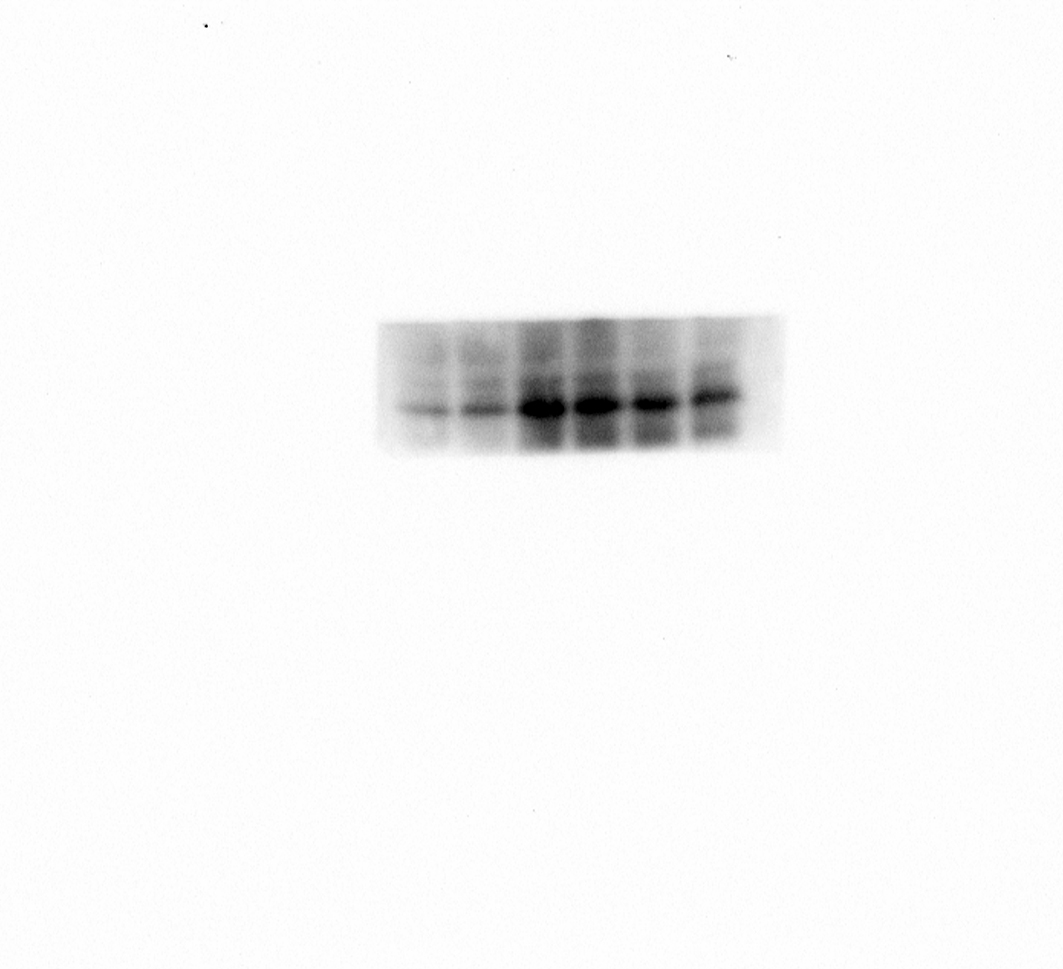

Supplement: Supplementary file 1 [file DataSheet1.ZIP › Original pictures for figures/5/5C SOCS3.jpg]

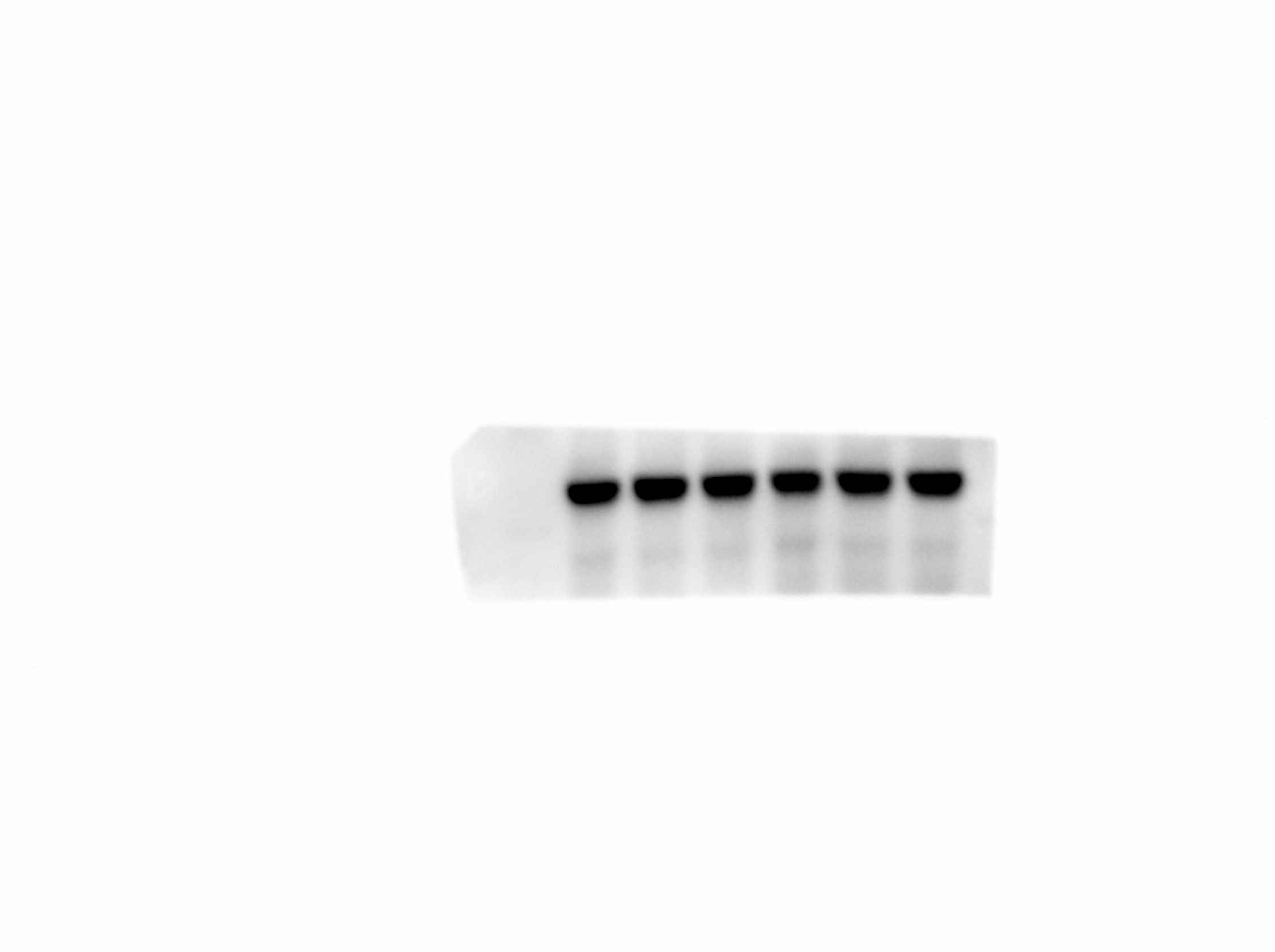

Supplement: Supplementary file 1 [file DataSheet1.ZIP › Original pictures for figures/5/5D actin.jpg]

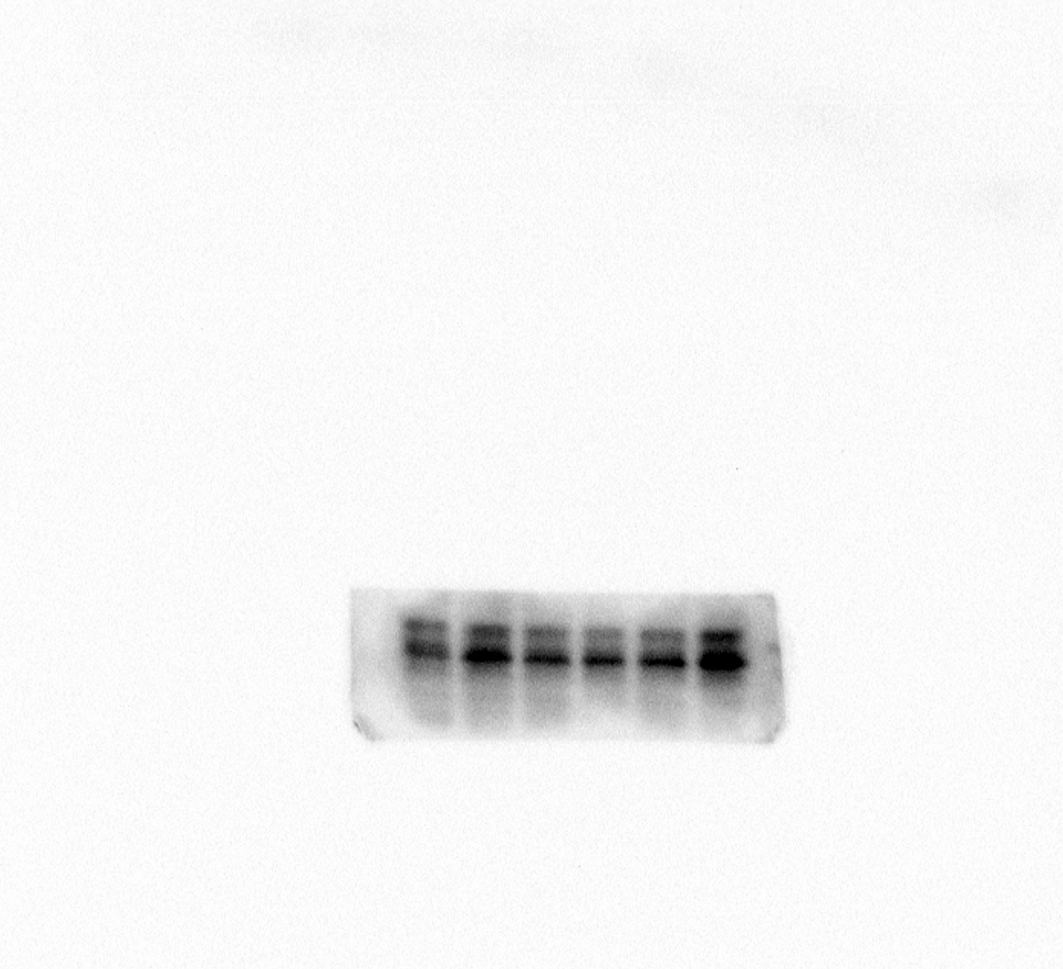

Supplement: Supplementary file 1 [file DataSheet1.ZIP › Original pictures for figures/5/5D p-JNK.jpg]

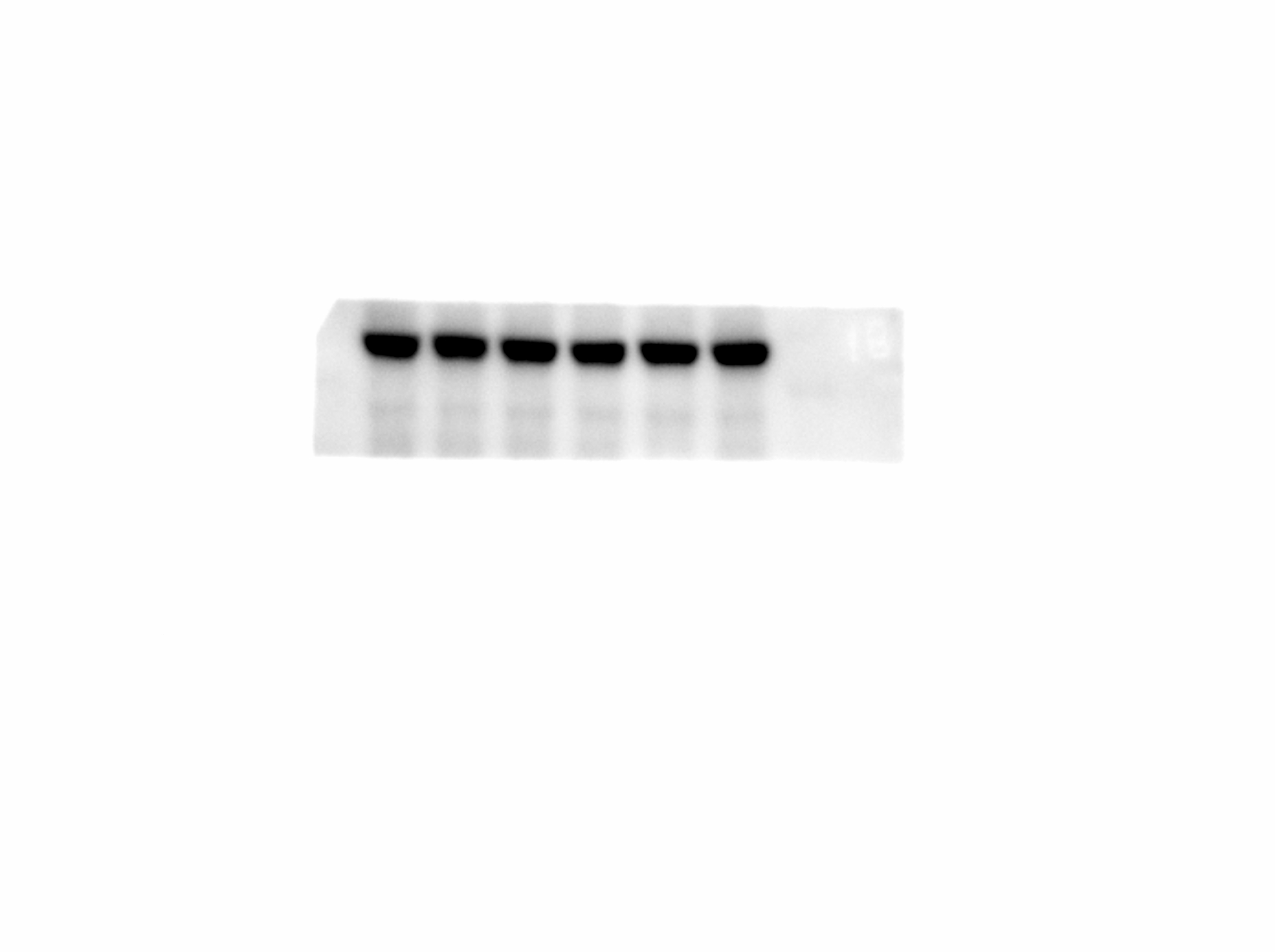

Supplement: Supplementary file 1 [file DataSheet1.ZIP › Original pictures for figures/5/5E actin.jpg]

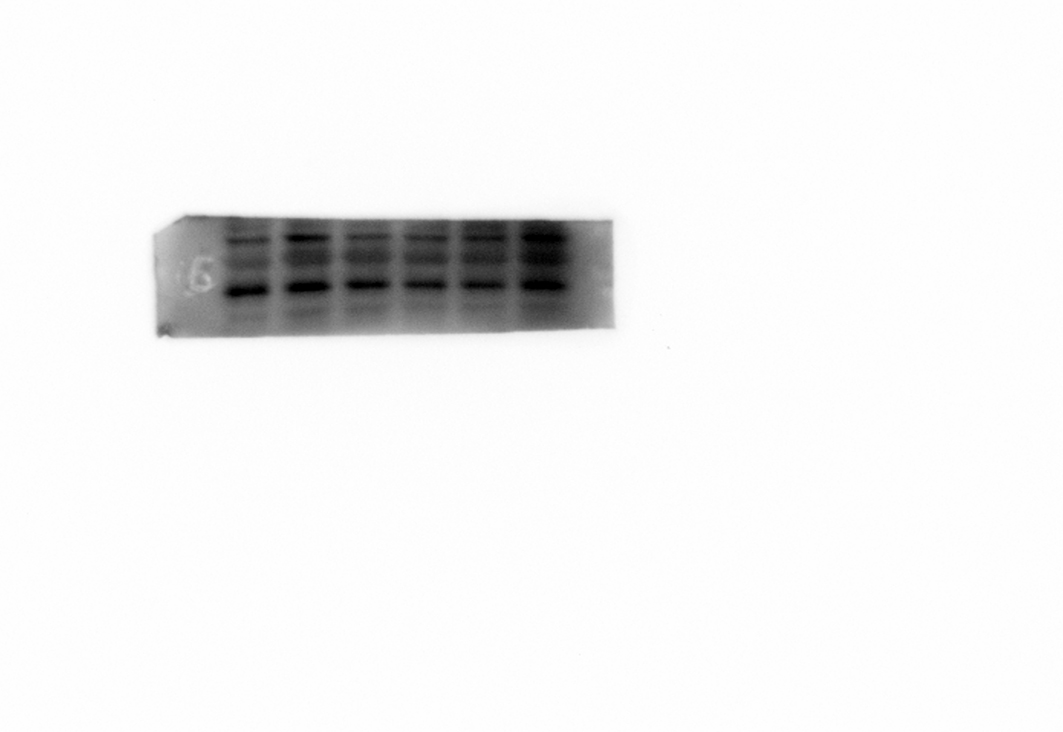

Supplement: Supplementary file 1 [file DataSheet1.ZIP › Original pictures for figures/5/5E p-Cx43.jpg]

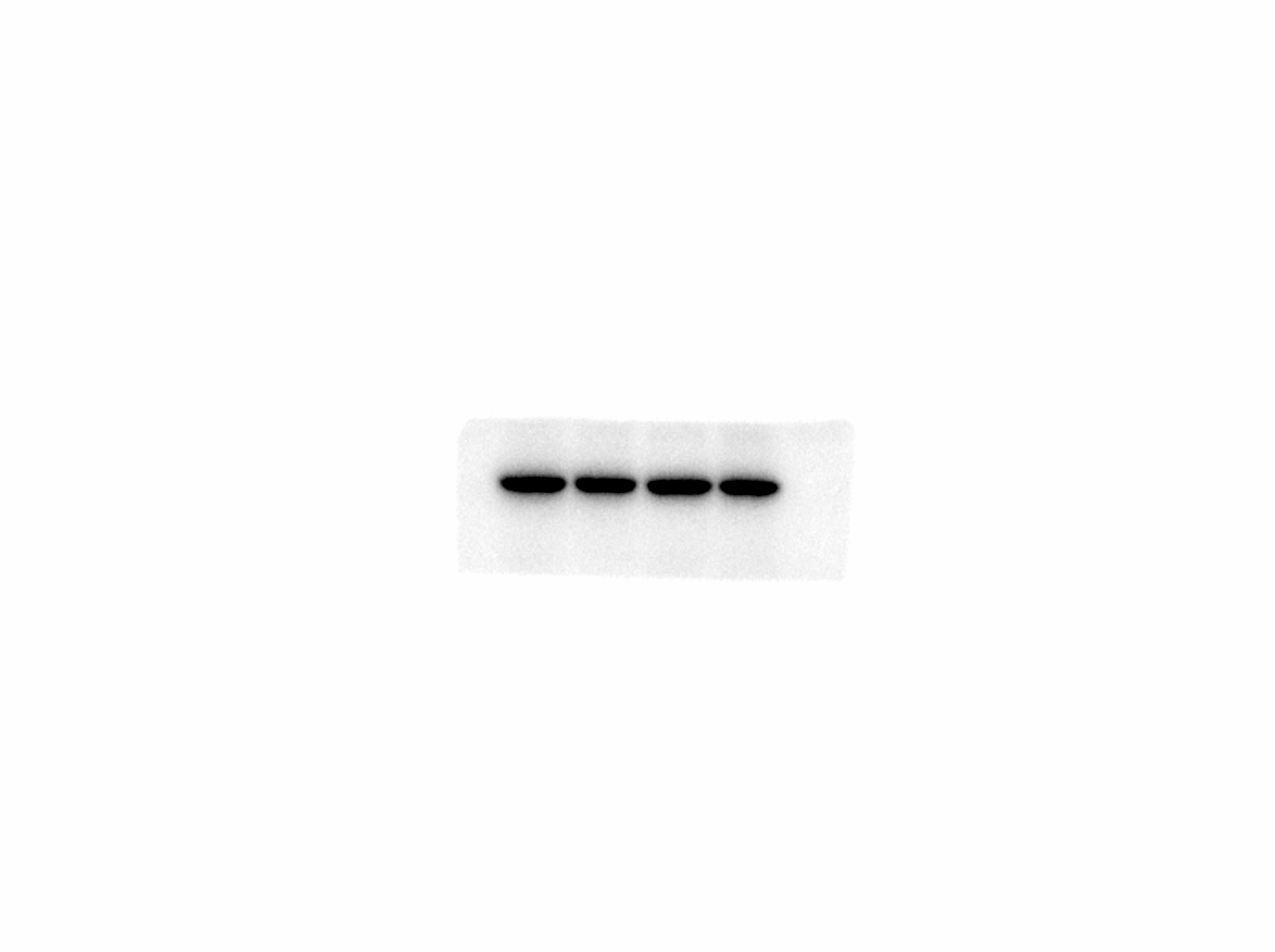

Supplement: Supplementary file 1 [file DataSheet1.ZIP › Original pictures for figures/6/6A actin.jpg]

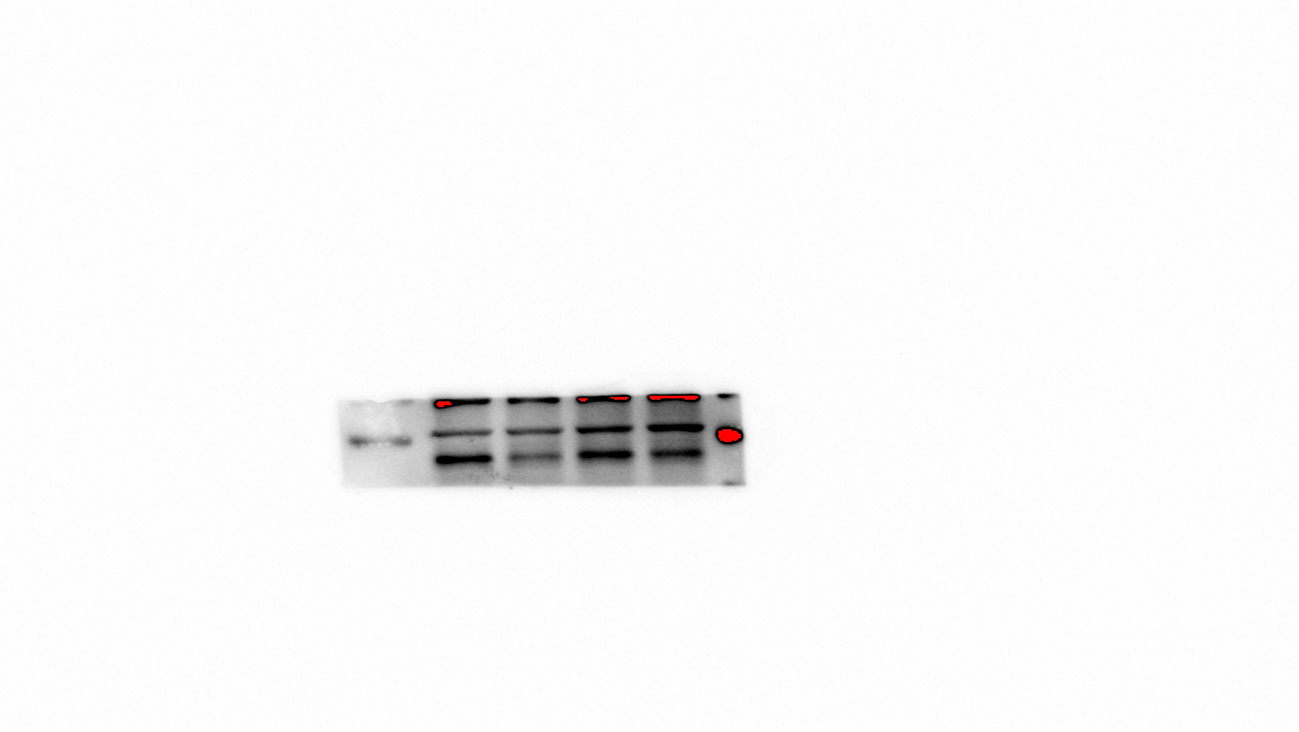

Supplement: Supplementary file 1 [file DataSheet1.ZIP › Original pictures for figures/6/6A p-AMPK.jpg]

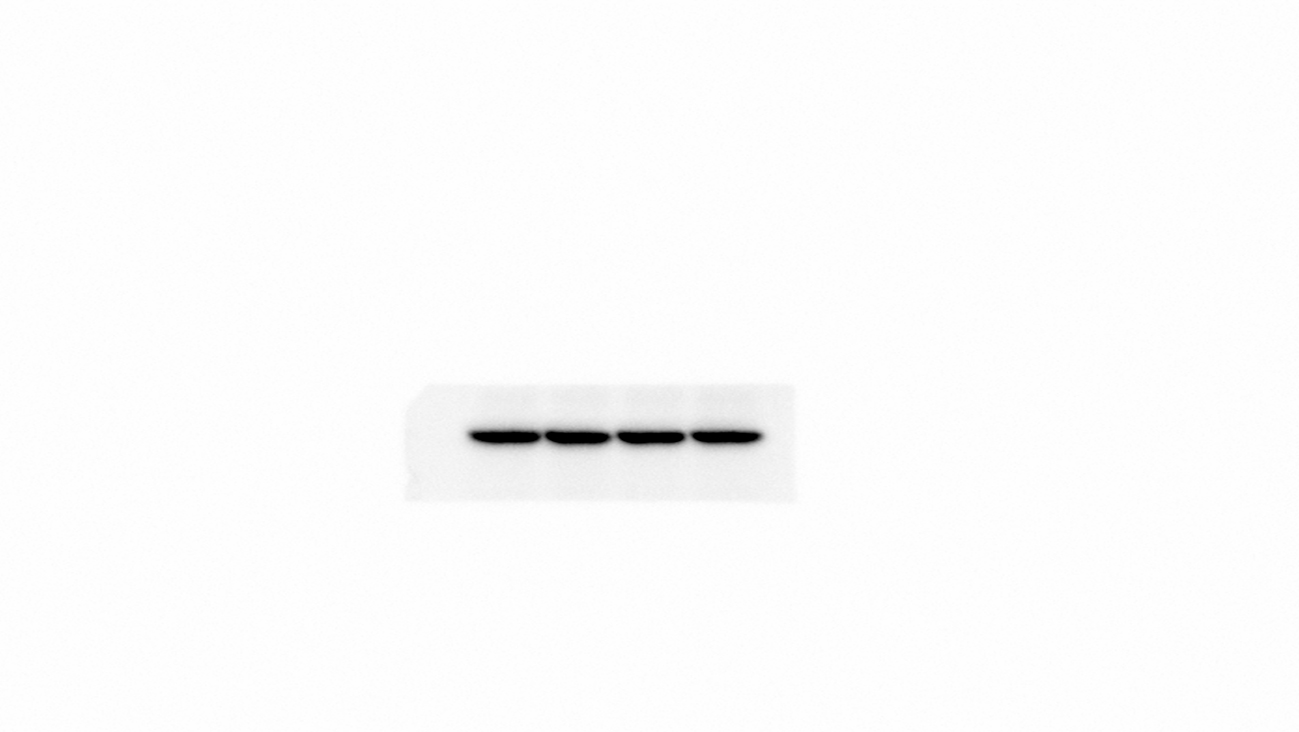

Supplement: Supplementary file 1 [file DataSheet1.ZIP › Original pictures for figures/6/6B actin.jpg]

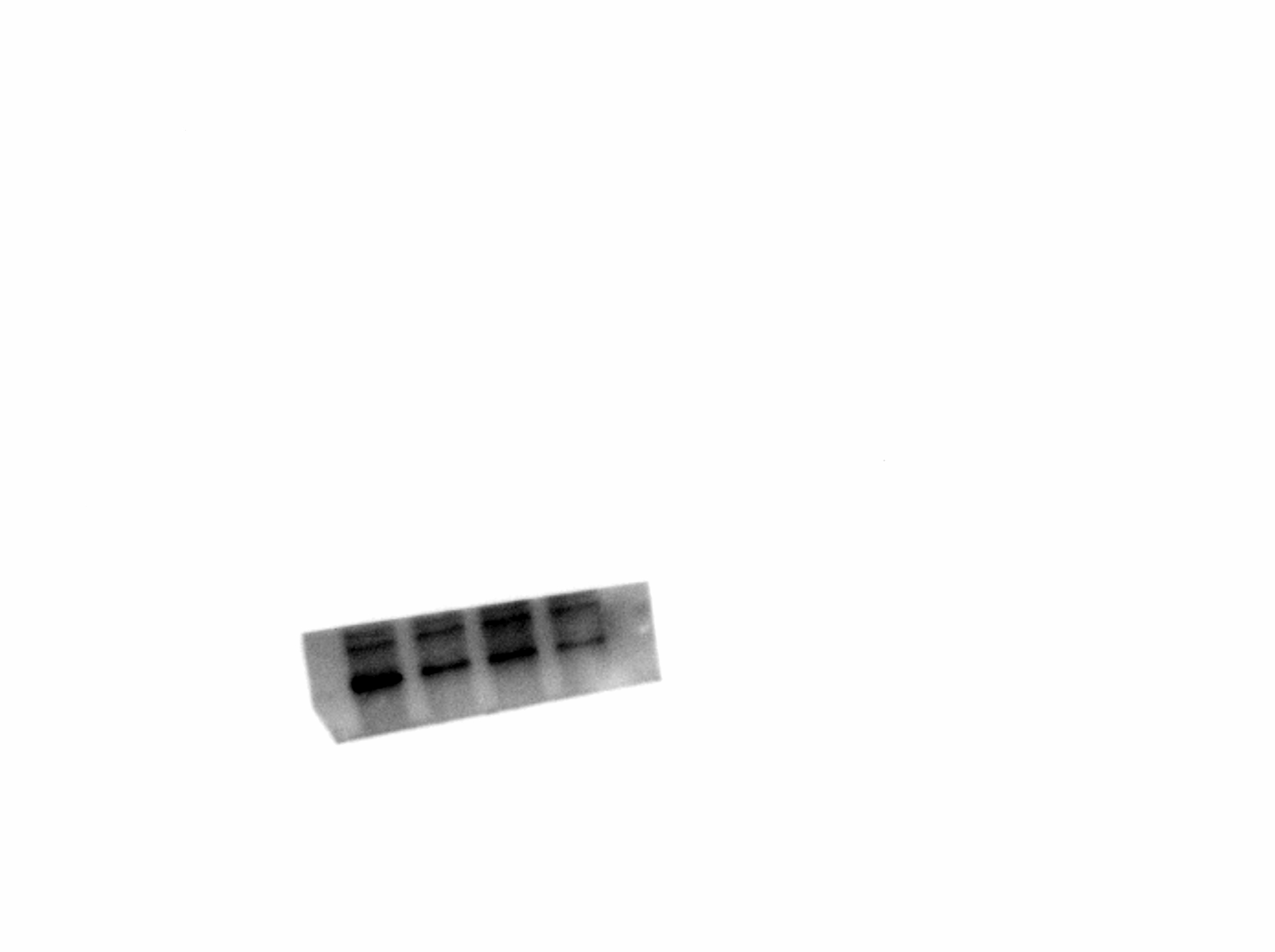

Supplement: Supplementary file 1 [file DataSheet1.ZIP › Original pictures for figures/6/6B Axl.jpg]

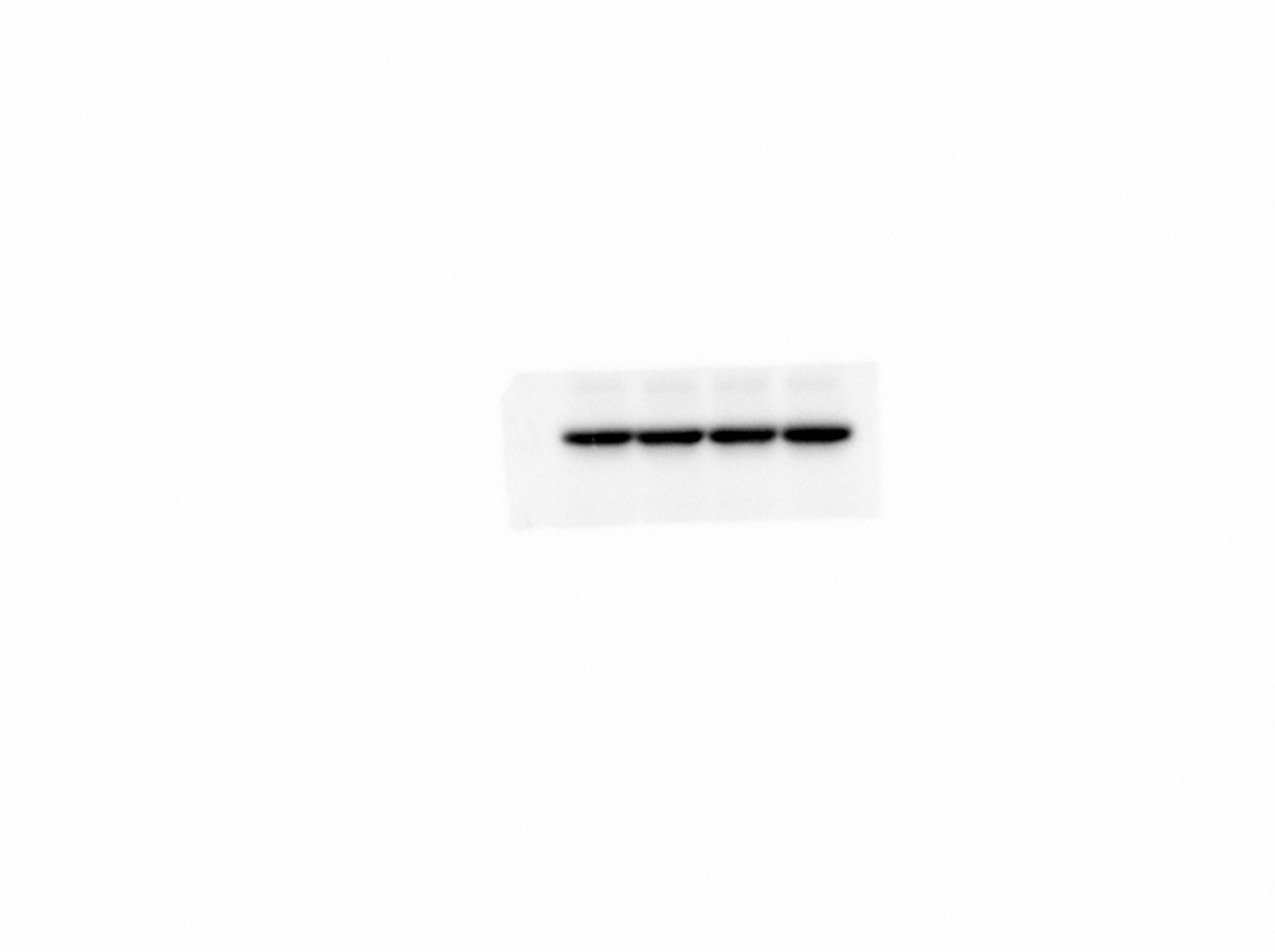

Supplement: Supplementary file 1 [file DataSheet1.ZIP › Original pictures for figures/6/6C actin.jpg]

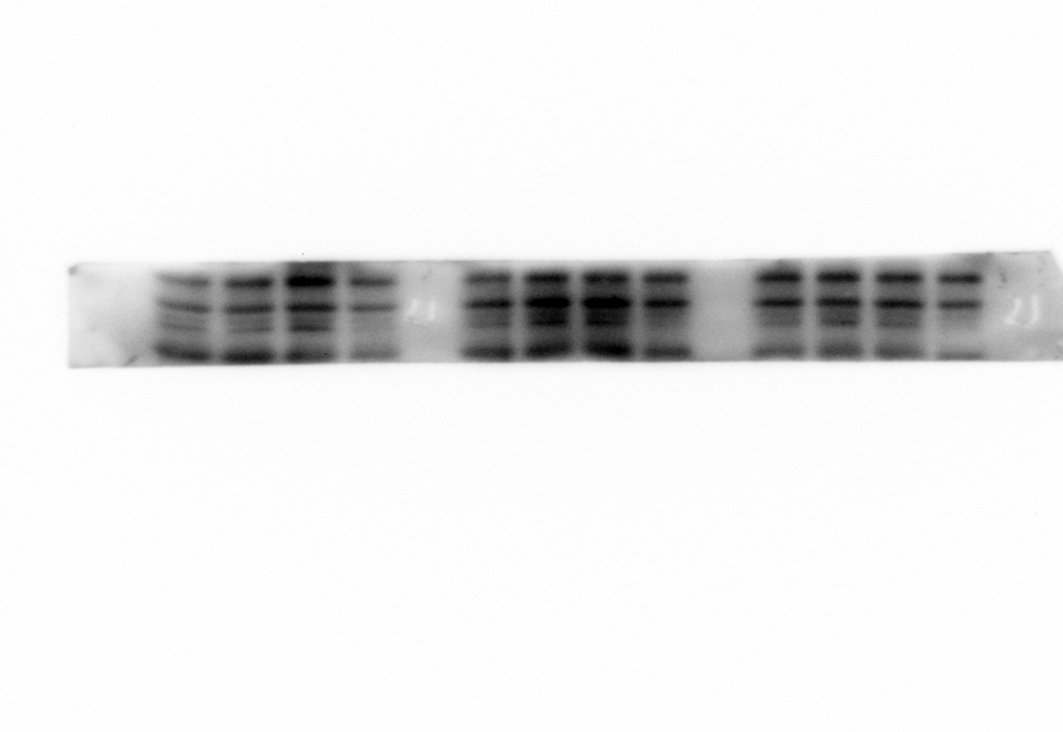

Supplement: Supplementary file 1 [file DataSheet1.ZIP › Original pictures for figures/6/6C SOCS3.jpg]

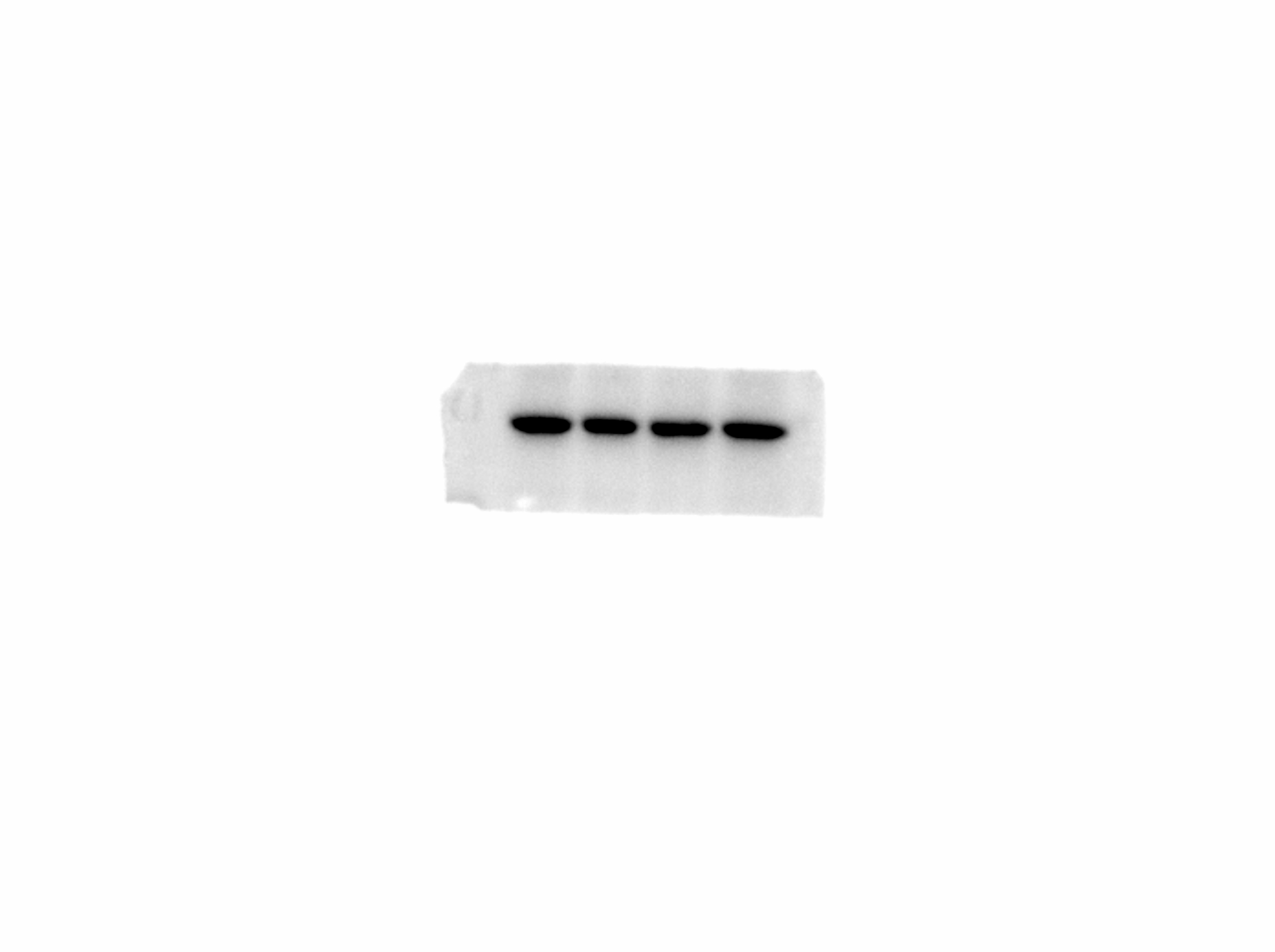

Supplement: Supplementary file 1 [file DataSheet1.ZIP › Original pictures for figures/6/6D actin.jpg]

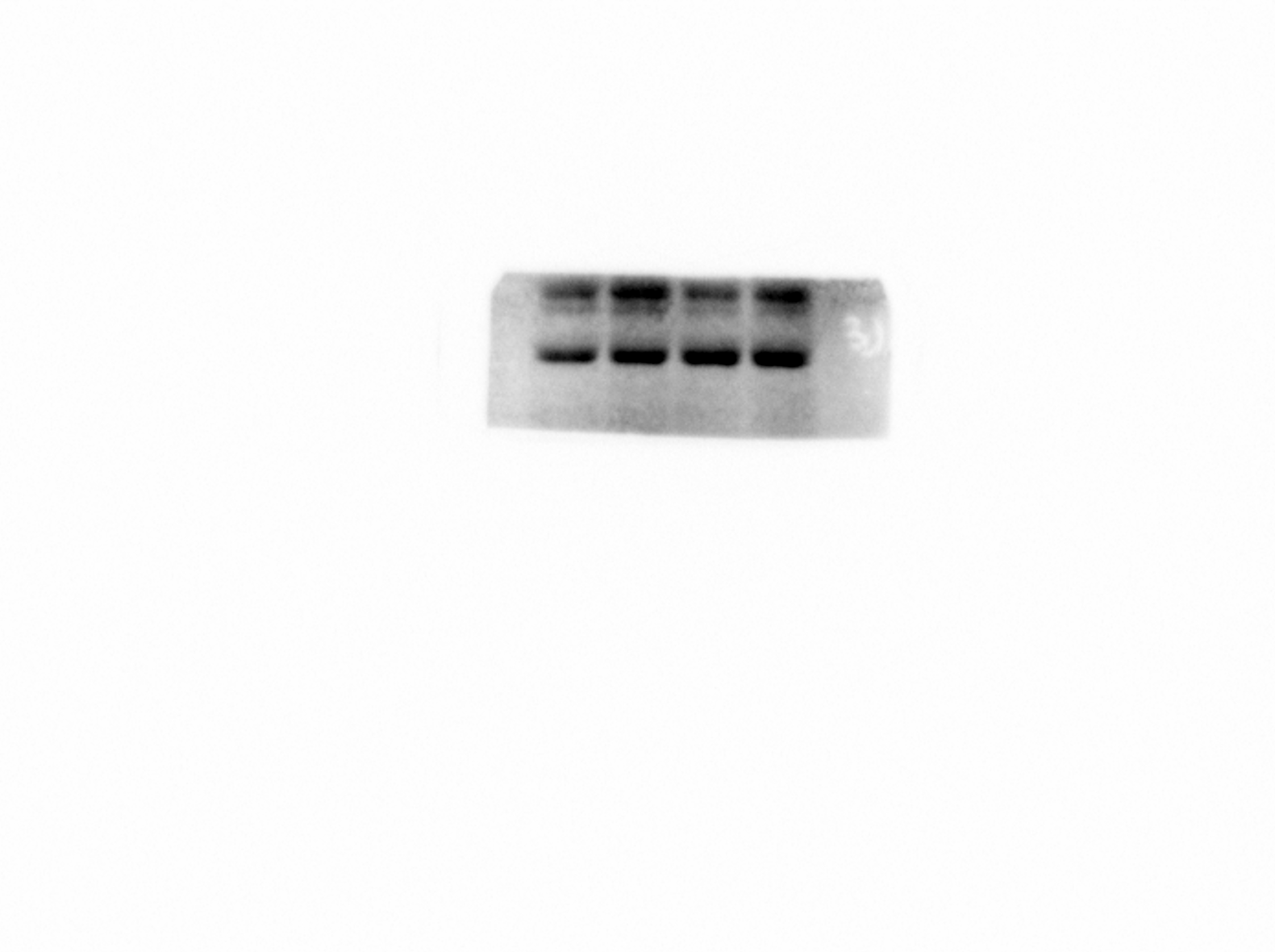

Supplement: Supplementary file 1 [file DataSheet1.ZIP › Original pictures for figures/6/6D p-JNK.jpg]

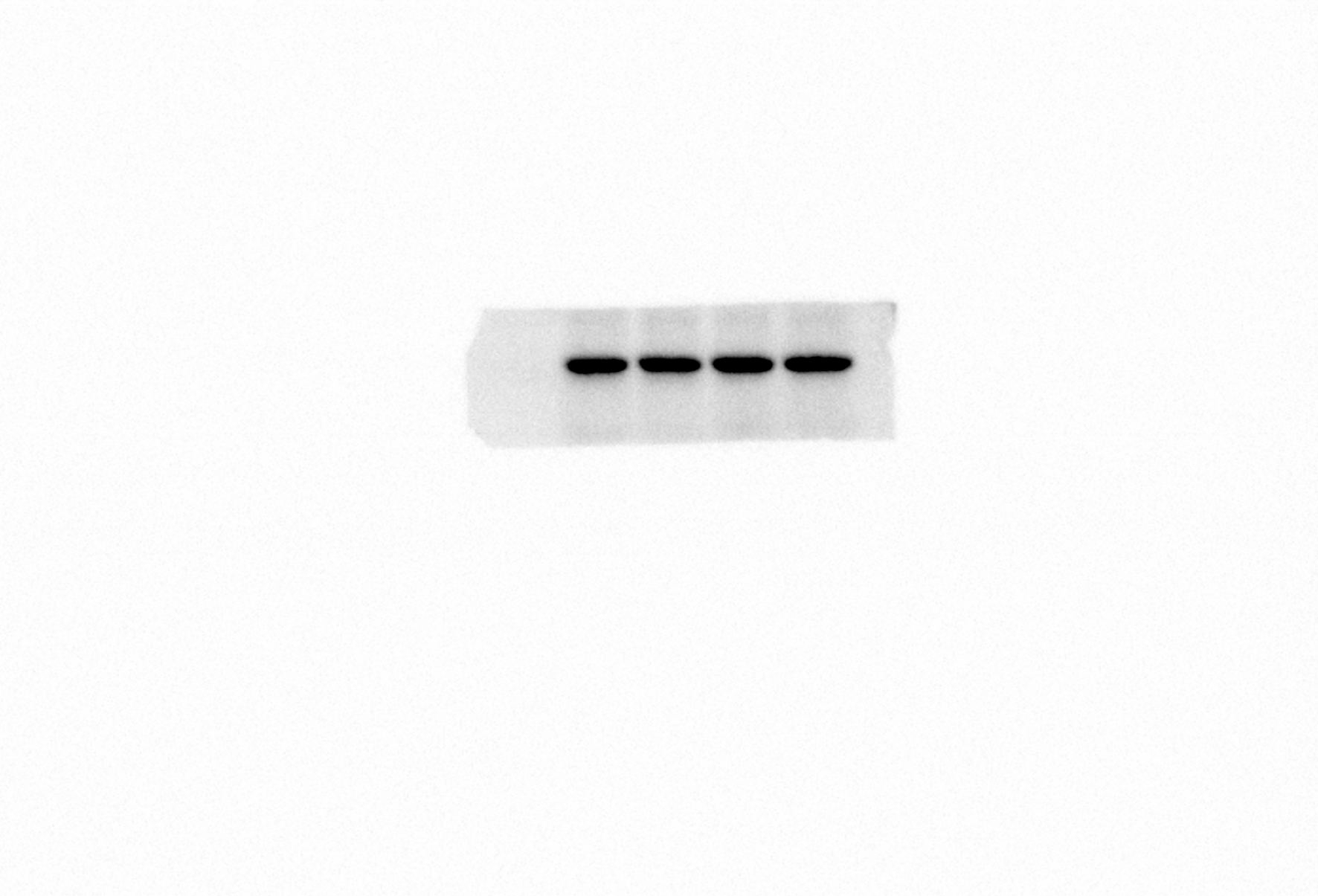

Supplement: Supplementary file 1 [file DataSheet1.ZIP › Original pictures for figures/6/6E actin.jpg]

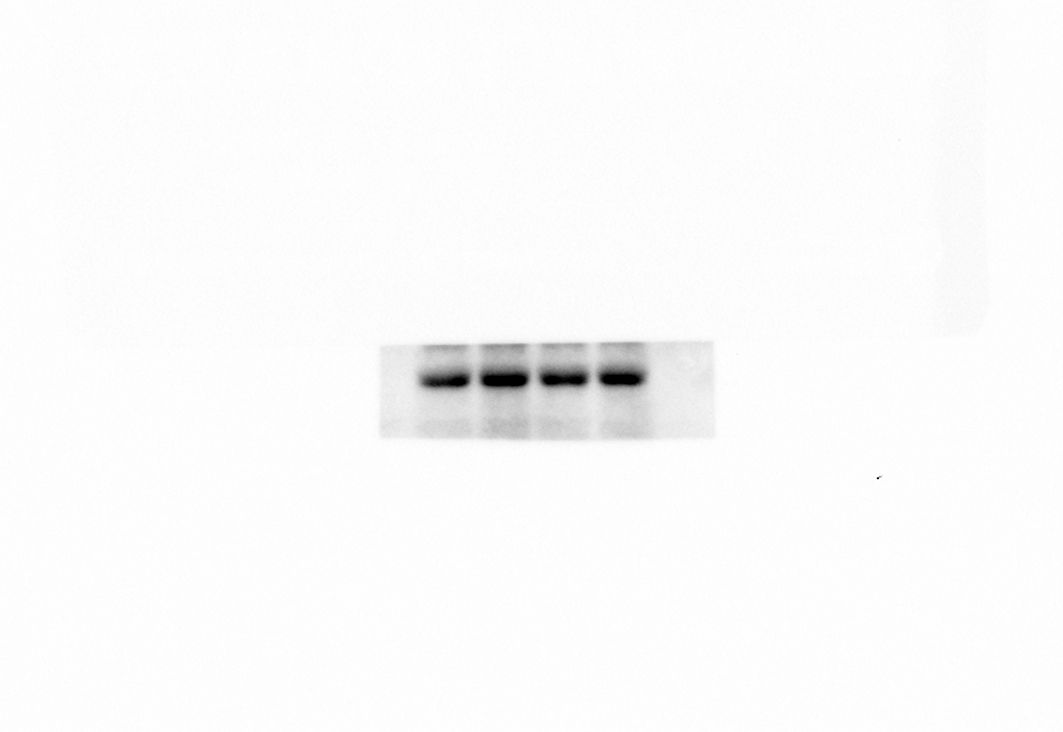

Supplement: Supplementary file 1 [file DataSheet1.ZIP › Original pictures for figures/6/6E p-Cx43.jpg]

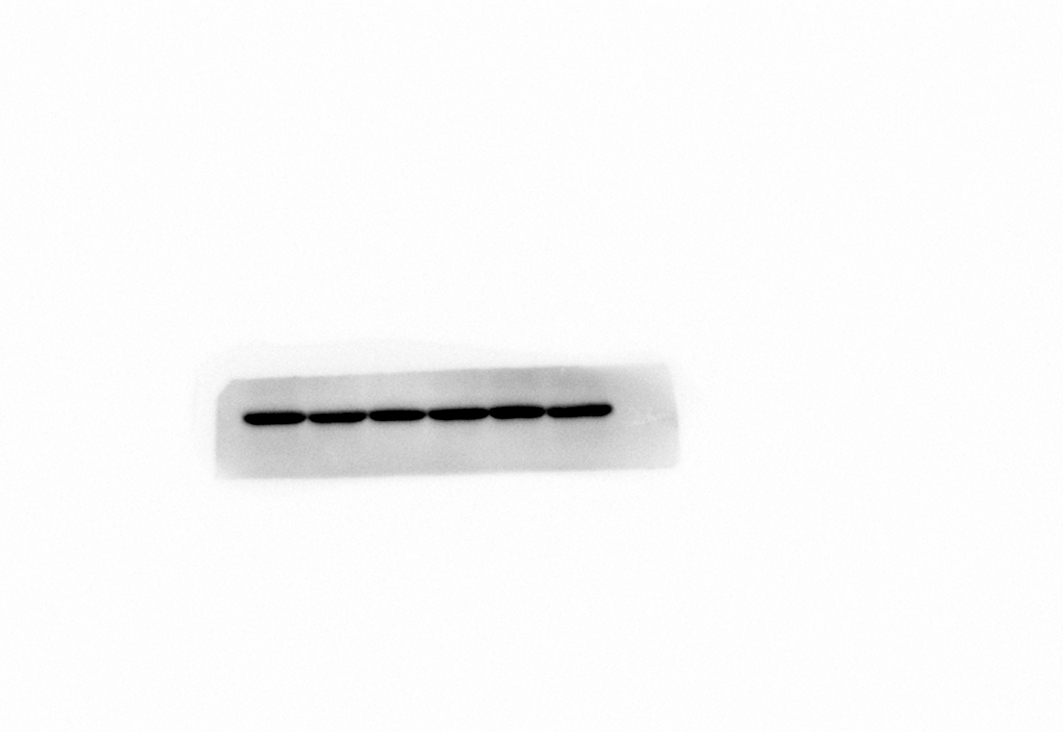

Supplement: Supplementary file 1 [file DataSheet1.ZIP › Original pictures for figures/6/6F actin.jpg]

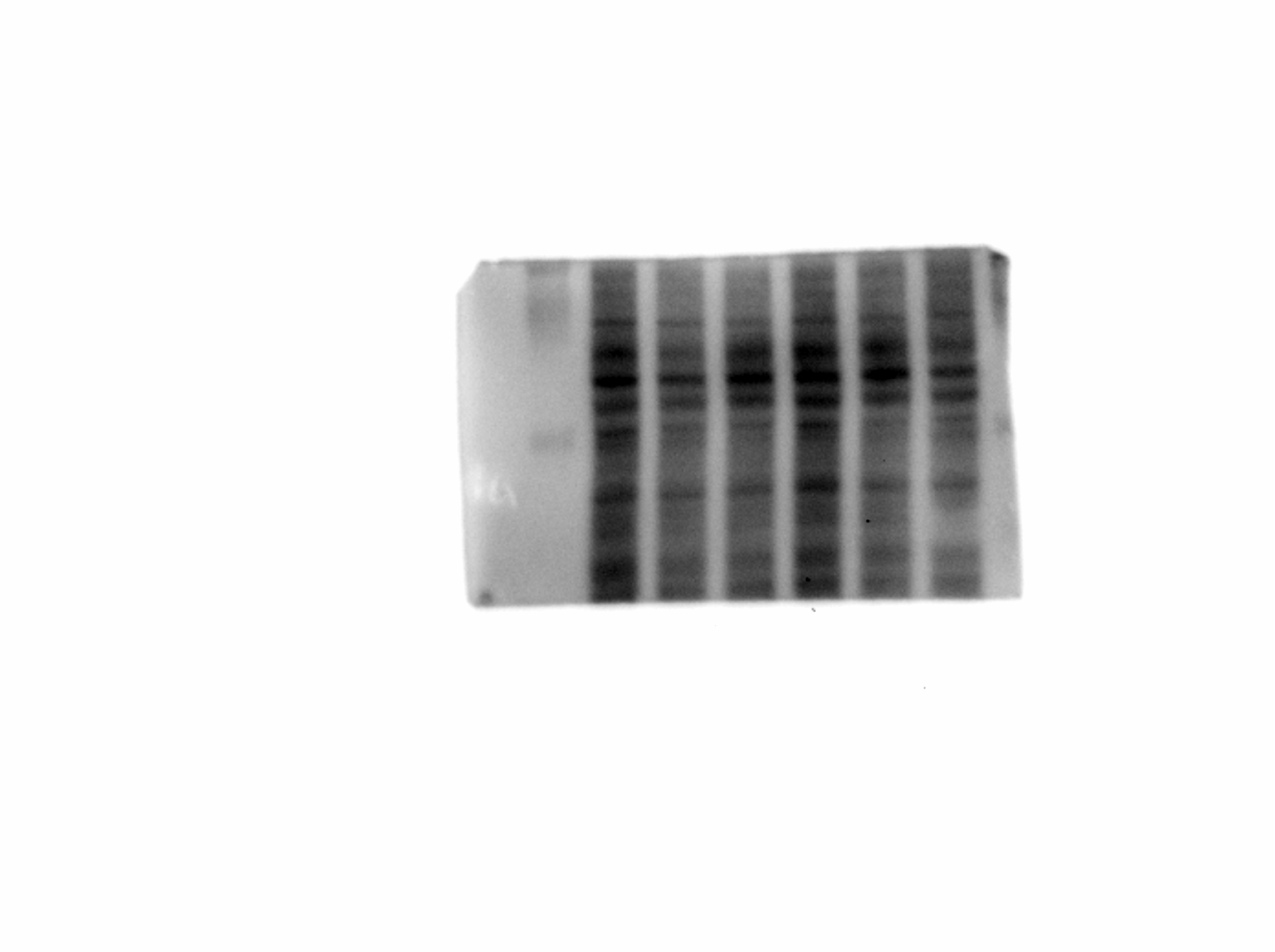

Supplement: Supplementary file 1 [file DataSheet1.ZIP › Original pictures for figures/6/6F Axl.jpg]

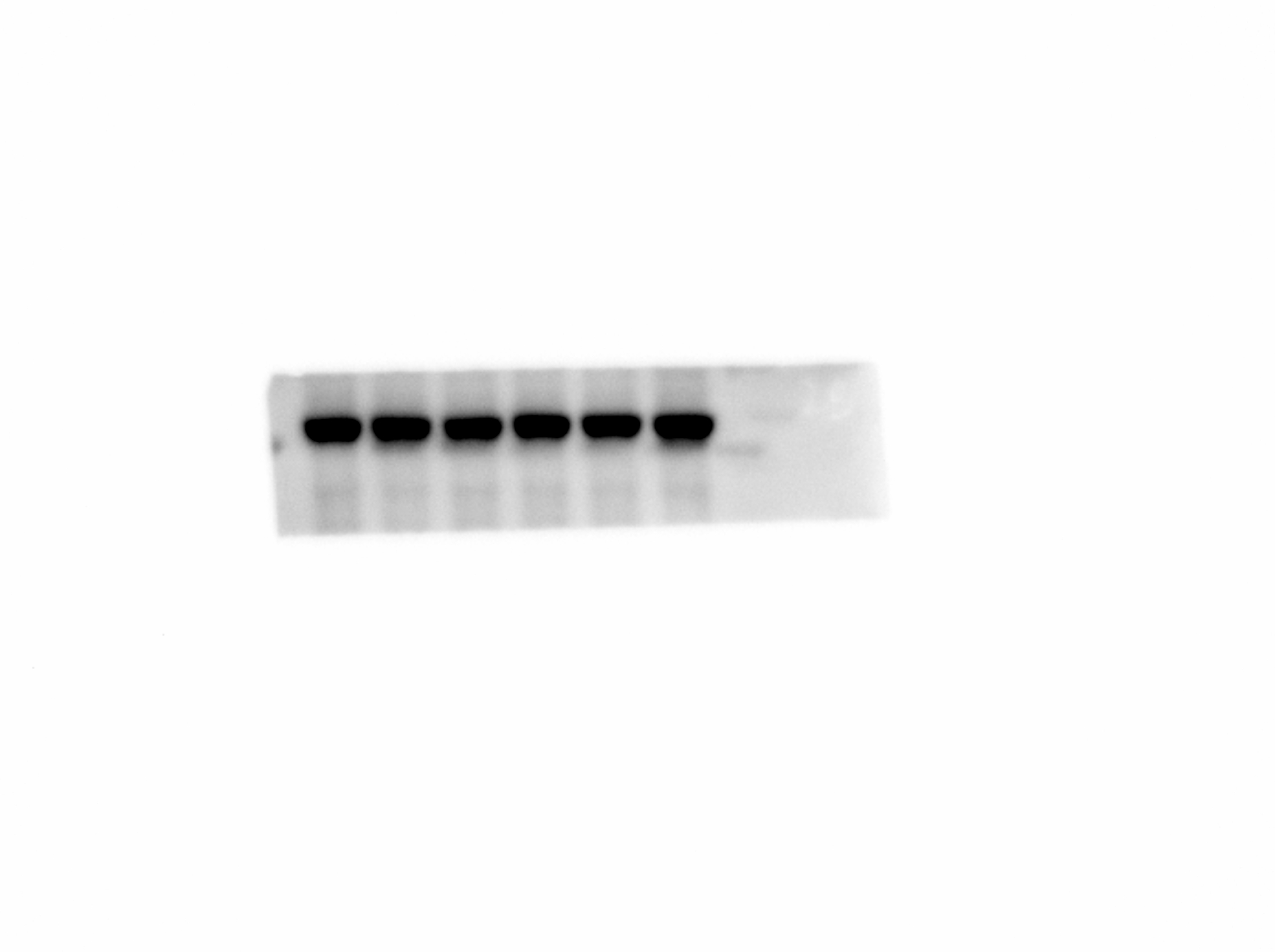

Supplement: Supplementary file 1 [file DataSheet1.ZIP › Original pictures for figures/6/6G actin.jpg]

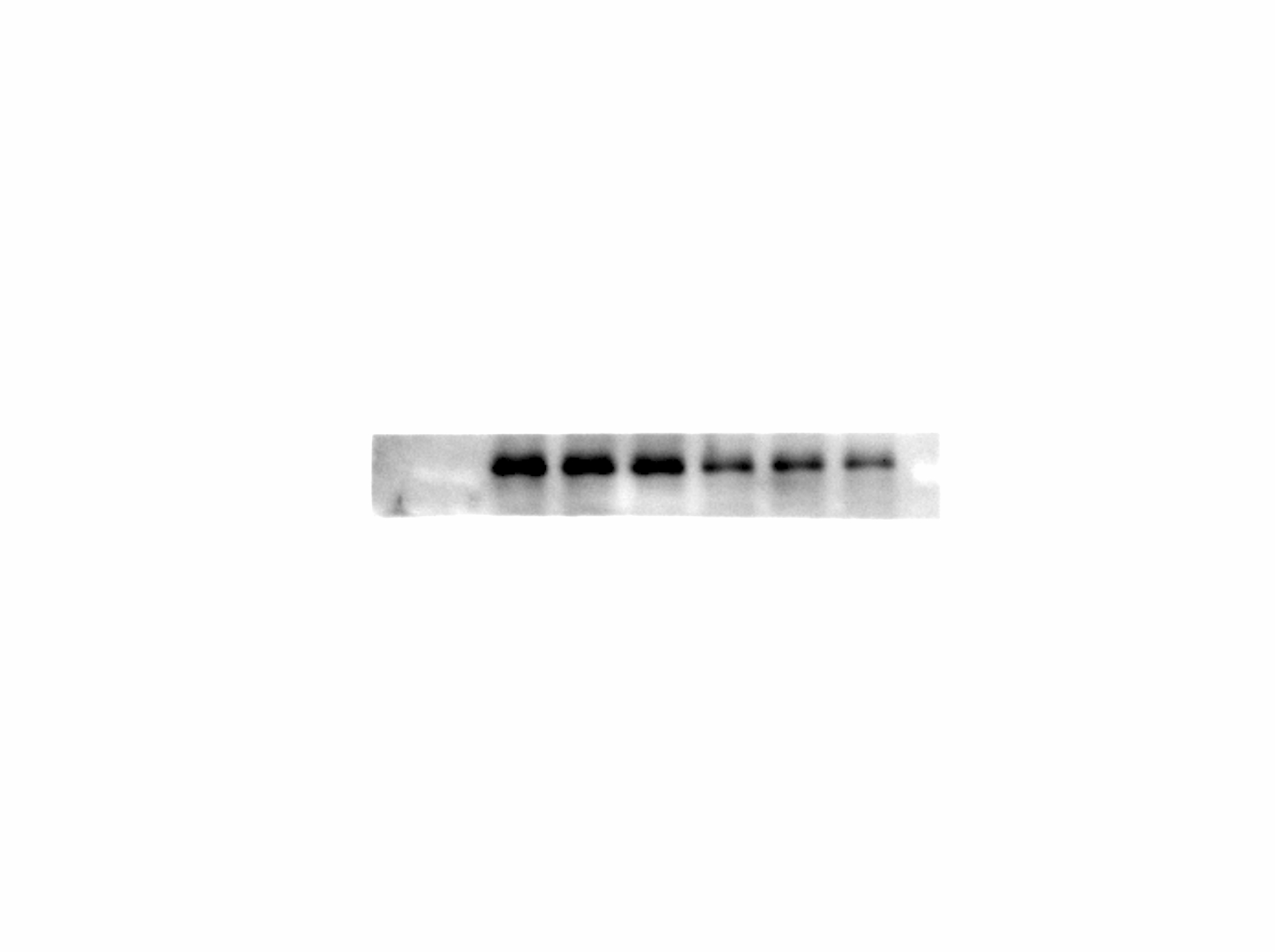

Supplement: Supplementary file 1 [file DataSheet1.ZIP › Original pictures for figures/6/6G Axl.jpg]

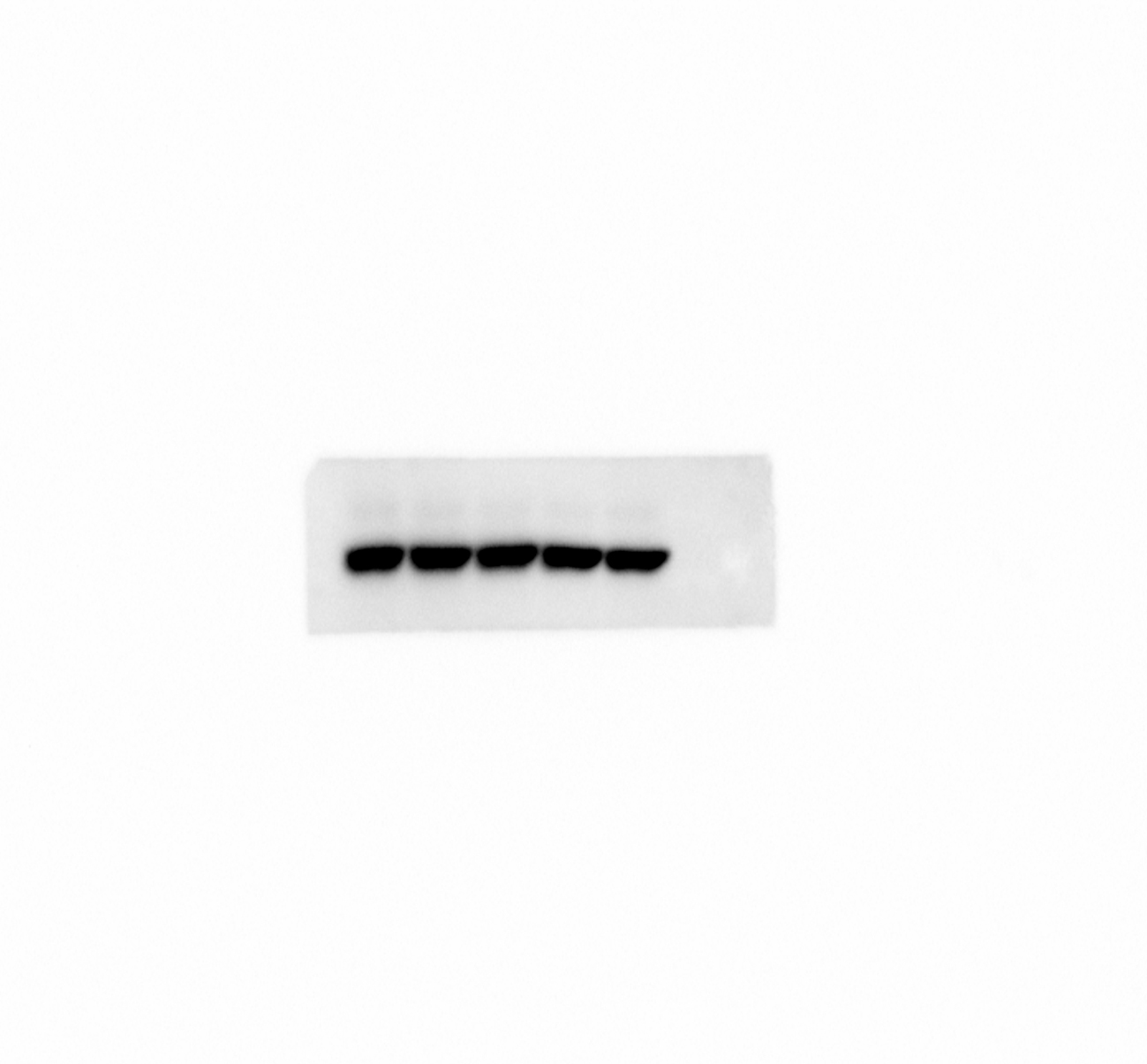

Supplement: Supplementary file 1 [file DataSheet1.ZIP › Original pictures for figures/6/6H actin.jpg]

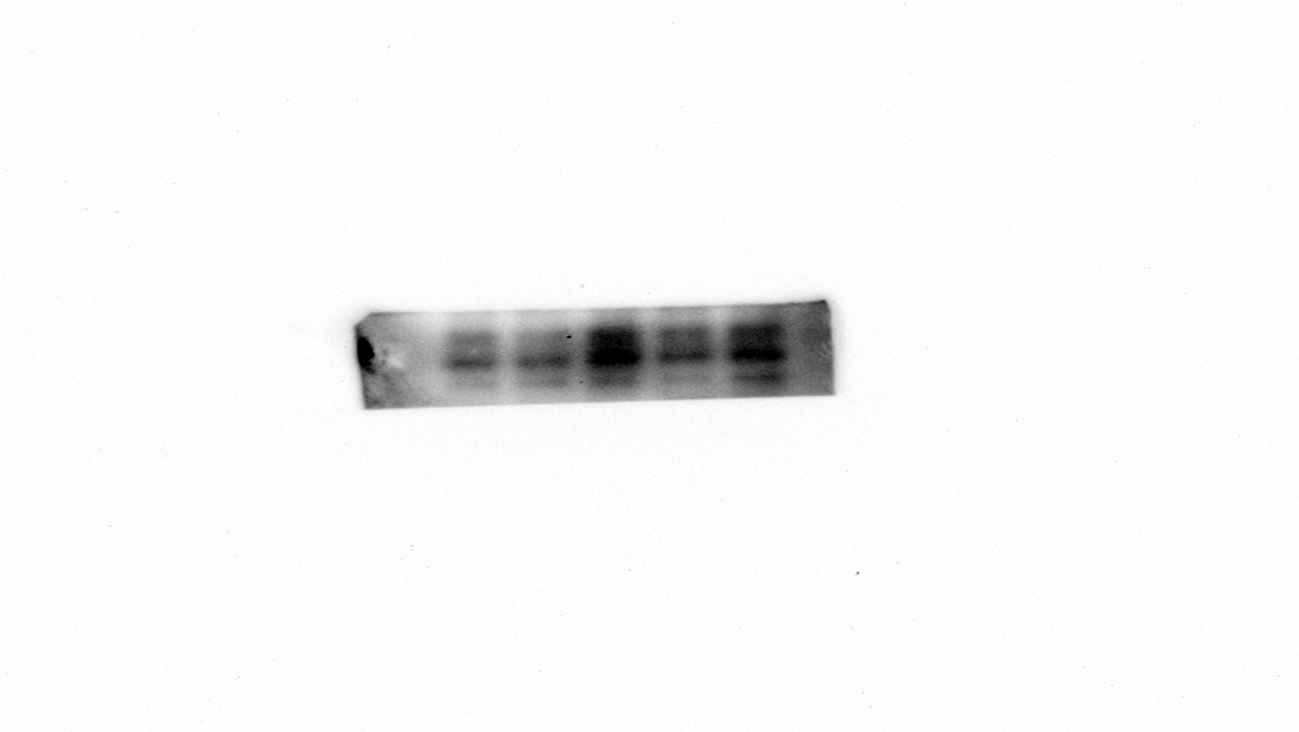

Supplement: Supplementary file 1 [file DataSheet1.ZIP › Original pictures for figures/6/6H SOCS3.jpg]

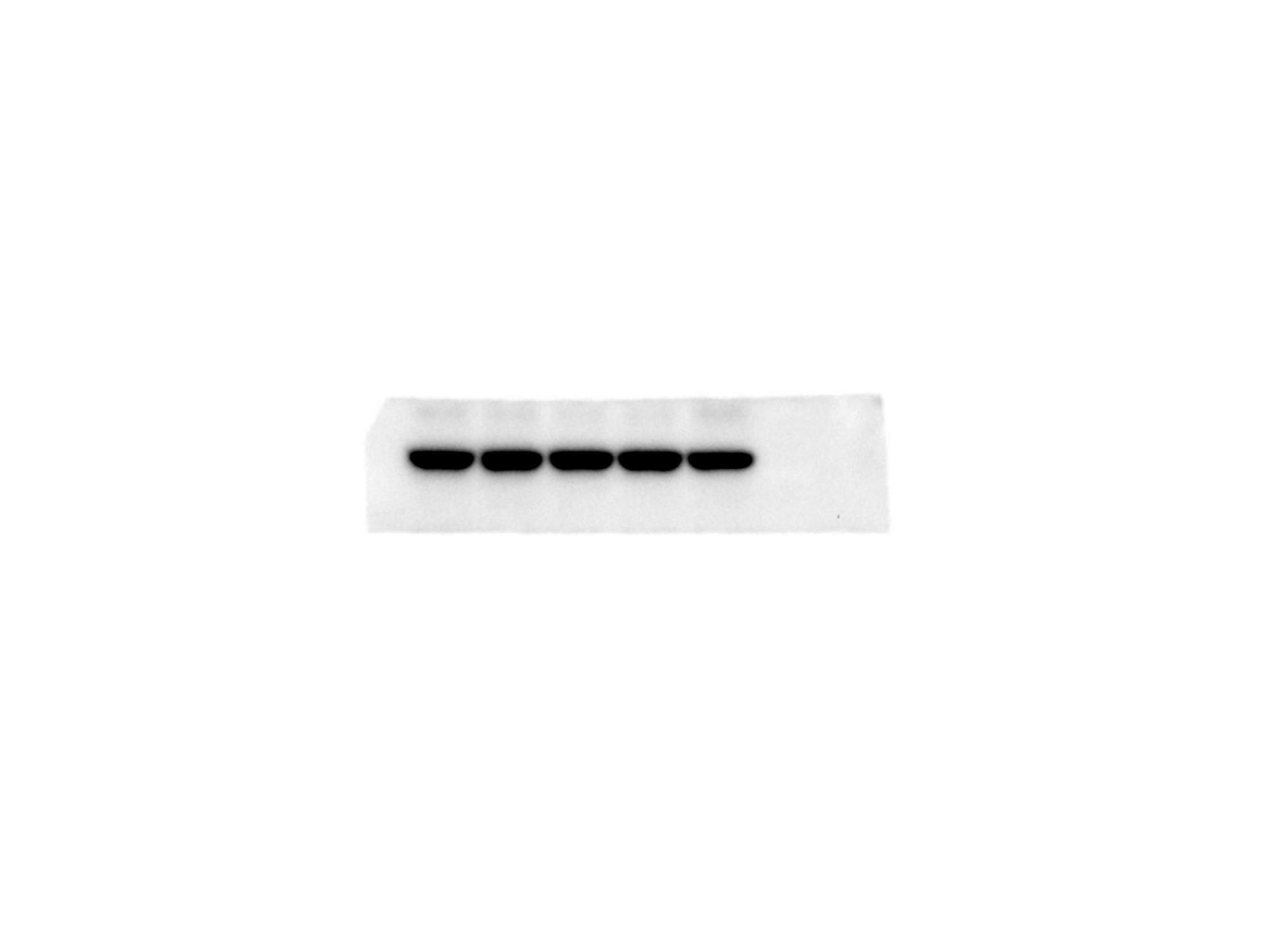

Supplement: Supplementary file 1 [file DataSheet1.ZIP › Original pictures for figures/6/6I actin.jpg]

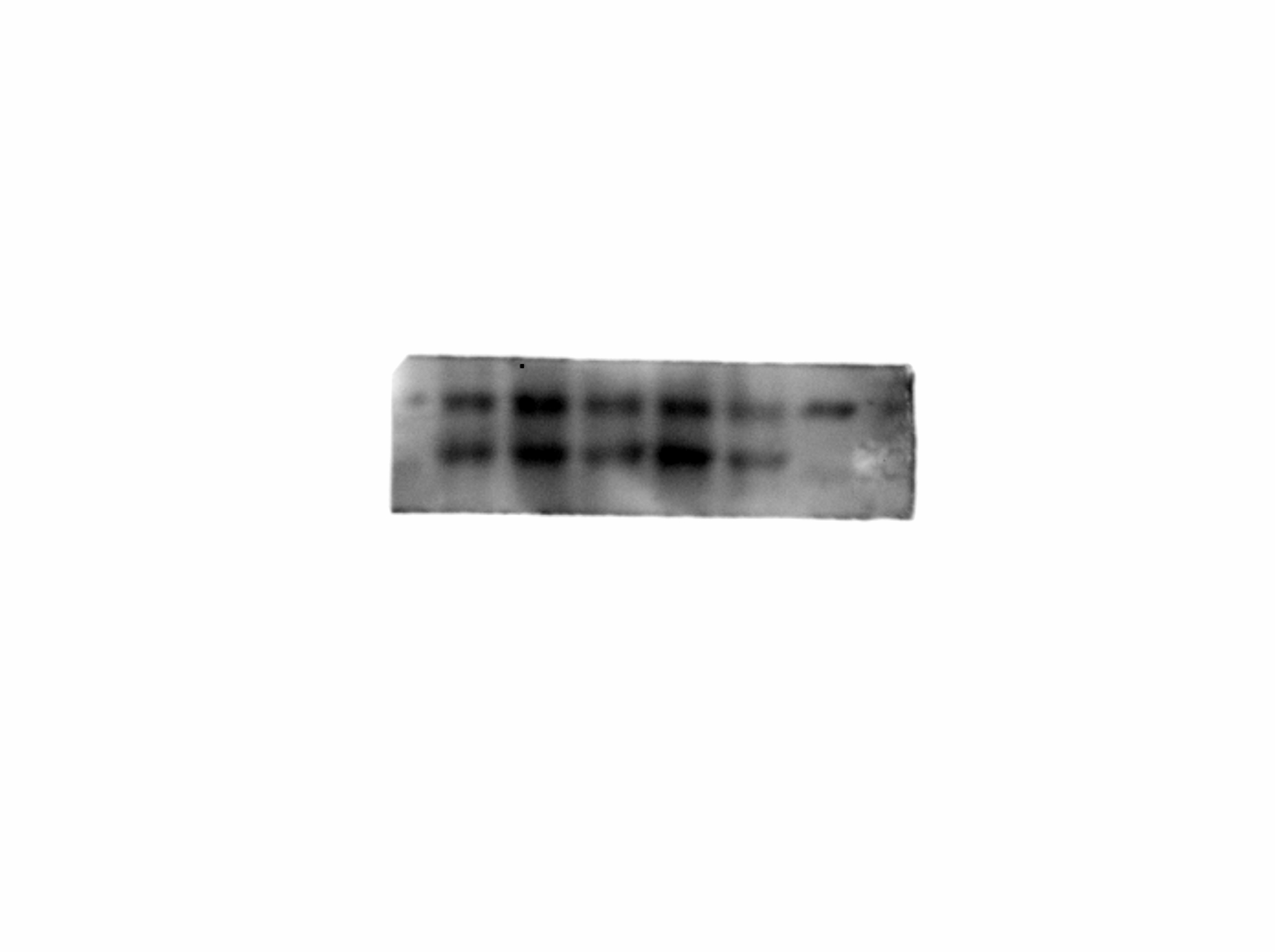

Supplement: Supplementary file 1 [file DataSheet1.ZIP › Original pictures for figures/6/6I p-JNK.jpg]

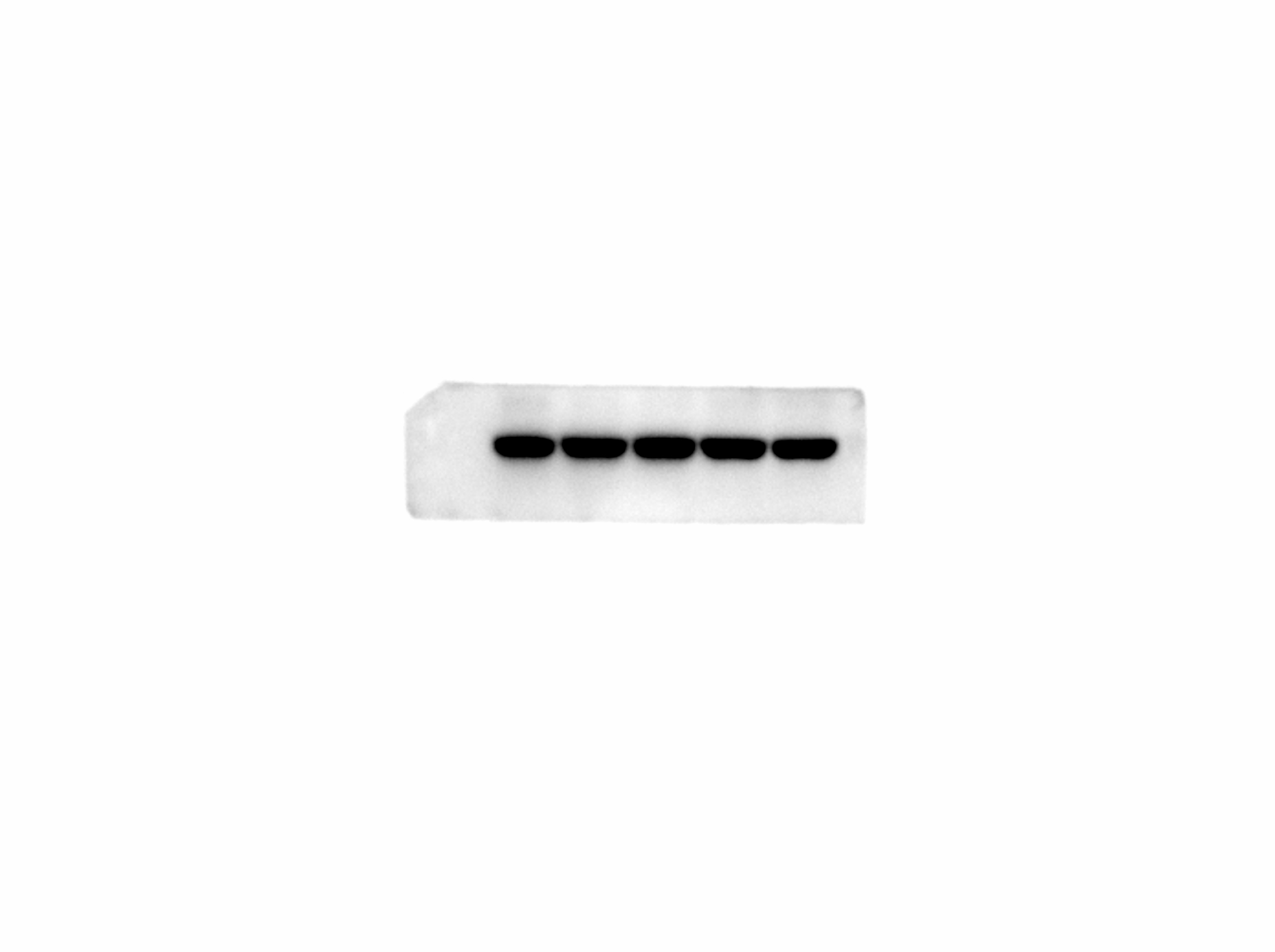

Supplement: Supplementary file 1 [file DataSheet1.ZIP › Original pictures for figures/6/6J actin.jpg]

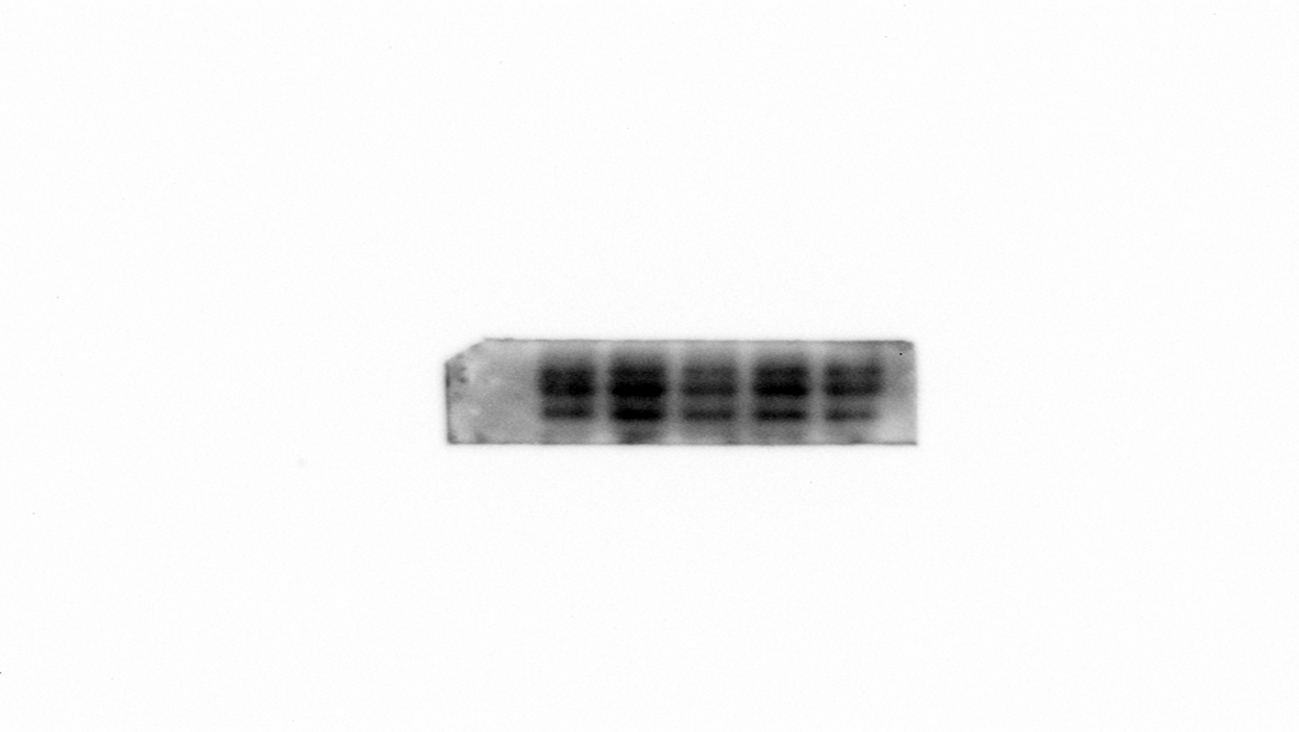

Supplement: Supplementary file 1 [file DataSheet1.ZIP › Original pictures for figures/6/6J p-Cx43.jpg]
